# *tert*-Butyl Hypochlorite: A Reagent for the Synthesis of Chlorinated Oxindole and Indole Derivatives

**DOI:** 10.3390/molecules30010102

**Published:** 2024-12-30

**Authors:** Chun-Yan Liu, Xia Chen, Hai-Long Liu, Nan Wang, Xiao-Yu Zhou

**Affiliations:** 1 College of Environmental and Chemical Engineering, Dalian University, Dalian 116622, China; lcy2403840249@126.com (C.-Y.L.); wxn001017@163.com (N.W.); 2 School of Chemistry and Materials Engineering, Liupanshui Normal University, Liupanshui 553004, China; liuhailong1988@live.cn

**Keywords:** *tert*-butyl hypochlorite, chlorooxidation, chlorination, decarboxylative chlorination, oxindole

## Abstract

*tert*-Butyl hypochlorite was employed as a versatile reagent for chlorooxidation of indoles, chlorination of 2-oxindoles, and decarboxylative chlorination of the indole-2-carboxylic acids. Four types of products including 2-chloro-3-oxindoles, 2,2-dichloro-3-oxindoles, 3,3-dichloro-2-oxindoles, and 2,3-dichloroindoles could be selectively obtained in moderate to excellent yields by switching the substrates. Various synthetically useful functional groups, such as halogen atoms, cyano, nitro, and methoxycarbonyl groups, remain intact during the reactions. Notable features of the approach include the universality of the starting materials, the mild reaction conditions, and the experimental simplicity.

## 1. Introduction

**Figure 1:**
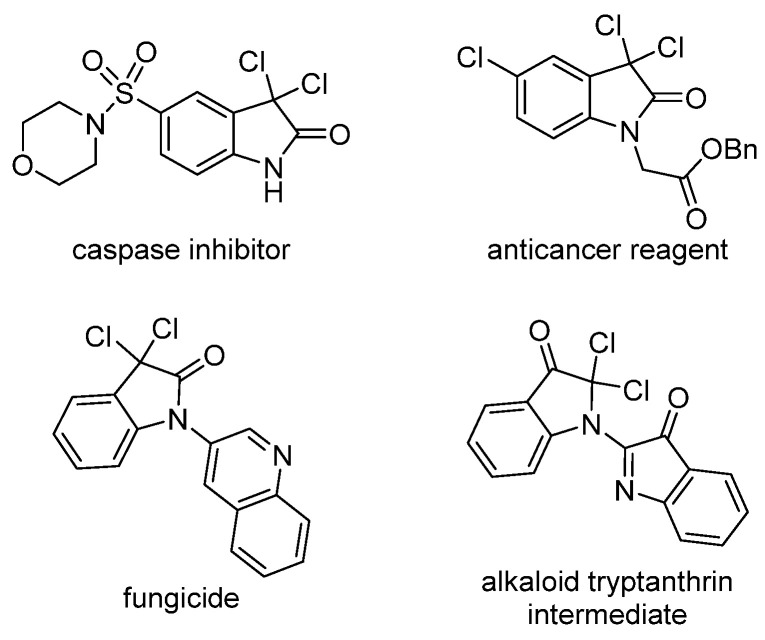
Some chlorine-containing oxindoles.

The oxindole moiety is central in a series of structurally diverse natural and man-made alkaloid products, many of which display attractive profiles for biological and pharmaceutical applications [1,2,3,4]. Chlorine-containing oxindoles are among the most prominent oxindole derivatives, owing in part to their frequent occurrence in biologically active molecules and broad application in the preparation of high-value heterocyclic molecules [5,6,7,8,9,10] (Figure 1). For example, the 3,3-dichlorooxindole scaffolds existed in caspase inhibitor, anticancer reagent, and fungicide, and 2-(2,2-dichloro-3-oxoindolin-1-yl)-3*H*-indol-3-one was a synthetic intermediate for the alkaloid tryptanthrin. Despite the significance of chlorine-containing oxindoles, reports on the synthesis of these compounds are still limited.

Conventional methods to access 3-oxindoles heavily rely on the transition metal (TM)-catalyzed tandem cyclization of complicated substrates [11,12,13,14,15,16,17,18]; moreover, these starting materials always require multi-step synthesis. Another approach is based on the oxidation of their aromatic precursors, indoles, by using oxodiperoxo molybdenum complexes [19], meta-chloroperoxybenzoic acid (*m*CPBA) [20], dimethyl dioxirane [21,22], manganese dioxide (MnO_2_) [23], hypervalent iodine [24], sodium periodate (NaIO_4_) [25] as oxidizing reagents, which represents a straightforward and practical route to obtain 2-substituted 3-oxindoles. However, the oxidation selectivity of the electron-rich indoles was still an issue, which required the installation of electron-withdrawing protective groups. Indeed, the processes always involved narrow substrate scope and low yields, and were difficult to accommodate 2-chloro-3-oxindoles preparation. As a result, it is urgent to develop convenient methods for the synthesis of 2-chloro-3-oxindoles.

**Scheme 1:**
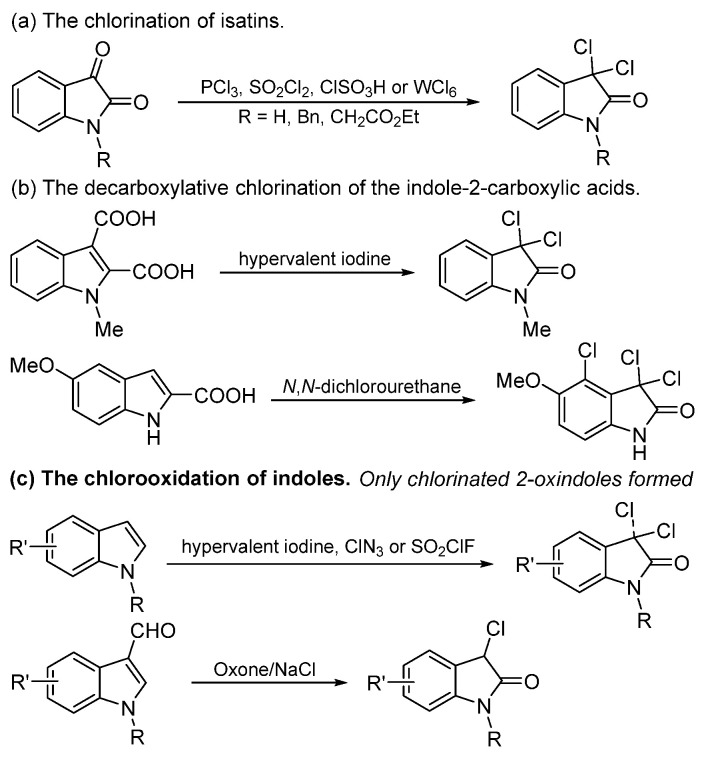
Methods for chlorinated oxindoles synthesis.

For the synthesis of 2-oxindoles, the main approach is based on the cyclization of *N*-phenyl acrylamides [26,27,28,29]. However, the strategy is associated with drawbacks such as the requirement of expensive metals and ligands, high temperatures, and toxic radical initiators. 3,3-Dichloro-2-oxindole scaffolds widely existed in a large number of biologically active compounds, with anti-diabetic, anti-cancer, and other pharmacological activities [5,30,31,32]. Accordingly, numerous chlorination methods have been successively developed to prepare 3,3-dichloro-2-oxindoles from isatins with diverse chlorine sources, such as PCl_5_ [33], SO_2_Cl_2_ [34], ClSO_3_H [35], and WCl_6_ [36] (Scheme 1a). Moreover, indole-2-carboxylic acids [37,38] and indole-2,3-dicarboxylic acids [39,40] can be converted into the corresponding 3,3-dichloro-2-oxindole, respectively (Scheme 1b). Great efforts also have been devoted to developing a direct synthesis of 3,3-dichloro-2-oxindoles through chlorooxidation reaction of indoles with chlorine azide (ClN_3_) [41], NaCl/Oxone (2KHSO_5_∙KHSO_4_∙K_2_SO_4_) [42], sulfuryl chlorofluoride (SO_2_ClF) [43], and hypervalent iodine [24,44] (Scheme 1c). Although the synthesis of various 3,3-dichloro-2-oxindoles can be achieved with the use of the methods mentioned above, the use of inexpensive, and easy-to-handle feedstocks to access 3,3-dichloro-2-oxindoles is still desirable.

**Scheme 2:**
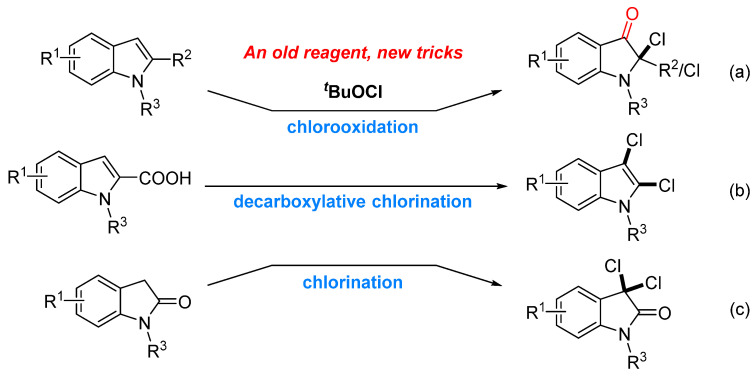
*tert*-Butyl hypochlorite for the synthesis of chlorinated oxindoles and indoles: (**a**) chlorooxidation of indoles; (**b**) decarboxylative chlorination of the indole-2-carboxylic acids; (**c**) chlorination of 2-oxindoles.

*tert*-Butyl hypochlorite (*^t^*BuOCl), a commercially available oxidating and chlorinating reagent, has been applied to various transformations like chlorination of secondary alcohols [45] and aliphatic amides [46], addition of terminal alkynes [47], and chlorooxidation of unsaturated hydrocarbons [48] and S-alkylisothiourea salts [49]. The regioselective oxidation of indoles and subsequent chlorination to construct chlorine-containing oxindoles is undoubtedly a powerful synthetic strategy because of atom economy and convenience. Herein, we would show that *^t^*BuOCl could serve as both an oxidizing reagent and chlorinating source for the controllable functionalization of protected and unprotected indoles (Scheme 2a). 

## 2. Results

In our initial studies, ethyl 1*H*-indole-2-carboxylate (**1a**) was examined as the model substrate for chlorooxidation reaction with *^t^*BuOCl (3.0 equiv.) under an air atmosphere at 60 °C for 20 h (Table 1). Ethyl 2-chloro-3-oxoindoline-2-carboxylate (**2a**) was observed in 77% yield when using tetrahydrofuran (THF) as solvent (entry 1). Among the examined solvents [THF, ethyl acetate (EtOAc), 1,4-dioxane, acetonitrile (CH_3_CN), and toluene], EtOAc proved to be the best solvent (entries 1–5). Increased yield (99%) was observed when the loading of *^t^*BuOCl was reduced to 2.5 equivalents, while decreased yields (85% and 56%) were observed when the loading was further reduced to 2.0 and 1.5 equivalent, respectively (entries 6–8 vs. entry 2). The results obtained from the experiments of temperature screening indicated that 40 °C was the best reaction temperature (entries 6 and 9–11). Almost a quantitative yield was also obtained even though the reaction time was shortened to 16 h from 20 h (entry 12 vs. entry 10), suggesting that the target reaction could be accomplished within 16 h. Therefore, the subsequent chlorooxidation reaction of various indole*s* with *^t^*BuOCl (2.5 equiv.) was performed in EtOAc at 40 °C for 16 h.

**Scheme 3:**
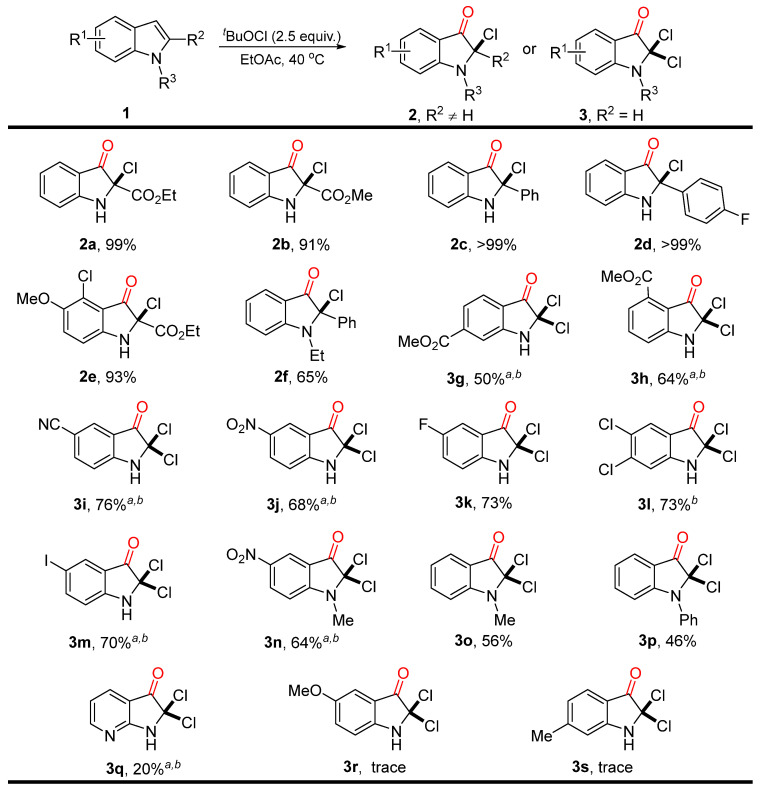
Substrate scope. Reaction conditions: **1** (0.5 mmol) and *^t^*BuOCl (2.5 equiv.) in EtOAc (3.0 mL) at 40 °C for 16 h, and isolated yield. *^a^* 4.0 equivalent of *^t^*BuOCl was used. *^b^* The reaction was performed at 60 °C.

With the optimized reaction conditions in hand, the scope and limitation of this reaction were explored, and the results are shown in Scheme 3. Reactions of indole substrates **1** bearing ethoxycarbonyl (-COOEt), methoxycarbonyl (-COOMe), and aryl linked on the pyrrole ring were investigated under the optimized reaction conditions. The desired products **2a**–**2e** were obtained in excellent yields (91%–>99%). The 1-ethyl-2-phenyl-1*H*-indole (**1f**) was employed in the reaction. The desired product **2f** was obtained in 65% yield, and the remaining starting material was decomposed. Notably, the reactions of indoles **1g** and **1h**, containing a -COOMe linked on the benzene ring, proceeded to afford the 2,2-dichloro-3-oxindole products **3g** and **3h** in 50% and 64% yields, respectively. That is, the absence of groups at the C2-position of indole would result in 2,2-dichloro-3-oxindole products. Reactions of indole substrates **1** bearing cyano (-CN) and nitro (-NO_2_) were subsequently investigated under the optimized reaction conditions. The desired products **3i** and **3j** were obtained in 76% and 68% yields. The suitability of indole substrates **1k**–**1m** with halogen atoms (-F, -Cl, and -I) in the current reaction was also investigated, and moderate yields (70–73%) of the desired products **3k**–**3m** were obtained. The functional groups, such as halogen atoms (-Cl and -I), -COOMe, -NO_2,_ and -CN remained intact during the chlorooxidation reaction of indoles, suggesting that further manipulation may produce additional useful compounds. The *N*-methyl substituted indoles **1n** and **1o** were also employed in the reaction. The desired products **3n** and **3o** were obtained in 64% and 56% yields, respectively. Moreover, *N*-phenyl indole (**1p**) reacted successfully and exhibited reactivity that was similar to *N*-Me indole. A low yield was obtained when 1*H*-pyrrolo[2,3-*b*]pyridine (**1q**) was examined, and the remaining starting material was decomposed. However, the electron-donating groups, such as methyl (Me) and methoxyl (MeO), linked on the benzene ring in substrate **1** would severely hamper the chlorooxidation reaction. Only trace amounts of the desired products **3r** and **3s** were obtained, and most of the starting materials were decomposed. The reason behind this may be that an indole with an electron-donor group easily undergoes an oxidation reaction, leading to the decomposition of the starting materials.

**Scheme 4:**
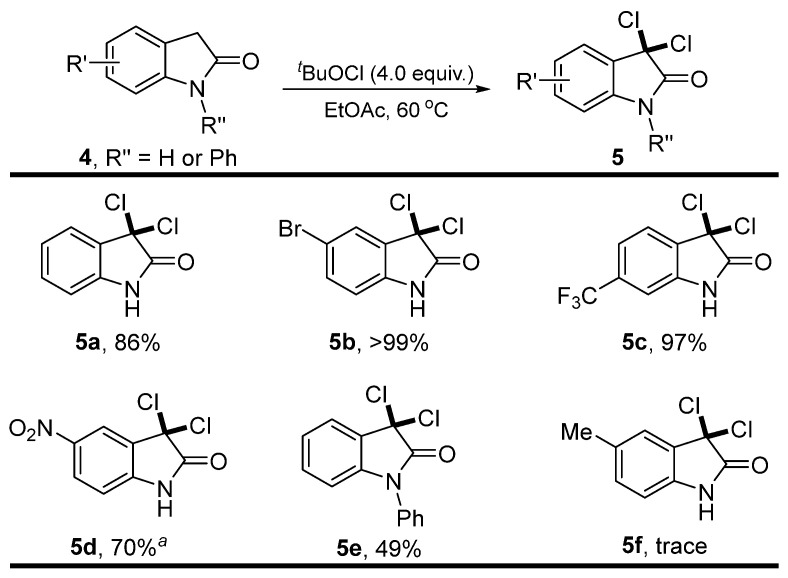
Substrate scope. Reaction conditions: **4** (0.5 mmol) and *^t^*BuOCl (4.0 equiv.) in EtOAc (3.0 mL) at 60 °C for 24 h, and isolated yield. *^a^* The reaction was performed at 80 °C.

Given that *^t^*BuOCl is a readily available and controllable chlorinating source, exploring its new applications is still interesting. Herein, we would further show that *^t^*BuOCl could serve as a chlorinating reagent for the functionalization of 2-oxindoles, and 3,3-dichloro-2-oxindoles were selectively formed (Scheme 2c). At the outset, we chose the simple unprotected 2-oxindole (**4a**) as the model substrate to investigate the reactivity of *^t^*BuOCl under the above-mentioned conditions (in EtOAc at 40 °C). The reaction parameters, including the loading of *^t^*BuOCl, temperature, and reaction time, were screened (for details, see Table S1). The substrate scope of the 2-oxindoles was investigated under optimized reaction conditions: *^t^*BuOCl (4.0 equiv.) in EtOAc (3.0 mL) at 60 °C for 16 h. The results are shown in Scheme 4.

Reactions of 2-oxindole and its derivatives bearing bromine (-Br), trifluoromethyl (-CF_3_), or -NO_2_ on the benzene ring proceeded smoothly to afford the target products **5a**–**5d**; except for **5d** (70%), good to excellent yields (86%–>99%) of the desired products were obtained. The *N*-phenyl substituted 2-oxindoles (**4e**) was employed in the reaction. The desired product **5e** was obtained in a 49% yield, and the starting material was recovered. The reaction of 5-methylindolin-2-one (**4f**) was also examined, but only trace amounts of the desired product **5f** were detected.

**Scheme 5:**
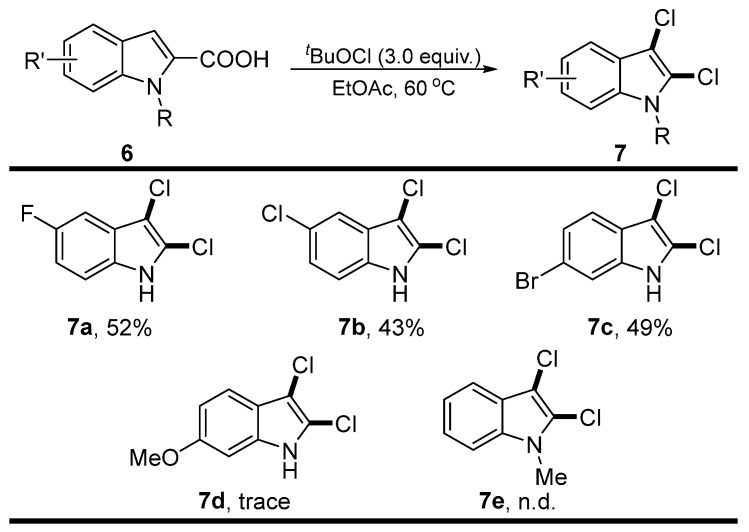
Substrate scope. Reaction conditions: **6** (0.5 mmol) and *^t^*BuOCl (3.0 equiv.) in EtOAc (3.0 mL) at 60 °C for 24 h, and isolated yield.

Reactions of indole-2-carboxylic acid derivatives **6a**–**6d** were performed to further expand the substrate scope (Scheme 5). Interestingly, the corresponding decarboxylative chlorination products **7a**–**7d** were obtained instead of the 2-chloro-3-oxindoles. Except for **7d**, moderate yields (43%–>52%) of the products were obtained, and the remaining starting materials were decomposed. The 1-methylindole-2-carboxylic acid (**6e**) was also examined in the reaction. No desired product was observed, and most of the starting materials were recovered. Notably, 2,3-dichloroindoles are also prominent due to their broad application in the preparation of high-value heterocyclic molecules.

**Scheme 6:**
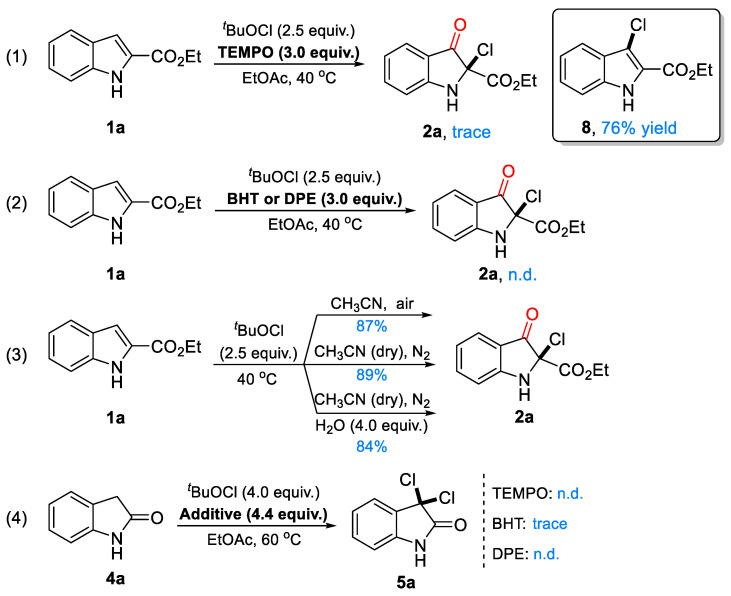
The control experiments.

The reactions with different free radical trapping reagents, including 2,2,6,6-tetramethyl-1-piperidinyloxy (TEMPO), butylated hydroxytoluene (BHT), and 1,1-diphenylethylene (DPE) were performed to gain insights into the reaction pathway (Scheme 6(1),(2),(4)). Almost completely inhibited reactions were attained, clearly indicating that the chlorooxidation of indoles and the chlorination of 2-oxindoles possibly proceeded via a radical pathway. Moreover, ethyl 3-chloro-indole-2-carboxylate (**8**) was isolated in a 76% yield when **1a** was treated in the presence of *^t^*BuOCl and TEMPO. The reason behind this may be that *^t^*BuOCl could provide Cl^+^ as an electrophilic reagent and undergo an electrophilic substitution reaction with **1a**. On the other hand, the final product **2a** was obtained in 89% yield almost as a sole product in the dry CH_3_CN under a N_2_ atmosphere, suggesting that the oxygen source in **2a** possibly came from *^t^*BuOCl, rather than H_2_O and air (Scheme 6(3)).

**Scheme 7:**
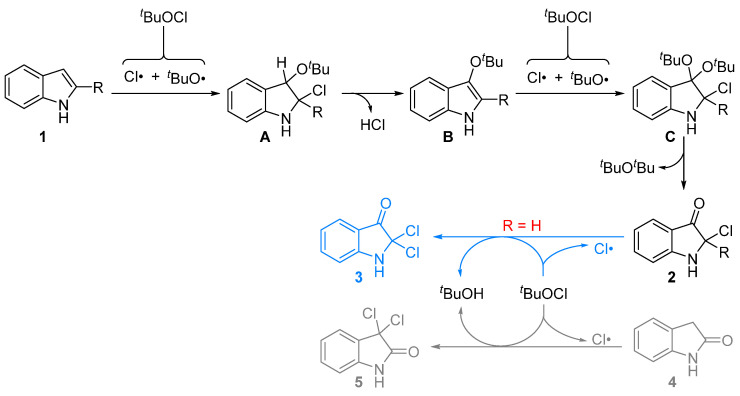
The plausible reaction mechanism.

On the basis of our experimental outcomes, a plausible reaction mechanism for the chlorooxidation of indoles is depicted in Scheme 7. The homolytic cleavage of the O–Cl bond of *^t^*BuOCl occurred and produced chlorine and oxygen radicals, which would react with **1** to provide the adduct **A**. The subsequent elimination reaction of **A** generated intermediate **B**. Acetal **C** was formed through the radical addition reaction of **B** with another *^t^*BuOCl. Elimination reaction of **C** would occur to furnish the desired product **2**. 2-Chloro-3-oxindole and 2-oxindole, in the presence of *α*-H of ketone, further underwent radical chlorination to generate 2,2-dichloro-3-oxindoles (**3**) and 3,3-dichloro-2-oxindoles (**5**).

## 3. Conclusions

In summary, we have disclosed that *^t^*BuOCl plays a dual role as an oxidizing reagent and chlorinating source for the chlorooxidation of indoles and chlorination of 2-oxindoles. This approach enables access to various 2-chloro-3-oxindoles, 2,2-dichloro-3-oxindoles, and 3,3-dichloro-2-oxindoles in a controllable manner by choosing different substrates. The decarboxylative chlorination of indole-2-carboxylic acids with *^t^*BuOCl also proceeded smoothly to produce 2,3-dichloroindoles in moderate yields. Different groups, including synthetically useful functional groups -F, -Cl, -Br, -CO_2_Me, -CN, and -NO_2_ are well tolerated under the reactions. The mild conditions, simplicity, and universality of the starting materials and the experimental simplicity make the present methodology highly useful in the synthesis of chlorinated oxindoles and indoles.

## 4. Materials and Methods

### 4.1. General Information

Unless otherwise noted, all reactions were carried out in oven-dried 25 mL Schlenk tubes under an air atmosphere. IKA plate was used as the heat source. All reagents and solvents were of pure analytical grade. Thin layer chromatography (TLC) was performed on HSGF254 silica gel, pre-coated on glass-backed plates coated with 0.2 mm silica and revealed with either a UV lamp (λ_max_ = 254 nm). The products were purified by flash column chromatography on silica gel 200–300 mesh. ^1^H, ^13^C, and ^19^F NMR spectra were recorded on a Bruker Avance NEO 600M NMR Spectrometer (600 MHz for ^1^H, 151 MHz for ^13^C, 565 MHz for ^19^F), a Bruker Avance III HD 500M NMR Spectrometer (500 MHz for ^1^H, 126 MHz for ^13^C, 471 MHz for ^19^F), using CDCl_3_ or *d*_6_-DMSO as the solvent with tetramethylsilane (TMS) as the internal standard at room temperature. The chemical shifts are reported in ppm downfield (*δ*) from TMS, and the coupling constants *J* are given in Hz. The peak patterns are indicated as follows: s, singlet; d, doublet; t, triplet; q, quartet; m, multiplet. High-resolution mass spectra were recorded on either a Q-TOF mass spectrometry or an LTQ Orbitrap XL mass spectrometry. Unless otherwise noted, starting materials are commercially available. Unless otherwise noted, starting materials are commercially available.

### 4.2. Synthetic Procedures

#### 4.2.1. The Typical Procedure for Chlorooxidation of Indoles with *^t^*BuOCl

To an oven-dried 25 mL Schlenk tube equipped with a magnetic stir bar, indole substrate **1** (0.5 mmol, 1.0 equiv.), *^t^*BuOCl (1.25 mmol, 2.5 equiv.) or *^t^*BuOCl (2.0 mmol, 4.0 equiv.), and EtOAc (3.0 mL) were added and sealed under an air atmosphere. The reaction mixture was stirred at 40 °C for 16 h, and then cooled to room temperature. The solvent was removed under reduced pressure and the crude product was purified by silica gel column chromatography to afford the desired product **2** or **3**.

**ethyl 2-chloro-3-oxoindoline-2-carboxylate (2a):** Yield: 99%, 118.6 mg, white solid, mp 121–123 °C, purified using petroleum ether/ethyl acetate (5:1). ^1^H NMR (600 MHz, CDCl_3_) *δ* 9.28 (s, 1H), 7.45 (d, *J* = 7.6 Hz, 1H), 7.38–7.35 (m, 1H), 7.15–7.12 (m, 1H), 7.03 (d, *J* = 7.8 Hz, 1H), 4.35–4.23 (m, 2H), 1.26 (t, *J* = 7.1 Hz, 3H). ^13^C{^1^H} NMR (151 MHz, CDCl_3_) *δ* 172.4, 165.0, 140.9, 131.4, 127.0, 125.0, 123.8, 111.2, 64.7, 63.8, 13.9. HRMS (ESI) *m*/*z*: [M+H]^+^ Calcd for C_11_H_11_ClNO_3_^+^ 240.0422; Found 240.0425.

**methyl 2-chloro-3-oxoindoline-2-carboxylate (2b):** Yield: 91%, 102.6 mg, white solid, mp 117–119 °C, purified using petroleum ether/ethyl acetate (5:1). ^1^H NMR (600 MHz, CDCl_3_) *δ* 9.31 (brs, 1H), 7.45 (d, *J* = 7.6 Hz, 1H), 7.39–7.36 (m, 1H), 7.15–7.13 (m, 1H), 7.03 (d, *J* = 7.8 Hz, 1H), 3.84 (s, 3H). ^13^C{^1^H} NMR (151 MHz, CDCl_3_) *δ* 172.3, 165.6, 140.9, 131.5, 126.8, 125.1, 123.9, 111.3, 64.6, 54.4. HRMS (ESI) *m*/*z*: [M+H]^+^ Calcd for C_10_H_9_ClNO_3_^+^ 226.0265; Found 226.0267.

**2-chloro-2-phenylindolin-3-one (2c):** Yield: >99%, 121.0 mg, yellow solid, mp 130–132 °C, purified using petroleum ether/ethyl acetate (10:1~5:1). ^1^H NMR (600 MHz, CDCl_3_) *δ* 8.53–8.45 (m, 2H), 7.76 (d, *J* = 7.4 Hz, 1H), 7.65 (d, *J* = 7.7 Hz, 1H), 7.62–7.56 (m, 3H), 7.51–7.49 (m, 1H), 7.43–7.38 (m, 1H). ^13^C{^1^H} NMR (151 MHz, CDCl_3_) *δ* 171.9, 149.3, 139.8, 132.1, 131.6, 129.5, 128.7, 128.6, 127.9, 122.7, 121.6, 80.2. HRMS (ESI) *m*/*z*: [M+H]^+^ Calcd for C_14_H_11_ClNO^+^ 244.0524; Found 244.0526.

**2-chloro-2-(4-fluorophenyl)indolin-3-one (2d):** Yield: >99%, 130.5 mg, yellow solid, mp 123–125 °C, purified using petroleum ether/ethyl acetate (10:1~5:1). ^1^H NMR (600 MHz, CDCl_3_) *δ* 8.50–8.47 (m, 2H), 7.75 (d, *J* = 7.3 Hz, 1H), 7.63 (d, *J* = 7.7 Hz, 1H), 7.51–7.48 (m, 1H), 7.43–7.37 (m, 1H), 7.28–7.21 (m, 2H). ^13^C{^1^H} NMR (151 MHz, CDCl_3_) *δ* 170.8, 165.1 (d, *J*_C-F_ = 254.9 Hz), 149.2, 139.7, 131.8 (d, *J*_C-F_ = 8.7 Hz), 131.6, 127.9, 124.9 (d, *J*_C-F_ = 3.2 Hz), 122.8, 121.5, 115.9 (d, *J*_C-F_ = 22.1 Hz), 80.1. ^19^F NMR (565 MHz, CDCl_3_) *δ* -106.2 (s, 1F). HRMS (ESI) *m*/*z*: [M+H]^+^ Calcd for C_14_H_10_ClFNO^+^ 262.0429; Found 262.0431.

**ethyl 2,4-dichloro-5-methoxy-3-oxoindoline-2-carboxylate (2e):** Yield: 93%, 142.1 mg, white solid, mp 130–132 °C, purified using petroleum ether/ethyl acetate (5:1). ^1^H NMR (600 MHz, CDCl_3_) *δ* 9.17 (1H), 6.95–6.91 (m, 2H), 4.38–4.28 (m, 2H), 3.91 (s, 3H), 1.27 (t, *J* = 7.1 Hz, 3H). ^13^C{^1^H} NMR (151 MHz, CDCl_3_) *δ* 171.3, 163.4, 152.0, 134.9, 126.9, 121.2, 113.9, 109.6, 65.8, 64.1, 56.8, 14.0. HRMS (ESI) *m*/*z*: [M+H]^+^ Calcd for C_12_H_12_Cl_2_NO_4_^+^ 304.0138; Found 304.0141.

**2-chloro-1-ethyl-2-phenylindolin-3-one (2f):** Yield: 65%, 88.6 mg, white solid, mp 90–92 °C, purified using petroleum ether. ^1^H NMR (500 MHz, CDCl_3_) *δ* 7.65–7.53 (m, 5H), 7.53–7.44 (m, 3H), 7.27 (d, *J* = 1.9 Hz, 1H), 4.45 (q, *J* = 7.0 Hz, 2H), 1.20 (t, *J* = 7.1 Hz, 3H). ^13^C{^1^H} NMR (126 MHz, CDCl_3_) *δ* 139.4, 130.6, 129.6, 129.4, 129.31, 129.26, 128.7, 126.0, 124.5, 117.5, 116.8, 104.6, 41.0, 17.1. HRMS (ESI) *m*/*z*: [M+H]^+^ Calcd for C_16_H_15_ClNO^+^ 272.0837; Found 272.0841.

**methyl 2,2-dichloro-3-oxoindoline-6-carboxylate (3g):** Yield: 50%, 65.6 mg, white solid, mp 178–180 °C, purified using petroleum ether/ethyl acetate (2:1). ^1^H NMR (500 MHz, *d*_6_-DMSO) *δ* 11.56 (s, 1H), 7.90–7.68 (m, 2H), 7.44 (s, 1H), 3.87 (s, 3H). ^13^C{^1^H} NMR (126 MHz, *d*_6_-DMSO) *δ* 169.2, 165.6, 140.0, 133.5, 125.6, 125.2, 111.8, 74.6, 53.1. HRMS (ESI) *m*/*z*: [M+H]^+^ Calcd for C_10_H_8_Cl_2_NO_3_^+^ 259.9876; Found 259.9878.

**methyl 2,2-dichloro-3-oxoindoline-4-carboxylate (3h):** Yield: 64%, 82.7 mg, light yellow solid, mp 136–138 °C, purified using petroleum ether/ethyl acetate (2:1). ^1^H NMR (500 MHz, *d*_6_-DMSO) *δ* 11.55 (s, 1H), 7.89–7.58 (m, 2H), 7.25–7.23 (m, 1H), 3.92 (s, 3H). ^13^C{^1^H} NMR (126 MHz, *d*_6_-DMSO) *δ* 169.6, 164.6, 141.2, 133.2, 128.0, 127.6, 125.3, 116.1, 75.3, 52.8. HRMS (ESI) *m*/*z*: [M+H]^+^ Calcd for C_10_H_8_Cl_2_NO_3_^+^ 259.9876; Found 259.9877.

**2,2-dichloro-3-oxoindoline-5-carbonitrile (3i):** Yield: 76%, 86.7 mg, brown solid, mp 212–214 °C, purified using petroleum ether/ethyl acetate (2:1). ^1^H NMR (500 MHz, *d*_6_-DMSO) *δ* 11.86 (s, 1H), 8.24 (s, 1H), 7.86 (d, *J* = 4.9 Hz, 1H), 7.13 (d, *J* = 5.5 Hz, 1H). ^13^C{^1^H} NMR (126 MHz, *d*_6_-DMSO) *δ* 169.4, 143.8, 137.7, 130.2, 129.3, 118.6, 112.8, 106.4, 73.9. HRMS (ESI) *m*/*z*: [M+H]^+^ Calcd for C_9_H_5_Cl_2_N_2_O^+^ 226.9773; Found 226.9772.

**2,2-dichloro-5-nitroindolin-3-one (3j):** Yield: 68%, 84.2 mg, yellow solid, mp 154–156 °C, purified using petroleum ether/ethyl acetate (2:1). ^1^H NMR (500 MHz, *d*_6_-DMSO) *δ* 12.04 (s, 1H), 8.43 (s, 1H), 8.30 (s, 1H), 7.17 (s, 1H). ^13^C{^1^H} NMR (126 MHz, *d*_6_-DMSO) *δ* 169.7, 145.6, 143.8, 129.8, 129.3, 120.9, 112.5, 73.8. HRMS (ESI) *m*/*z*: [M+H]^+^ Calcd for C_8_H_5_Cl_2_N_2_O_3_^+^ 246.9672; Found 246.9675.

**2,2-dichloro-5-fluoroindolin-3-one (3k):** Yield: 73%, 80.3 mg, white solid, mp 156–158 °C, purified using petroleum ether/ethyl acetate (10:1~5:1). ^1^H NMR (500 MHz, CDCl_3_) *δ* 9.54 (s, 1H), 7.37 (dd, *J* = 7.2, 2.5 Hz, 1H), 7.14–7.10 (m, 1H), 7.03 (dd, *J* = 8.6, 4.1 Hz, 1H). ^13^C{^1^H} NMR (126 MHz, CDCl_3_) *δ* 171.45, 159.7 (d, *J*_C-F_ = 244.9 Hz), 133.9 (d, *J*_C-F_ = 2.3 Hz), 130.8 (d, *J*_C-F_ = 8.6 Hz), 118.9 (d, *J*_C-F_ = 23.8 Hz), 112.8 (d, *J*_C-F_ = 29.1 Hz), 112.7 (d, *J*_C-F_ = 4.6 Hz), 74.3. ^19^F NMR (471 MHz, CDCl_3_) *δ* -116.8 (s, 1F). HRMS (ESI) *m*/*z*: [M+H]^+^ Calcd for C_8_H_5_Cl_2_FNO^+^ 219.9727; Found 219.9730.

**2,2,5,6-tetrachloroindolin-3-one (3l):** Yield: 73%, 98.5 mg, yellow solid, mp 200–202 °C, purified using petroleum ether/ethyl acetate (10:1~5:1). ^1^H NMR (500 MHz, *d*_6_-DMSO) *δ* 11.64 (s, 1H), 8.04 (s, 1H), 7.20 (s, 1H). ^13^C{^1^H} NMR (126 MHz, *d*_6_-DMSO) *δ* 169.2, 139.6, 135.3, 129.5, 127.3, 126.2, 113.7, 74.1. HRMS (ESI) *m*/*z*: [M+H]^+^ Calcd for C_8_H_4_Cl_4_NO^+^ 269.9042; Found 269.9041.

**2,2-dichloro-5-iodoindolin-3-one (3m):** Yield: 70%, 114.9 mg, yellow solid, mp 230–232 °C, purified using petroleum ether/ethyl acetate (10:1~5:1). ^1^H NMR (500 MHz, *d*_6_-DMSO) *δ* 11.46 (s, 1H), 7.97 (s, 1H), 7.75 (d, *J* = 7.9 Hz, 1H), 6.82 (d, *J* = 8.1 Hz, 1H). ^13^C{^1^H} NMR (126 MHz, *d*_6_-DMSO) *δ* 169.0, 141.4, 139.3, 133.4, 131.4, 114.1, 86.6, 74.6. HRMS (ESI) *m*/*z*: [M+H]^+^ Calcd for C_8_H_5_Cl_2_INO^+^ 327.8787; Found 327.8789.

**2,2-dichloro-1-methyl-5-nitroindolin-3-one (3n):** Yield: 64%, 83.0 mg, yellow solid, mp 184–186 °C, purified using petroleum ether/ethyl acetate (5:1). ^1^H NMR (500 MHz, CDCl_3_) *δ* 8.50 (s, 1H), 8.40–8.35 (m, 1H), 7.06 (d, *J* = 8.7 Hz, 1H), 3.38 (s, 3H). ^13^C{^1^H} NMR (126 MHz, CDCl_3_) *δ* 168.7, 146.0, 144.4, 129.9, 128.4, 120.9, 109.4, 72.4, 27.6. HRMS (ESI) *m*/*z*: [M+H]^+^ Calcd for C_9_H_7_Cl_2_N_2_O_3_^+^ 260.9828; Found 260.9827.

**2,2-dichloro-1-methylindolin-3-one (3o):** Yield: 56%, 60.3 mg, white solid, mp 76–78 °C, purified using petroleum ether/ethyl acetate (10:1). ^1^H NMR (600 MHz, CDCl_3_) *δ* 7.66 (d, *J* = 7.2 Hz, 1H), 7.45–7.42 (m, 1H), 7.23–7.20 (m, 1H), 6.89 (d, *J* = 7.9 Hz, 1H), 3.30 (s, 3H). ^13^C{^1^H} NMR (151 MHz, CDCl_3_) *δ* 169.0, 140.7, 131.9, 129.3, 124.8, 124.3, 109.2, 74.3, 27.1. HRMS (ESI) *m*/*z*: [M+H]^+^ Calcd for C_9_H_8_Cl_2_NO^+^ 215.9977; Found 215.9978.

**2,2-dichloro-1-phenylindolin-3-one (3p):** Yield: 46%, 64.2 mg, white solid, mp 86–88 °C, purified using petroleum ether/ethyl acetate (10:1). ^1^H NMR (600 MHz, CDCl_3_) *δ* 7.74 (d, *J* = 7.6 Hz, 1H), 7.60–7.57 (m, 2H), 7.5 –7.45 (m, 3H), 7.38–7.35 (m, 1H), 7.29–7.24 (m, 1H), 6.85 (d, *J* = 8.0 Hz, 1H). ^13^C{^1^H} NMR (151 MHz, CDCl_3_) *δ* 168.1, 140.8, 133.0, 131.8, 129.8, 129.0, 128.9, 126.3, 125.1, 124.6, 110.3, 74.5. HRMS (ESI) *m*/*z*: [M+H]^+^ Calcd for C_14_H_10_Cl_2_NO^+^ 278.0134; Found 278.0136.

**2,2-dichloro-1,2-dihydro-3*H*-pyrrolo[2,3-*b*]pyridin-3-one (3q):** Yield: 20%, 20.8 mg, white solid, mp 160–162 °C, purified using petroleum ether/ethyl acetate (2:1). ^1^H NMR (500 MHz, CDCl_3_) *δ* 9.65 (s, 1H), 8.35 (d, *J* = 4.2 Hz, 1H), 7.95 (d, *J* = 7.3 Hz, 1H), 7.21–7.18 (m, 1H). ^13^C{^1^H} NMR (126 MHz, CDCl_3_) *δ* 168.8, 153.3, 149.5, 133.8, 125.0, 119.8, 73.1. HRMS (ESI) *m*/*z*: [M+H]^+^ Calcd for C_7_H_5_Cl_2_N_2_O^+^ 202.9773; Found 202.9776.

#### 4.2.2. The Typical Procedure for Chlorination of 2-Oxindoles with *^t^*BuOCl

To an oven-dried 25 mL Schlenk tube equipped with a magnetic stir bar, indole substrate **4** (0.5 mmol, 1.0 equiv.), *^t^*BuOCl (2.0 mmol, 4.0 equiv.), and EtOAc (3.0 mL) were added and sealed under an air atmosphere. The reaction mixture was stirred at 60 °C for 24 h, and then cooled to room temperature. The solvent was removed under reduced pressure and the crude product was purified by silica gel column chromatography to afford the desired product **5**.

**3,3-dichloroindolin-2-one (5a):** Yield: 86%, 87.3 mg, white solid, mp 176–178 °C, purified using petroleum ether/ethyl acetate (5:1). ^1^H NMR (500 MHz, *d*_6_-DMSO) *δ* 11.47 (s, 1H), 7.74 (s, 1H), 7.44 (d, *J* = 8.5 Hz, 1H), 6.98 (d, *J* = 8.6 Hz, 1H). ^13^C{^1^H} NMR (126 MHz, *d*_6_-DMSO) *δ* 169.3, 138.5, 132.8, 130.9, 128.0, 125.3, 113.4, 74.8. HRMS (ESI) *m*/*z*: [M+H]^+^ Calcd for C_8_H_6_Cl_2_NO^+^ 201.9821; Found 201.9822.

**5-bromo-3,3-dichloroindolin-2-one (5b):** Yield: >99%, 140.1 mg, white solid, mp 198–200 °C, purified using petroleum ether/ethyl acetate (5:1). ^1^H NMR (500 MHz, *d*_6_-DMSO) *δ* 11.49 (s, 1H), 7.87 (s, 1H), 7.59 (d, *J* = 8.3 Hz, 1H), 6.94 (d, *J* = 8.4 Hz, 1H). ^13^C{^1^H} NMR (126 MHz, *d*_6_-DMSO) *δ* 169.2, 138.9, 135.6, 131.2, 128.0, 115.4, 113.9, 74.7. HRMS (ESI) *m*/*z*: [M+H]^+^ Calcd for C_8_H_5_BrCl_2_NO^+^ 279.8926; Found 279.8925.

**3,3-dichloro-6-(trifluoromethyl)indolin-2-one (5c):** Yield: 97%, 131.4 mg, white solid, mp 156–158 °C, purified using petroleum ether/ethyl acetate (5:1). ^1^H NMR (500 MHz, *d*_6_-DMSO) *δ* 11.73 (s, 1H), 7.91 (d, *J* = 7.9 Hz, 1H), 7.53 (d, *J* = 7.9 Hz, 1H), 7.23 (s, 1H). ^13^C{^1^H} NMR (126 MHz, *d*_6_-DMSO) *δ* 169.2, 140.6, 133.1, 132.7 (q, *J*_C-F_ = 30.2 Hz), 126.3, 123.8 (q, *J*_C-F_ = 273.4 Hz), 121.0 (q, *J*_C-F_ = 3.9 Hz), 108.4 (q, *J*_C-F_ = 4.0 Hz), 74.2. ^19^F NMR (471 MHz, *d*_6_-DMSO) *δ* -61.8 (s, 3F). HRMS (ESI) *m*/*z*: [M+H]^+^ Calcd for C_9_H_5_Cl_2_F_3_NO^+^ 269.9695; Found 269.9693.

**3,3-dichloro-5-nitroindolin-2-one (5d):** Yield: 70%, 86.5 mg, yellow solid, mp 172–174 °C, purified using petroleum ether/ethyl acetate (5:1). ^1^H NMR (500 MHz, *d*_6_-DMSO) *δ* 12.04 (s, 1H), 8.45 (s, 1H), 8.32 (d, *J* = 8.7 Hz, 1H), 7.18 (d, *J* = 8.7 Hz, 1H). ^13^C{^1^H} NMR (126 MHz, *d*_6_-DMSO) *δ* 169.7, 145.6, 143.8, 129.8, 129.4, 121.0, 112.5, 73.8. HRMS (ESI) *m*/*z*: [M+H]^+^ Calcd for C_8_H_5_Cl_2_N_2_O_3_^+^ 246.9672; Found 246.9673.

**3,3-dichloro-1-phenylindolin-2-one (5e):** Yield: 49%, 67.5 mg, yellow oil, purified using petroleum ether/ethyl acetate (10:1). ^1^H NMR (500 MHz, CDCl_3_) *δ* 7.78–7.69 (m, 1H), 7.60–7.57 (m, 2H), 7.52–7.44 (m, 3H), 7.39–7.31 (m, 1H), 7.26–7.23 (m, 1H), 6.82 (dd, *J* = 26.8, 8.2 Hz, 1H). ^13^C{^1^H} NMR (126 MHz, CDCl_3_) *δ* 168.1, 131.8, 130.1, 129.9, 129.2, 129.0, 126.4, 126.3, 125.5, 125.2, 124.7, 111.6, 110.4, 74.6. HRMS (ESI) *m*/*z*: [M+H]^+^ Calcd for C_14_H_10_Cl_2_NO^+^ 278.0134; Found 278.0135.

#### 4.2.3. The Typical Procedure for Decarboxylative Chlorination of Indole-2-Carboxylic Acids

To an oven-dried 25 mL Schlenk tube equipped with a magnetic stir bar, indole-2-carboxylic acid substrate **6** (0.5 mmol, 1.0 equiv.), *^t^*BuOCl (1.5 mmol, 3.0 equiv.), and EtOAc (3.0 mL) were added and sealed under an air atmosphere. The reaction mixture was stirred at 60 °C for 24 h, and then cooled to room temperature. The solvent was removed under reduced pressure and the crude product was purified by silica gel column chromatography to afford the desired product **7**.

**2,3-dichloro-5-fluoro-1*H*-indole (7a):** Yield: 52%, 52.5 mg, white solid, mp 131–133 °C, purified using petroleum ether/ethyl acetate (10:1). ^1^H NMR (600 MHz, CDCl_3_) *δ* 8.14 (brs, 1H), 7.27–7.18 (m, 2H), 7.03–7.00 (m, 1H). ^13^C{^1^H} NMR (151 MHz, CDCl_3_) *δ* 158.5 (d, *J*_C-F_ = 238.1 Hz), 129.6, 126.1 (d, *J*_C-F_ = 10.7 Hz), 121.7, 112.0 (d, *J*_C-F_ = 20.9 Hz), 111.9 (d, *J*_C-F_ = 3.7 Hz), 103.9 (d, *J*_C-F_ =4.5 Hz), 103.3 (d, *J*_C-F_ = 25.8 Hz). ^19^F NMR (565 MHz, CDCl_3_) *δ* -121.7. HRMS (ESI) *m*/*z*: [M+H]^+^ Calcd for C_8_H_5_Cl_2_FN^+^ 203.9778; Found 203.9780.

**2,3,5-trichloro-1*H*-indole (7b):** Yield: 43%, 47.0 mg, white solid, mp 148–150 °C, purified using petroleum ether/ethyl acetate (10:1). ^1^H NMR (600 MHz, CDCl_3_) *δ* 8.18 (brs, 1H), 7.54 (s, 1H), 7.24–7.19 (m, 2H). ^13^C{^1^H} NMR (151 MHz, CDCl_3_) *δ* 131.5, 127.1, 126.5, 123.9, 121.5, 117.5, 112.0, 103.5. HRMS (ESI) *m*/*z*: [M+H]^+^ Calcd for C_8_H_5_Cl_3_N^+^ 219.9482; Found 219.9485.

**6-Bromo-2,3-dichloro-1*H*-indole (7c):** Yield: 49%, 64.0 mg, white solid, mp 183–185 °C, purified using petroleum ether/ethyl acetate (10:1). ^1^H NMR (600 MHz, CDCl_3_) *δ* 8.15 (brs, 1H), 7.47 (d, *J* = 1.4 Hz, 1H), 7.42 (d, *J* = 8.5 Hz, 1H), 7.33 (dd, *J* = 8.5, 1.5 Hz, 1H). ^13^C{^1^H} NMR (151 MHz, CDCl_3_) *δ* 133.7, 124.6, 124.5, 120.7, 119.2, 117.0, 113.8, 104.2. HRMS (ESI) *m*/*z*: [M+H]^+^ Calcd for C_8_H_5_BrCl_2_N^+^ 263.8977; Found 263.8978.

### 4.3. Control Experiments

#### 4.3.1. The Effect of Radical Scavenger TEMPO on the Reaction

To an oven-dried 25 mL Schlenk tube equipped with a magnetic stir bar, **1a** (0.5 mmol, 1.0 equiv.), *^t^*BuOCl (1.25 mmol, 2.5 equiv.), TEMPO (1.5 mmol, 3.0 equiv.), and EtOAc (3.0 mL) were added and sealed under an air atmosphere. The reaction mixture was stirred at 40 °C for 16 h, and then cooled to room temperature. The solvent was removed under reduced pressure and the crude product was purified by silica gel column chromatography to afford the chlorination product **8** (84.9 mg, 76% yield).

**ethyl 3-chloro-1*H*-indole-2-carboxylate (8):** Yield: 76%, 84.9 mg, white solid, mp 138–140 °C, purified using petroleum ether/ethyl acetate (10:1). ^1^H NMR (500 MHz, CDCl_3_) *δ* 9.35 (s, 1H), 7.75 (d, *J* = 8.1 Hz, 1H), 7.45–7.36 (m, 2H), 7.26–7.23 (m, 1H), 4.51 (q, *J* = 7.1 Hz, 2H), 1.49 (t, *J* = 7.1 Hz, 3H). ^13^C{^1^H} NMR (126 MHz, CDCl_3_) δ 161.3, 134.9, 126.6, 126.2, 122.4, 121.3, 120.2, 112.4, 112.2, 61.5, 14.4.

#### 4.3.2. The Effect of Radical Scavenger BHT and DPE on the Reaction

To an oven-dried 25 mL Schlenk tube equipped with a magnetic stir bar, **1a** (0.5 mmol, 1.0 equiv.), *^t^*BuOCl (1.25 mmol, 2.5 equiv.), BHT or DPE (1.5 mmol, 3.0 equiv.), and EtOAc (3.0 mL) were added and sealed under an air atmosphere. The reaction mixture was stirred at 40 °C for 16 h, and then cooled to room temperature. The corresponding reaction mixture was analyzed by TLC.

#### 4.3.3. The Effect of Water and Air on the Reaction

**Method A**: To an oven-dried 25 mL Schlenk tube equipped with a magnetic stir bar, **1a** (0.5 mmol, 1.0 equiv.), *^t^*BuOCl (1.25 mmol, 2.5 equiv.), and CH_3_CN (3.0 mL) were added and sealed under an air atmosphere. The reaction mixture was stirred at 40 °C for 16 h, and then cooled to room temperature. The solvent was removed under reduced pressure and the crude product was purified by silica gel column chromatography to afford the desired product **2a** (104.2 mg, 87% yield).

**Method B**: To an oven-dried 25 mL Schlenk tube equipped with a magnetic stir bar, **1a** (0.5 mmol, 1.0 equiv.), *^t^*BuOCl (1.25 mmol, 2.5 equiv.), and CH_3_CN (dry, 3.0 mL) were added under an N_2_ atmosphere. The reaction mixture was stirred at 40 °C for 16 h, and then cooled to room temperature. The solvent was removed under reduced pressure and the crude product was purified by silica gel column chromatography to afford the desired product **2a** (106.6 mg, 89% yield).

**Method C**: To an oven-dried 25 mL Schlenk tube equipped with a magnetic stir bar, **1a** (0.5 mmol, 1.0 equiv.), *^t^*BuOCl (1.25 mmol, 2.5 equiv.), H_2_O (2.0 mmol, 4.0 equiv.), and CH_3_CN (dry, 3.0 mL) were added under an N_2_ atmosphere. The reaction mixture was stirred at 40 °C for 16 h, and then cooled to room temperature. The solvent was removed under reduced pressure and the crude product was purified by silica gel column chromatography to afford the desired product **2a** (100.6 mg, 84% yield).

## Figures and Tables

**Table 1:** Optimization of reaction conditions ^a^.

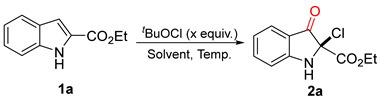				
Entry	*^t^*BuOCl (x Equiv.)	Solvent	Temp. (°C)	Yield (%) ^b^
1	3.0	THF	60	77
2	3.0	EtOAc	60	91
3	3.0	1,4-dioxane	60	61
4	3.0	CH_3_CN	60	87
5	3.0	toluene	60	53
6	2.5	EtOAc	60	94
7	2.0	EtOAc	60	85
8	1.5	EtOAc	60	56
9	2.5	EtOAc	50	97
10	2.5	EtOAc	40	99
11	2.5	EtOAc	30	92
12 ^c^	2.5	EtOAc	40	99
13 ^d^	2.5	EtOAc	40	88

^a^ Reaction conditions: **1a** (0.5 mmol) and *^t^*BuOCl (x equiv.) in solvent (3.0 mL) at heating for 20 h. ^b^ Isolated yield. ^c^ The reaction was performed for 16 h. ^d^ The reaction was performed for 12 h.

## Data Availability

Data obtained in this project are contained within this article and are available upon request from the authors.

## Supplementary Materials

The following supporting information can be downloaded at https://www.mdpi.com/article/10.3390/molecules30010102/s1. Crystallographic data and copies of NMR spectra [43,50].
