# Supplementary material for: tert-Butyl Hypochlorite: A Reagent for the Synthesis of Chlorinated Oxindole and Indole Derivatives
Source: Molecules. 2024 Dec 30;30(1):102. doi: 10.3390/molecules30010102 (PMC11721607; doi:10.3390/molecules30010102)

Supporting Information

tert-Butyl Hypochlorite: A Reagent for the Synthesis of Chlorinated Oxindole and Indole Derivatives

Table of Contents

1. General Information……………………………………………………………………….............S3

2.The Typical Procedure for Chlorooxidation of Indoles with ^{t}BuOCl……..............................S3-S7

3. Screening of Conditions for Chlorination of 2-Oxindoles with ^{t}BuOCl…………………………...S7

4.The Typical Procedure for Chlorination of 2-Oxindoles with ^{t}BuOCl……… ……………S8-S9

5.The Typical Procedure for Decarboxylative Chlorination of Indole-2-carboxylic Acids …S9-S10

6.Control Experiments…………………………………………………….........................S10-S11

7. References…………………………………………………...S12

8.Copy of NMR Spectra……………………………………………… ………… …......S13-S40

1. General Information.

Unless otherwise noted, all reactions were carried out in oven-dried 25-mL Schlenk tubes under an air atmosphere. IKA plate was used as the heat source. All reagents and solvents were of pure analytical grade. Thin layer chromatography (TLC) was performed on HSGF254 silica gel, pre-coated on glass-backed plates coated with 0.2 mm silica and revealed with either a UV lamp (λ_{max} = 254 nm). The products were purified by flash column chromatography on silica gel 200-300 mesh. ^{1}H, ^{13}C and ^{19}F NMR spectra were recorded on a Bruker Avance NEO 600M NMR Spectrometer (600 MHz for ^{1}H, 151 MHz for ^{13}C, 565 MHz for ^{19}F), a Bruker Avance III HD 500M NMR Spectrometer (500 MHz for ^{1}H, 126 MHz for ^{13}C, 471 MHz for ^{19}F), using $CDCl_3$ or d_6-DMSO as the solvent with tetramethylsilane (TMS) as the internal standard at room temperature. The chemical shifts are reported in ppm downfield (δ) from TMS, the coupling constants J are given in Hz. The peak patterns are indicated as follows: s, singlet; d, doublet; t, triplet; q, quartet; m, multiplet. High resolution mass spectra were recorded on either a Q-TOF mass spectrometry or a LTQ Orbitrap XL mass spectrometry. Unless otherwise noted, starting materials are commercially available. Unless otherwise noted, starting materials are commercially available.

2. The Typical Procedure for Chlorooxidation of Indoles with ^{t}BuOCl.

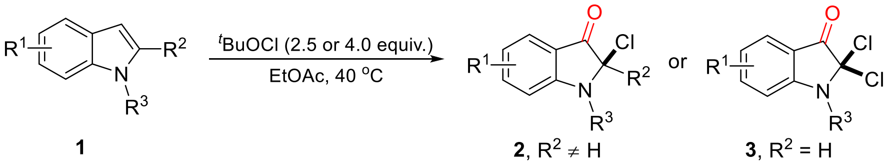

To an oven-dried 25 mL Schlenk tube equipped with a magnetic stir bar was added indole substrate **1** (0.5 mmol, 1.0 equiv.), ^{t}BuOCl (1.25 mmol, 2.5 equiv.) or ^{t}BuOCl (2.0 mmol, 4.0 equiv.), EtOAc (3.0 mL), and sealed under an air atmosphere. The reaction mixture was stirred at 40 °C for 16 h, and then cooled to room temperature. The solvent was removed under reduced pressure and the crude product was purified by silica gel column chromatography to afford the desired product **2** or **3**.

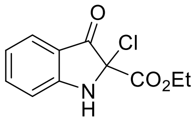

ethyl 2-chloro-3-oxoindoline-2-carboxylate (2a): Yield: 99%, 118.6 mg, white solid, mp 121–123 °C, purified using petroleum ether/ethyl acetate (5:1). ^{1}H NMR (600 MHz, $CDCl_3$) δ 9.28 (s, 1H), 7.45 (d, J = 7.6 Hz, 1H), 7.38–7.35 (m, 1H), 7.15–7.12 (m, 1H), 7.03 (d, J = 7.8 Hz, 1H), 4.35–4.23 (m, 2H), 1.26 (t, J = 7.1 Hz, 3H). $^{13}C\{^{1}H\}$ NMR (151 MHz, $CDCl_3$) δ 172.4, 165.0, 140.9, 131.4, 127.0, 125.0, 123.8, 111.2, 64.7, 63.8, 13.9. HRMS (ESI) m/z: $[M+H]^+$ Calcd for $C_{11}H_{11}ClNO_3^+$ 240.0422; Found 240.0425.

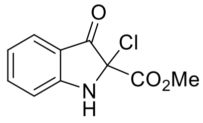

methyl 2-chloro-3-oxoindoline-2-carboxylate (2b): Yield: 91%, 102.6 mg, white solid, mp 117–119 °C, purified using petroleum ether/ethyl acetate (5:1). ^{1}H NMR (600 MHz, $CDCl_3$) δ 9.31 (brs, 1H), 7.45 (d, J = 7.6 Hz, 1H), 7.39–7.36 (m, 1H), 7.15–7.13 (m, 1H), 7.03 (d, J = 7.8 Hz, 1H), 3.84 (s, 3H). ^{13}C{^{1}H} NMR (151 MHz, $CDCl_3$) δ 172.3, 165.6, 140.9, 131.5, 126.8, 125.1, 123.9, 111.3, 64.6, 54.4. HRMS (ESI) m/z: [M+H]$^+$ Calcd for $C_{10}H_9ClNO_3^+$ 226.0265; Found 226.0267.

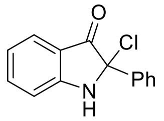

2-chloro-2-phenylindolin-3-one (2c): Yield: >99%, 121.0 mg, yellow solid, mp 130–132 °C, purified using petroleum ether/ethyl acetate (10:1~5:1). ^{1}H NMR (600 MHz, $CDCl_3$) δ 8.53–8.45 (m, 2H), 7.76 (d, J = 7.4 Hz, 1H), 7.65 (d, J = 7.7 Hz, 1H), 7.62–7.56 (m, 3H), 7.51–7.49 (m, 1H), 7.43–7.38 (m, 1H). ^{13}C{^{1}H} NMR (151 MHz, $CDCl_3$) δ 171.9, 149.3, 139.8, 132.1, 131.6, 129.5, 128.7, 128.6, 127.9, 122.7, 121.6, 80.2. HRMS (ESI) m/z: [M+H]$^+$ Calcd for $C_{14}H_{11}ClNO^+$ 244.0524; Found 244.0526.

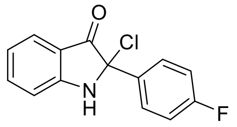

2-chloro-2-(4-fluorophenyl)indolin-3-one (2d): Yield: >99%, 130.5 mg, yellow solid, mp 123–125 °C, purified using petroleum ether/ethyl acetate (10:1~5:1). ^{1}H NMR (600 MHz, $CDCl_3$) δ 8.50–8.47 (m, 2H), 7.75 (d, J = 7.3 Hz, 1H), 7.63 (d, J = 7.7 Hz, 1H), 7.51–7.48 (m, 1H), 7.43–7.37 (m, 1H), 7.28–7.21 (m, 2H). ^{13}C{^{1}H} NMR (151 MHz, $CDCl_3$) δ 170.8, 165.1 (d, $J_{C\text{-}F}$ = 254.9 Hz), 149.2, 139.7, 131.8 (d, $J_{C\text{-}F}$ = 8.7 Hz), 131.6, 127.9, 124.9 (d, $J_{C\text{-}F}$ = 3.2 Hz), 122.8, 121.5, 115.9 (d, $J_{C\text{-}F}$ = 22.1 Hz), 80.1. ^{19}F NMR (565 MHz, $CDCl_3$) δ -106.2 (s, 1F). HRMS (ESI) m/z: [M+H]$^+$ Calcd for $C_{14}H_{10}ClFNO^+$ 262.0429; Found 262.0431.

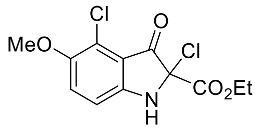

ethyl 2,4-dichloro-5-methoxy-3-oxoindoline-2-carboxylate (2e): Yield: 93%, 142.1 mg, white solid, mp 130–132 °C, purified using petroleum ether/ethyl acetate (5:1). ^{1}H NMR (600 MHz, $CDCl_3$) δ 9.17 (1H), 6.95–6.91 (m, 2H), 4.38–4.28 (m, 2H), 3.91 (s, 3H), 1.27 (t, J = 7.1 Hz, 3H). ^{13}C{^{1}H} NMR (151 MHz, $CDCl_3$) δ 171.3, 163.4, 152.0, 134.9, 126.9, 121.2, 113.9, 109.6, 65.8, 64.1, 56.8, 14.0. HRMS (ESI) m/z: [M+H]$^+$ Calcd for $C_{12}H_{12}Cl_2NO_4^+$ 304.0138; Found 304.0141.

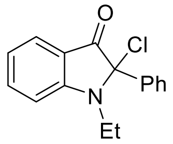

2-chloro-1-ethyl-2-phenylindolin-3-one (2f): Yield: 65%, 88.6 mg, white solid, mp 90–92 °C, purified using petroleum ether. ^{1}H NMR (500 MHz, $CDCl_3$) δ 7.65–7.53 (m, 5H), 7.53–7.44 (m, 3H), 7.27 (d, J = 1.9 Hz, 1H), 4.45 (q, J = 7.0 Hz, 2H), 1.20 (t, J = 7.1 Hz, 3H). $^{13}C\{^1H\}$ NMR (126 MHz, $CDCl_3$) δ 139.4, 130.6, 129.6, 129.4, 129.31, 129.26, 128.7, 126.0, 124.5, 117.5, 116.8, 104.6, 41.0, 17.1. HRMS (ESI) *m*/*z*: $[M+H]^+$ Calcd for $C_{16}H_{15}ClNO^+$ 272.0837; Found 272.0841.

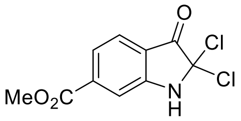

methyl 2,2-dichloro-3-oxoindoline-6-carboxylate (3g): Yield: 50%, 65.6 mg, white solid, mp 178–180 °C, purified using petroleum ether/ethyl acetate (2:1). ^{1}H NMR (500 MHz, d_6-DMSO) δ 11.56 (s, 1H), 7.90–7.68 (m, 2H), 7.44 (s, 1H), 3.87 (s, 3H). $^{13}C\{^1H\}$ NMR (126 MHz, d_6-DMSO) δ 169.2, 165.6, 140.0, 133.5, 125.6, 125.2, 111.8, 74.6, 53.1. HRMS (ESI) *m*/*z*: $[M+H]^+$ Calcd for $C_{10}H_8Cl_2NO_3^+$ 259.9876; Found 259.9878.

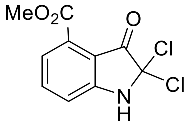

methyl 2,2-dichloro-3-oxoindoline-4-carboxylate (3h): Yield: 64%, 82.7 mg, light yellow solid, mp 136–138 °C, purified using petroleum ether/ethyl acetate (2:1). ^{1}H NMR (500 MHz, d_6-DMSO) δ 11.55 (s, 1H), 7.89–7.58 (m, 2H), 7.25–7.23 (m, 1H), 3.92 (s, 3H). $^{13}C\{^1H\}$ NMR (126 MHz, d_6-DMSO) δ 169.6, 164.6, 141.2, 133.2, 128.0, 127.6, 125.3, 116.1, 75.3, 52.8. HRMS (ESI) *m*/*z*: $[M+H]^+$ Calcd for $C_{10}H_8Cl_2NO_3^+$ 259.9876; Found 259.9877.

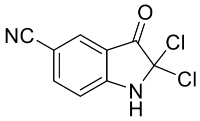

2,2-dichloro-3-oxoindoline-5-carbonitrile (3i): Yield: 76%, 86.7 mg, brown solid, mp 212–214 °C, purified using petroleum ether/ethyl acetate (2:1). ^{1}H NMR (500 MHz, d_6-DMSO) δ 11.86 (s, 1H), 8.24 (s, 1H), 7.86 (d, J = 4.9 Hz, 1H), 7.13 (d, J = 5.5 Hz, 1H). $^{13}C\{^1H\}$ NMR (126 MHz, d_6-DMSO) δ 169.4, 143.8, 137.7, 130.2, 129.3, 118.6, 112.8, 106.4, 73.9. HRMS (ESI) *m*/*z*: $[M+H]^+$ Calcd for $C_9H_5Cl_2N_2O^+$ 226.9773; Found 226.9772.

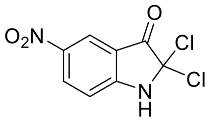

2,2-dichloro-5-nitroindolin-3-one (3j): Yield: 68%, 84.2 mg, yellow solid, mp 154–156 °C, purified using petroleum ether/ethyl acetate (2:1). ^{1}H NMR (500 MHz, d_6-DMSO) δ 12.04 (s, 1H), 8.43 (s, 1H), 8.30 (s, 1H), 7.17 (s, 1H). $^{13}C\{^1H\}$ NMR (126 MHz, d_6-DMSO) δ 169.7, 145.6, 143.8, 129.8, 129.3, 120.9, 112.5, 73.8. HRMS (ESI) *m*/*z*: $[M+H]^+$ Calcd for $C_8H_5Cl_2N_2O_3^+$ 246.9672; Found 246.9675.

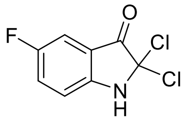

2,2-dichloro-5-fluoroindolin-3-one (3k): Yield: 73%, 80.3 mg, white solid, mp 156–158 °C, purified using petroleum ether/ethyl acetate (10:1~5:1). ^{1}H NMR (500 MHz, $CDCl_3$) δ 9.54 (s, 1H), 7.37 (dd, J = 7.2, 2.5 Hz, 1H), 7.14-7.10 (m, 1H), 7.03 (dd, J = 8.6, 4.1 Hz, 1H). ^{13}C{^{1}H} NMR (126 MHz, $CDCl_3$) δ 171.45, 159.7 (d, $J_{C\text{-}F}$ = 244.9 Hz), 133.9 (d, $J_{C\text{-}F}$ = 2.3 Hz), 130.8 (d, $J_{C\text{-}F}$ = 8.6 Hz), 118.9 (d, $J_{C\text{-}F}$ = 23.8 Hz), 112.8 (d, $J_{C\text{-}F}$ = 29.1 Hz), 112.7 (d, $J_{C\text{-}F}$ = 4.6 Hz), 74.3. ^{19}F NMR (471 MHz, $CDCl_3$) δ -116.8 (s, 1F). HRMS (ESI) m/z: $[M+H]^+$ Calcd for $C_8H_5Cl_2FNO^+$ 219.9727; Found 219.9730.

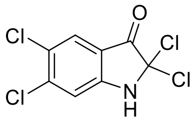

2,2,5,6-tetrachloroindolin-3-one (3l): Yield: 73%, 98.5 mg, yellow solid, mp 200–202 °C, purified using petroleum ether/ethyl acetate (10:1~5:1). ^{1}H NMR (500 MHz, d_6-DMSO) δ 11.64 (s, 1H), 8.04 (s, 1H), 7.20 (s, 1H). ^{13}C{^{1}H} NMR (126 MHz, d_6-DMSO) δ 169.2, 139.6, 135.3, 129.5, 127.3, 126.2, 113.7, 74.1. HRMS (ESI) m/z: $[M+H]^+$ Calcd for $C_8H_4Cl_4NO^+$ 269.9042; Found 269.9041.

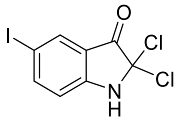

2,2-dichloro-5-iodoindolin-3-one (3m): Yield: 70%, 114.9 mg, yellow solid, mp 230–232 °C, purified using petroleum ether/ethyl acetate (10:1~5:1). ^{1}H NMR (500 MHz, d_6-DMSO) δ 11.46 (s, 1H), 7.97 (s, 1H), 7.75 (d, J = 7.9 Hz, 1H), 6.82 (d, J = 8.1 Hz, 1H). ^{13}C{^{1}H} NMR (126 MHz, d_6-DMSO) δ 169.0, 141.4, 139.3, 133.4, 131.4, 114.1, 86.6, 74.6. HRMS (ESI) m/z: $[M+H]^+$ Calcd for $C_8H_5Cl_2INO^+$ 327.8787; Found 327.8789.

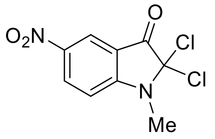

2,2-dichloro-1-methyl-5-nitroindolin-3-one (3n): Yield: 64%, 83.0 mg, yellow solid, mp 184–186 °C, purified using petroleum ether/ethyl acetate (5:1). ^{1}H NMR (500 MHz, $CDCl_3$) δ 8.50 (s, 1H), 8.40–8.35 (m, 1H), 7.06 (d, J = 8.7 Hz, 1H), 3.38 (s, 3H). ^{13}C{^{1}H} NMR (126 MHz, $CDCl_3$) δ 168.7, 146.0, 144.4, 129.9, 128.4, 120.9, 109.4, 72.4, 27.6. HRMS (ESI) m/z: $[M+H]^+$ Calcd for $C_9H_7Cl_2N_2O_3^+$ 260.9828; Found 260.9827.

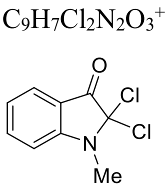

2,2-dichloro-1-methylindolin-3-one (3o): Yield: 56%, 60.3 mg, white solid, mp 76–78 °C, purified using petroleum ether/ethyl acetate (10:1). ^{1}H NMR (600 MHz, $CDCl_3$) δ 7.66 (d, J = 7.2 Hz, 1H),

7.45–7.42 (m, 1H), 7.23–7.20 (m, 1H), 6.89 (d, *J* = 7.9 Hz, 1H), 3.30 (s, 3H). $^{13}C\{^1H\}$ NMR (151 MHz, $CDCl_3$) *δ* 169.0, 140.7, 131.9, 129.3, 124.8, 124.3, 109.2, 74.3, 27.1. HRMS (ESI) *m*/*z*: $[M+H]^+$ Calcd for $C_9H_8Cl_2NO^+$ 215.9977; Found 215.9978.

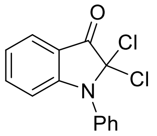

2,2-dichloro-1-phenylindolin-3-one (3p): Yield: 46%, 64.2 mg, white solid, mp 86–88 °C, purified using petroleum ether/ethyl acetate (10:1). ^1H NMR (600 MHz, $CDCl_3$) *δ* 7.74 (d, *J* = 7.6 Hz, 1H), 7.60–7.57 (m, 2H), 7.5 –7.45 (m, 3H), 7.38–7.35 (m, 1H), 7.29–7.24 (m, 1H), 6.85 (d, *J* = 8.0 Hz, 1H). $^{13}C\{^1H\}$ NMR (151 MHz, $CDCl_3$) *δ* 168.1, 140.8, 133.0, 131.8, 129.8, 129.0, 128.9, 126.3, 125.1, 124.6, 110.3, 74.5. HRMS (ESI) *m*/*z*: $[M+H]^+$ Calcd for $C_{14}H_{10}Cl_2NO^+$ 278.0134; Found 278.0136.

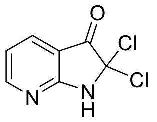

2,2-dichloro-1,2-dihydro-3*H*-pyrrolo[2,3-*b*]pyridin-3-one (3q): Yield: 20%, 20.8 mg, white solid, mp 160–162 °C, purified using petroleum ether/ethyl acetate (2:1). ^1H NMR (500 MHz, $CDCl_3$) *δ* 9.65 (s, 1H), 8.35 (d, *J* = 4.2 Hz, 1H), 7.95 (d, *J* = 7.3 Hz, 1H), 7.21–7.18 (m, 1H). $^{13}C\{^1H\}$ NMR (126 MHz, $CDCl_3$) *δ* 168.8, 153.3, 149.5, 133.8, 125.0, 119.8, 73.1. HRMS (ESI) *m*/*z*: $[M+H]^+$ Calcd for $C_7H_5Cl_2N_2O^+$ 202.9773; Found 202.9776.

3. Screening of Conditions for Chlorination of 2-Oxindoles with ^{t}BuOCl.

Table S1. Optimization study[a]

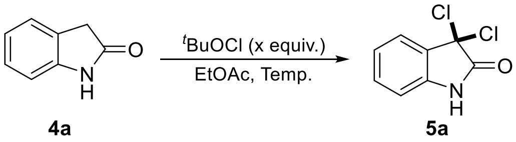

Entry	^{t}BuOCl (x equiv.)	T (°C)	Reaction time (h)	Yield (%)[b]
1	2.5	40	16	30
2	3.0	40	16	38
3	3.5	40	16	50
4	4.0	40	16	57
5	4.5	40	16	56
6	4.0	50	16	69
7	4.0	60	16	78
8	4.0	70	16	76
9	4.0	60	20	85
10	4.0	60	24	86
11	4.0	60	28	86

[a]Reaction conditions: **1a** (0.5 mmol) and ^{t}BuOCl (x equiv.) in EtOAc (3.0 mL) at heating. [b]Isolated yield.

4. The Typical Procedure for Chlorination of 2-Oxindoles with ^{t}BuOCl.

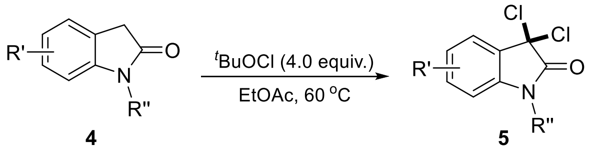

To an oven-dried 25 mL Schlenk tube equipped with a magnetic stir bar was added indole substrate **4** (0.5 mmol, 1.0 equiv.), ^{t}BuOCl (2.0 mmol, 4.0 equiv.), EtOAc (3.0 mL), and sealed under an air atmosphere. The reaction mixture was stirred at 60 °C for 24 h, and then cooled to room temperature. The solvent was removed under reduced pressure and the crude product was purified by silica gel column chromatography to afford the desired product **5**.

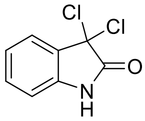

3,3-dichloroindolin-2-one (5a)[1]: Yield: 86%, 87.3 mg, white solid, mp 176–178 °C, purified using petroleum ether/ethyl acetate (5:1). ^{1}H NMR (500 MHz, d_6-DMSO) δ 11.47 (s, 1H), 7.74 (s, 1H), 7.44 (d, J = 8.5 Hz, 1H), 6.98 (d, J = 8.6 Hz, 1H). ^{13}C{^{1}H} NMR (126 MHz, d_6-DMSO) δ 169.3, 138.5, 132.8, 130.9, 128.0, 125.3, 113.4, 74.8. HRMS (ESI) *m*/*z*: [M+H]$^+$ Calcd for $C_8H_6Cl_2NO^+$ 201.9821; Found 201.9822.

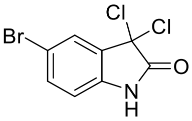

5-bromo-3,3-dichloroindolin-2-one (5b): Yield: >99%, 140.1 mg, white solid, mp 198–200 °C, purified using petroleum ether/ethyl acetate (5:1). ^{1}H NMR (500 MHz, d_6-DMSO) δ 11.49 (s, 1H), 7.87 (s, 1H), 7.59 (d, J = 8.3 Hz, 1H), 6.94 (d, J = 8.4 Hz, 1H). ^{13}C{^{1}H} NMR (126 MHz, d_6-DMSO) δ 169.2, 138.9, 135.6, 131.2, 128.0, 115.4, 113.9, 74.7. HRMS (ESI) *m*/*z*: [M+H]$^+$ Calcd for $C_8H_5BrCl_2NO^+$ 279.8926; Found 279.8925.

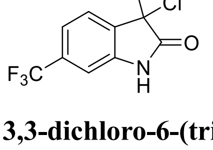

3,3-dichloro-6-(trifluoromethyl)indolin-2-one (5c): Yield: 97%, 131.4 mg, white solid, mp 156–158 °C, purified using petroleum ether/ethyl acetate (5:1). ^{1}H NMR (500 MHz, d_6-DMSO) δ 11.73 (s, 1H), 7.91 (d, J = 7.9 Hz, 1H), 7.53 (d, J = 7.9 Hz, 1H), 7.23 (s, 1H). ^{13}C{^{1}H} NMR (126 MHz, d_6-DMSO) δ 169.2, 140.6, 133.1, 132.7 (q, $J_{C\text{-}F}$ = 30.2 Hz), 126.3, 123.8 (q, $J_{C\text{-}F}$ = 273.4 Hz), 121.0 (q, $J_{C\text{-}F}$ = 3.9 Hz), 108.4 (q, $J_{C\text{-}F}$ = 4.0 Hz), 74.2. ^{19}F NMR (471 MHz, d_6-DMSO) δ -61.8 (s, 3F). HRMS (ESI) *m*/*z*: [M+H]$^+$ Calcd for $C_9H_5Cl_2F_3NO^+$ 269.9695; Found 269.9693.

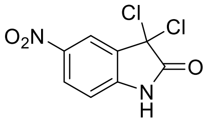

3,3-dichloro-5-nitroindolin-2-one (5d)[2]: Yield: 70%, 86.5 mg, yellow solid, mp 172–174 °C, purified using petroleum ether/ethyl acetate (5:1). ^{1}H NMR (500 MHz, d_6-DMSO) δ 12.04 (s, 1H), 8.45 (s, 1H), 8.32 (d, J = 8.7 Hz, 1H), 7.18 (d, J = 8.7 Hz, 1H). ^{13}C{^{1}H} NMR (126 MHz, d_6-DMSO) δ 169.7, 145.6, 143.8, 129.8, 129.4, 121.0, 112.5, 73.8. HRMS (ESI) m/z: $[M+H]^+$ Calcd for $C_8H_5Cl_2N_2O_3^+$ 246.9672; Found 246.9673.

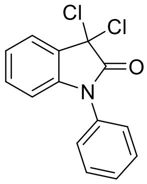

3,3-dichloro-1-phenylindolin-2-one (5e)[1]: Yield: 49%, 67.5 mg, yellow oil, purified using petroleum ether/ethyl acetate (10:1). ^{1}H NMR (500 MHz, $CDCl_3$) δ 7.78–7.69 (m, 1H), 7.60–7.57 (m, 2H), 7.52–7.44 (m, 3H), 7.39–7.31 (m, 1H), 7.26–7.23 (m, 1H), 6.82 (dd, J = 26.8, 8.2 Hz, 1H). ^{13}C{^{1}H} NMR (126 MHz, $CDCl_3$) δ 168.1, 131.8, 130.1, 129.9, 129.2, 129.0, 126.4, 126.3, 125.5, 125.2, 124.7, 111.6, 110.4, 74.6. HRMS (ESI) m/z: $[M+H]^+$ Calcd for $C_{14}H_{10}Cl_2NO^+$ 278.0134; Found 278.0135.

5. The Typical Procedure for Decarboxylative Chlorination of Indole-2-carboxylic Acids.

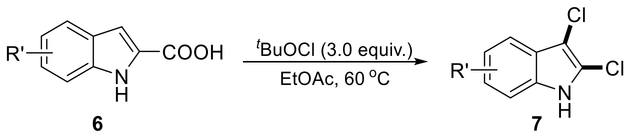

To an oven-dried 25 mL Schlenk tube equipped with a magnetic stir bar was added indole-2-carboxylic acid substrate **6** (0.5 mmol, 1.0 equiv.), ^{t}BuOCl (1.5 mmol, 3.0 equiv.), EtOAc (3.0 mL), and sealed under an air atmosphere. The reaction mixture was stirred at 60 °C for 24 h, and then cooled to room temperature. The solvent was removed under reduced pressure and the crude product was purified by silica gel column chromatography to afford the desired product **7**.

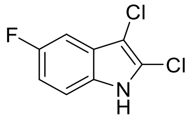

2,3-dichloro-5-fluoro-1*H*-indole (7a): Yield: 52%, 52.5 mg, white solid, mp 131–133 °C, purified using petroleum ether/ethyl acetate (10:1). ^{1}H NMR (600 MHz, $CDCl_3$) δ 8.14 (brs, 1H), 7.27–7.18 (m, 2H), 7.03–7.00 (m, 1H). ^{13}C{^{1}H} NMR (151 MHz, $CDCl_3$) δ 158.5 (d, $J_{C\text{-}F}$ = 238.1 Hz), 129.6, 126.1 (d, $J_{C\text{-}F}$ = 10.7 Hz), 121.7, 112.0 (d, $J_{C\text{-}F}$ = 20.9 Hz), 111.9 (d, $J_{C\text{-}F}$ = 3.7 Hz), 103.9 (d, $J_{C\text{-}F}$ =4.5 Hz), 103.3 (d, $J_{C\text{-}F}$ = 25.8 Hz). ^{19}F NMR (565 MHz, $CDCl_3$) δ -121.7. HRMS (ESI) m/z: $[M+H]^+$ Calcd for $C_8H_5Cl_2FN^+$ 203.9778; Found 203.9780.

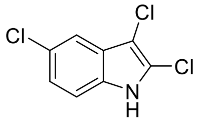

2,3,5-trichloro-1*H*-indole (7b): Yield: 43%, 47.0 mg, white solid, mp 148–150 °C, purified using petroleum ether/ethyl acetate (10:1). ^{1}H NMR (600 MHz, $CDCl_3$) δ 8.18 (brs, 1H), 7.54 (s, 1H), 7.24–7.19 (m, 2H). ^{13}C{^{1}H} NMR (151 MHz, $CDCl_3$) δ 131.5, 127.1, 126.5, 123.9, 121.5, 117.5, 112.0, 103.5. HRMS (ESI) *m*/*z*: $[M+H]^+$ Calcd for $C_8H_5Cl_3N^+$ 219.9482; Found 219.9485.

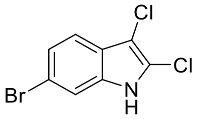

6-Bromo-2,3-dichloro-1*H*-indole (7c): Yield: 49%, 64.0 mg, white solid, mp 183–185 °C, purified using petroleum ether/ethyl acetate (10:1). ^{1}H NMR (600 MHz, $CDCl_3$) δ 8.15 (brs, 1H), 7.47 (d, J = 1.4 Hz, 1H), 7.42 (d, J = 8.5 Hz, 1H), 7.33 (dd, J = 8.5, 1.5 Hz, 1H). ^{13}C{^{1}H} NMR (151 MHz, $CDCl_3$) δ 133.7, 124.6, 124.5, 120.7, 119.2, 117.0, 113.8, 104.2. HRMS (ESI) *m*/*z*: $[M+H]^+$ Calcd for $C_8H_5BrCl_2N^+$ 263.8977; Found 263.8978.

6. Control Experiments.

(1) The effect of radical scavenger TEMPO on the reaction.

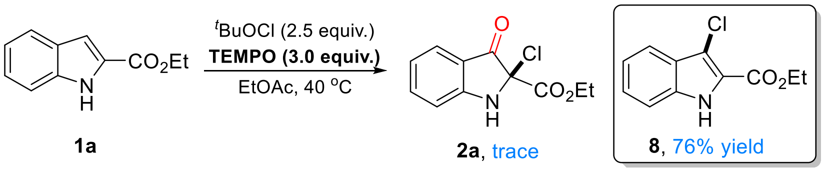

To an oven-dried 25 mL Schlenk tube equipped with a magnetic stir bar was added **1a** (0.5 mmol, 1.0 equiv.), ^{t}BuOCl (1.25 mmol, 2.5 equiv.), TEMPO (1.5 mmol, 3.0 equiv.), EtOAc (3.0 mL), and sealed under an air atmosphere. The reaction mixture was stirred at 40 °C for 16 h, and then cooled to room temperature. The solvent was removed under reduced pressure and the crude product was purified by silica gel column chromatography to afford the chlorination product **8** (84.9 mg, 76% yield).

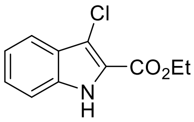

ethyl 3-chloro-1*H*-indole-2-carboxylate (8): Yield: 76%, 84.9 mg, white solid, mp 138–140 °C, purified using petroleum ether/ethyl acetate (10:1). ^{1}H NMR (500 MHz, $CDCl_3$) δ 9.35 (s, 1H), 7.75 (d, J = 8.1 Hz, 1H), 7.45–7.36 (m, 2H), 7.26–7.23 (m, 1H), 4.51 (q, J = 7.1 Hz, 2H), 1.49 (t, J = 7.1 Hz, 3H). ^{13}C{^{1}H} NMR (126 MHz, $CDCl_3$) δ 161.3, 134.9, 126.6, 126.2, 122.4, 121.3, 120.2, 112.4, 112.2, 61.5, 14.4.

(2) The effect of radical scavenger BHT and DPE on the reaction.

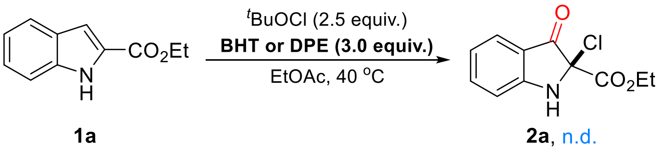

To an oven-dried 25 mL Schlenk tube equipped with a magnetic stir bar was added **1a** (0.5 mmol,

1.0 equiv.), ^{t}BuOCl (1.25 mmol, 2.5 equiv.), BHT or DPE (1.5 mmol, 3.0 equiv.), EtOAc (3.0 mL), and sealed under an air atmosphere. The reaction mixture was stirred at 40 °C for 16 h, and then cooled to room temperature. The corresponding reaction mixture was analyzed by TLC.

(3) The effect of water and air on the reaction.

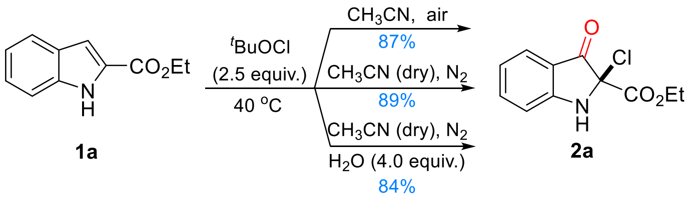

1a **2a**

Method A: To an oven-dried 25 mL Schlenk tube equipped with a magnetic stir bar was added **1a** (0.5 mmol, 1.0 equiv.), ^{t}BuOCl (1.25 mmol, 2.5 equiv.), CH_3CN (3.0 mL), and sealed under an air atmosphere. The reaction mixture was stirred at 40 °C for 16 h, and then cooled to room temperature. The solvent was removed under reduced pressure and the crude product was purified by silica gel column chromatography to afford the desired product **2a** (104.2 mg, 87% yield).

Method B: To an oven-dried 25 mL Schlenk tube equipped with a magnetic stir bar was added **1a** (0.5 mmol, 1.0 equiv.), ^{t}BuOCl (1.25 mmol, 2.5 equiv.) and CH_3CN (dry, 3.0 mL) under an N_2 atmosphere. The reaction mixture was stirred at 40 °C for 16 h, and then cooled to room temperature. The solvent was removed under reduced pressure and the crude product was purified by silica gel column chromatography to afford the desired product **2a** (106.6 mg, 89% yield).

Method C: To an oven-dried 25 mL Schlenk tube equipped with a magnetic stir bar was added **1a** (0.5 mmol, 1.0 equiv.), ^{t}BuOCl (1.25 mmol, 2.5 equiv.), H_2O (2.0 mmol, 4.0 equiv.) and CH_3CN (dry, 3.0 mL) under an N_2 atmosphere. The reaction mixture was stirred at 40 °C for 16 h, and then cooled to room temperature. The solvent was removed under reduced pressure and the crude product was purified by silica gel column chromatography to afford the desired product **2a** (100.6 mg, 84% yield).

7. References.

(1) Li, X.; Wang, B.; Li, D.; Zhao, J.; Qu, J.; Zhou, Y. KI-Mediated Chlorine Gas-Free Synthesis of 3,3 -Dichloro-2-oxindole Derivatives. *Eur. J. Org. Chem.* **2023**, *26*, e202201452.

(2) Ma, T.; Zheng, Y.; Huang, S. SO_2ClF: A Reagent for Controllable Chlorination and Chlorooxidation of Simple Unprotected Indoles. *J. Org. Chem.* **2023**, *88,* 4839−4847.

8. Copy of NMR Spectra.

^{1}H NMR (600 MHz) of 2a in $CDCl_3$

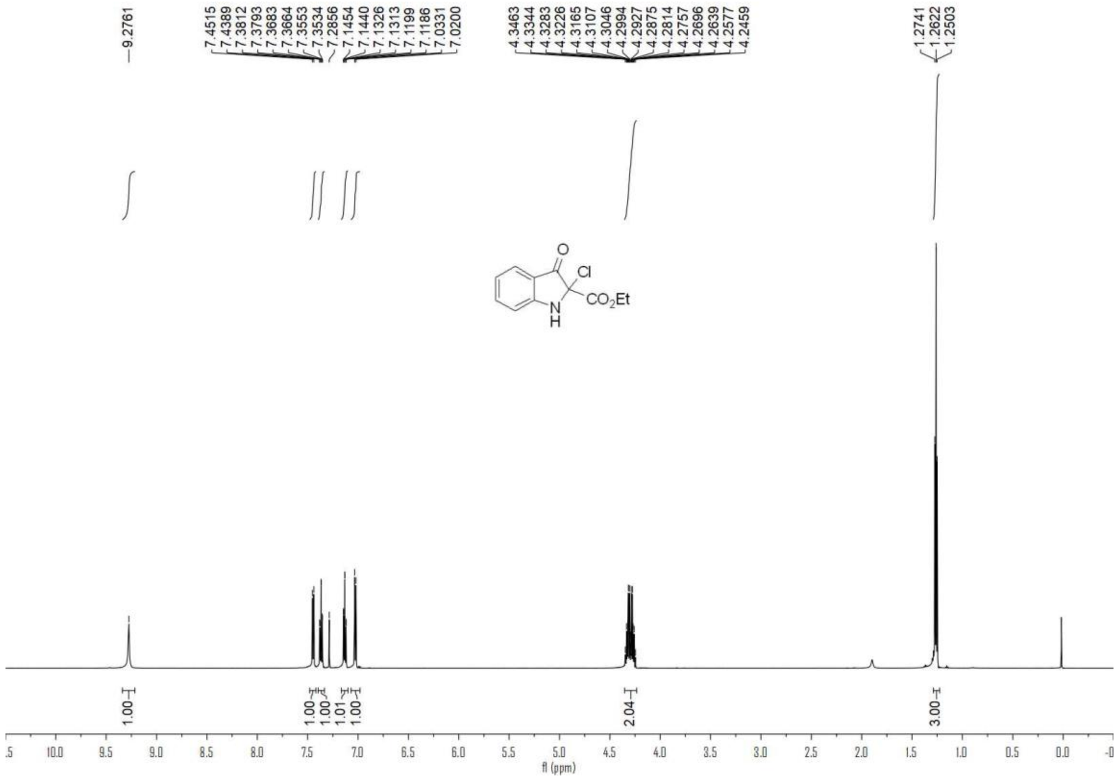

^{13}C{^{1}H} NMR (151 MHz) of 2a in $CDCl_3$

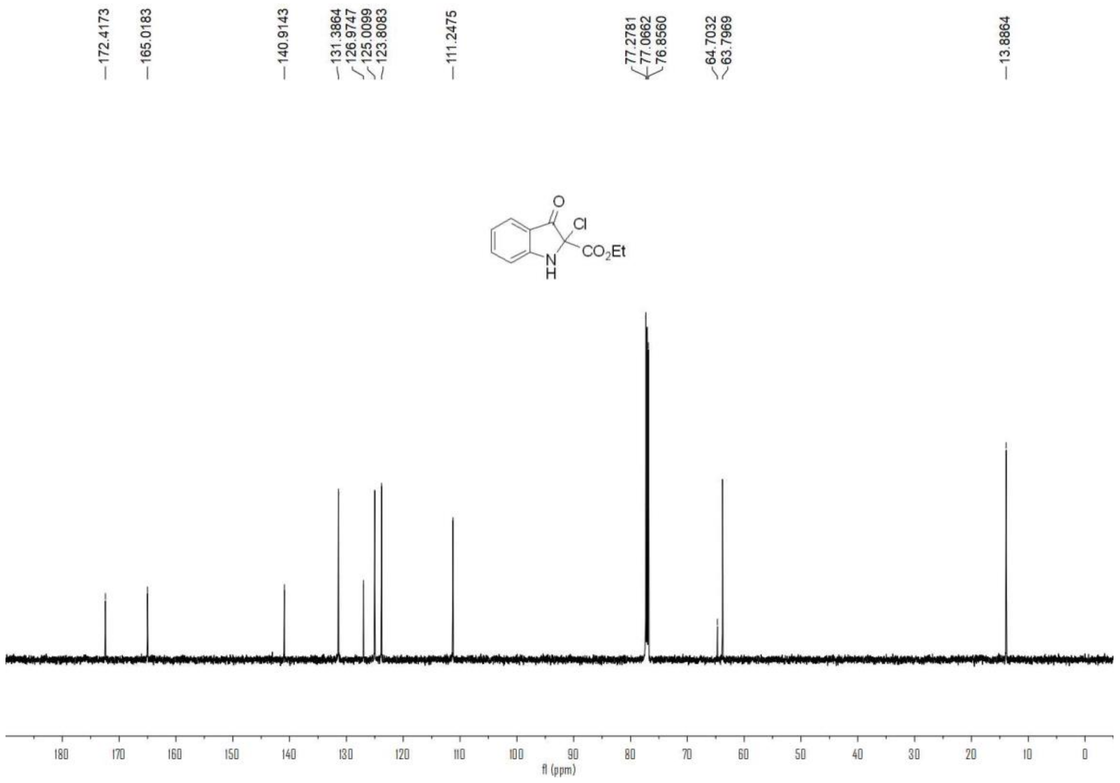

^{1}H NMR (600 MHz) of 2b in $CDCl_3$

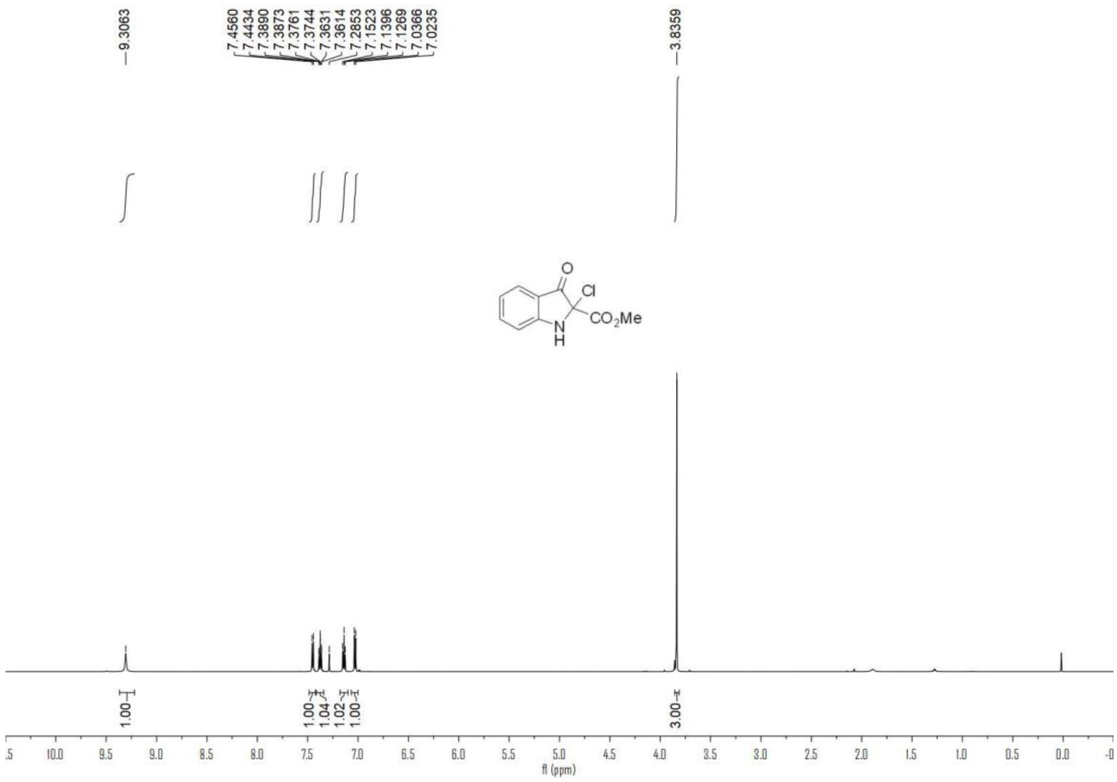

^{13}C{^{1}H} NMR (151 MHz) of 2b in $CDCl_3$

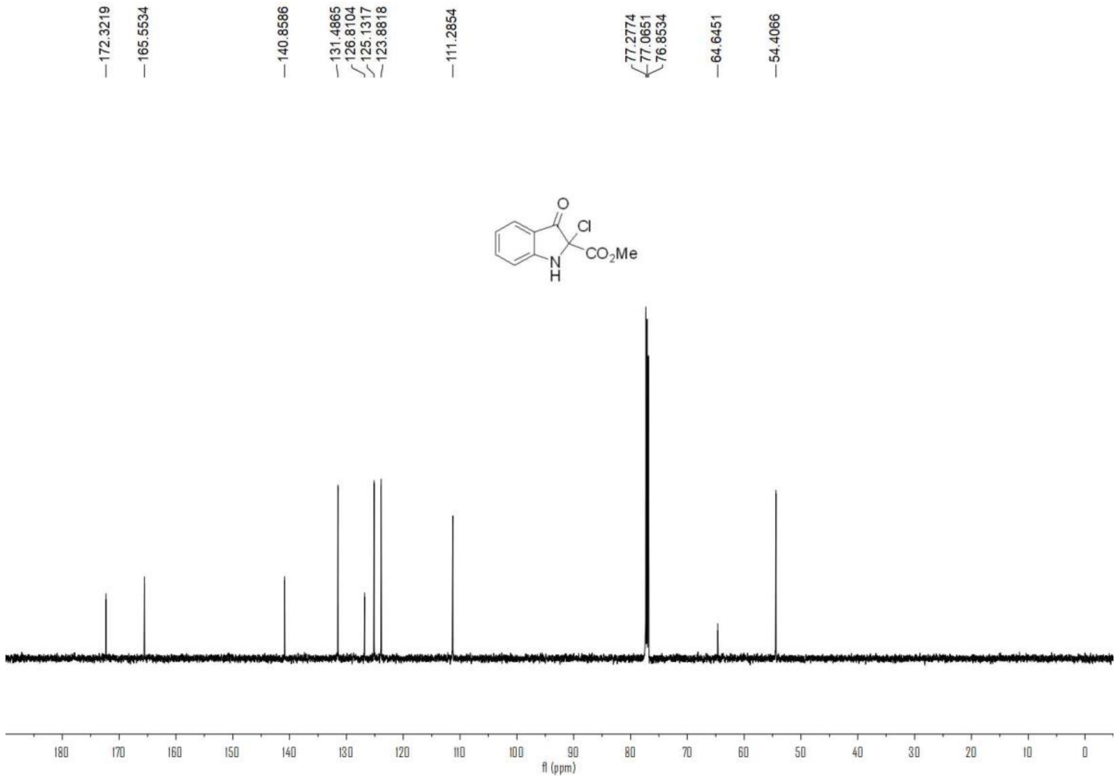

^{1}H NMR (600 MHz) of 2c in $CDCl_3$

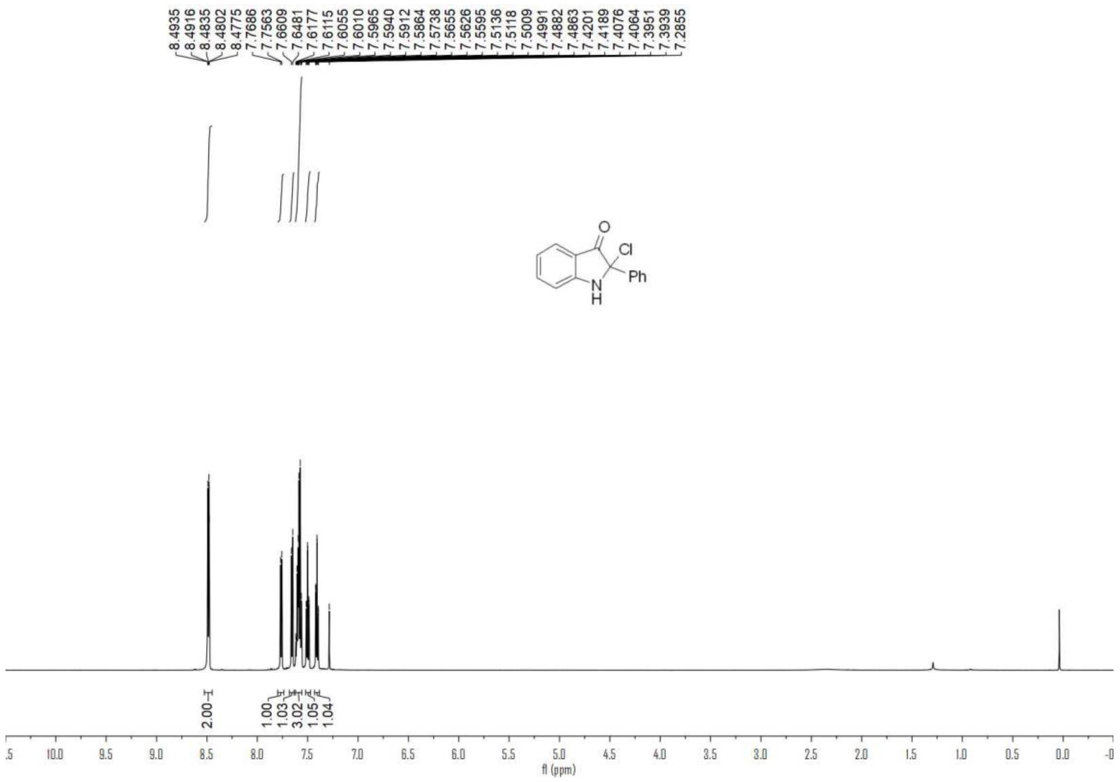

$^{13}C\{^{1}H\}$ NMR (151 MHz) of 2c in $CDCl_3$

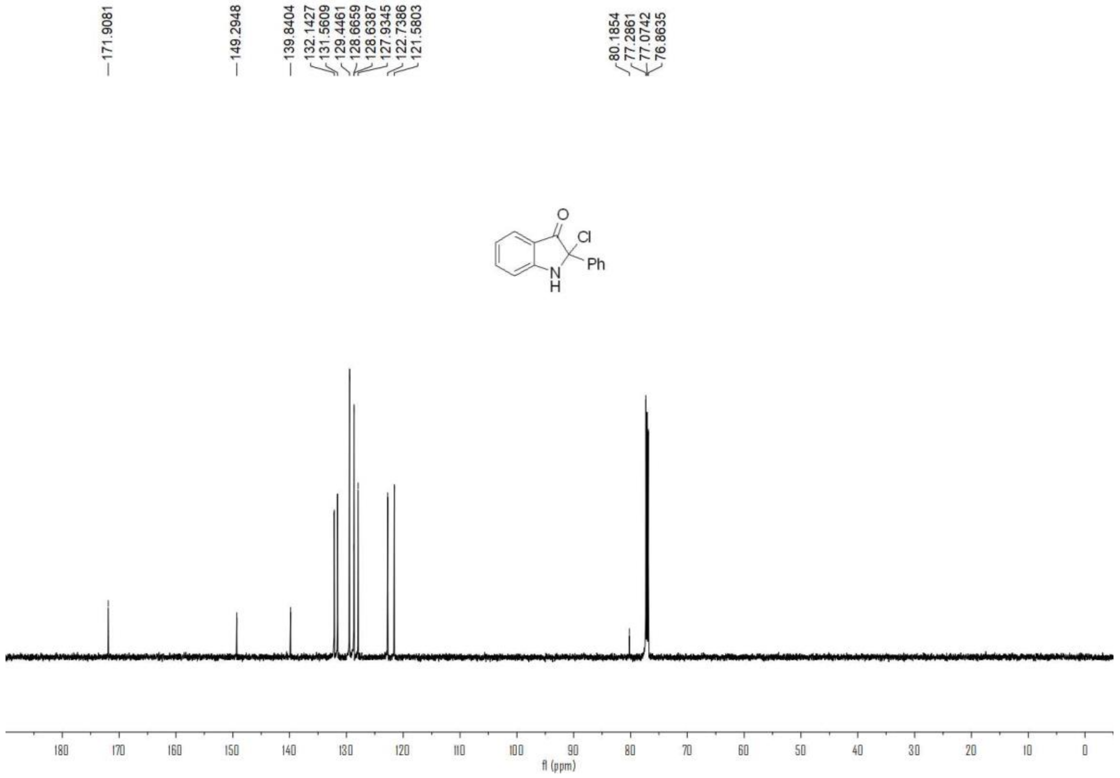

^{1}H NMR (600 MHz) of 2d in $CDCl_3$

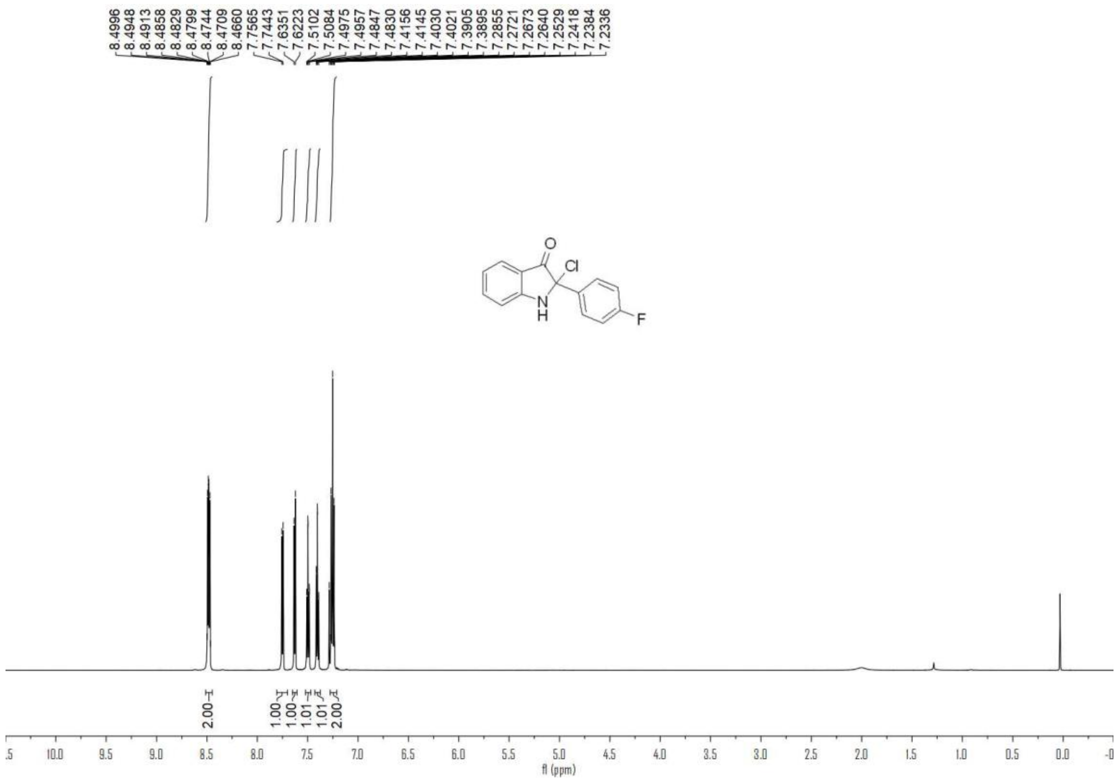

$^{13}C\{^1H\}$ NMR (151 MHz) of 2d in $CDCl_3$

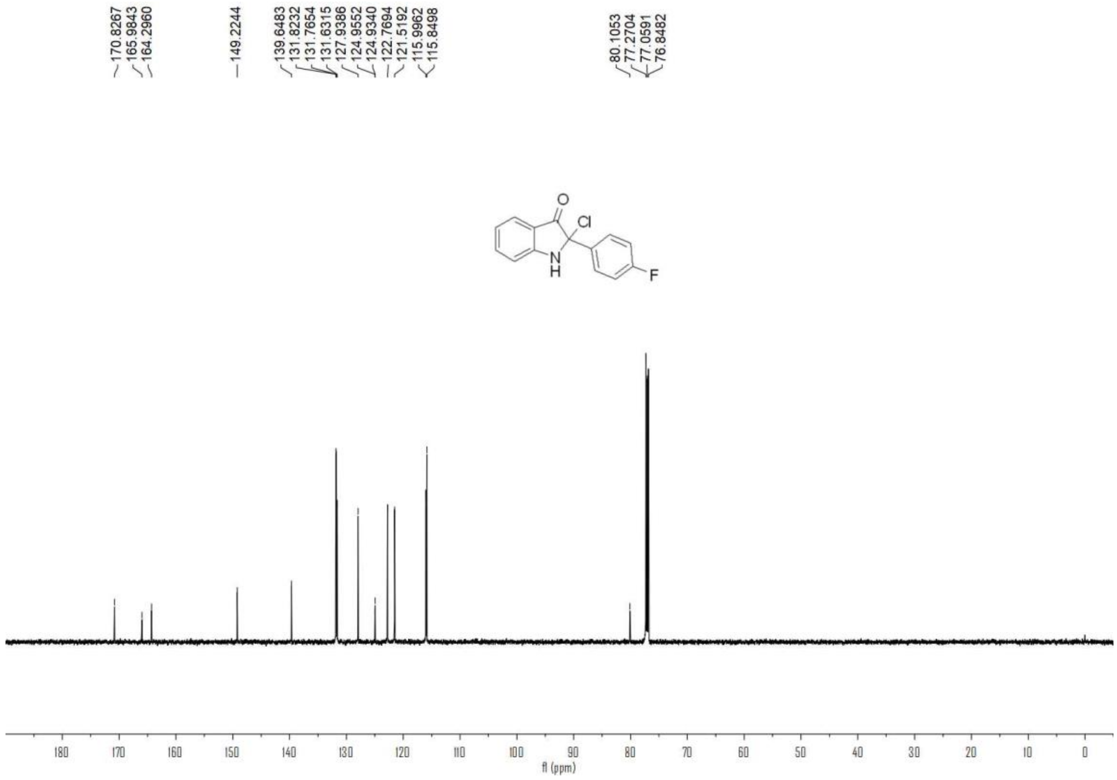

^{19}F NMR (565 MHz) of 2d in $CDCl_3$

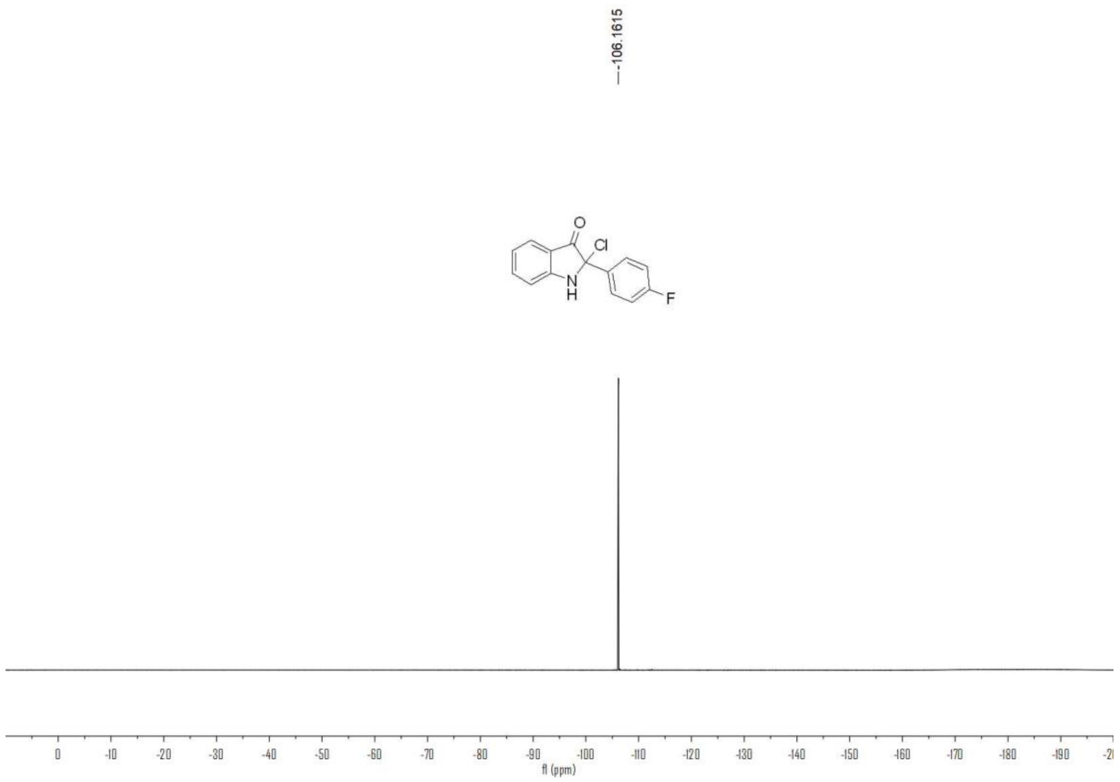

^{1}H NMR (600 MHz) of 2e in $CDCl_3$

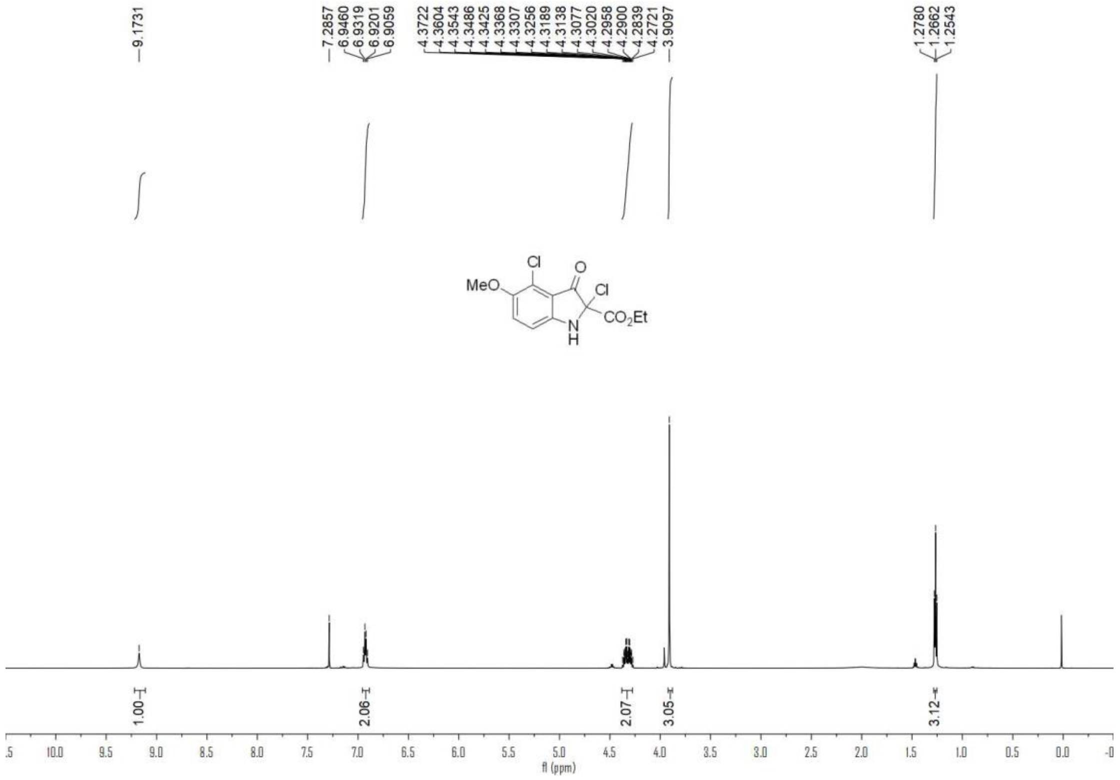

$^{13}C\{^1H\}$ NMR (151 MHz) of 2e in $CDCl_3$

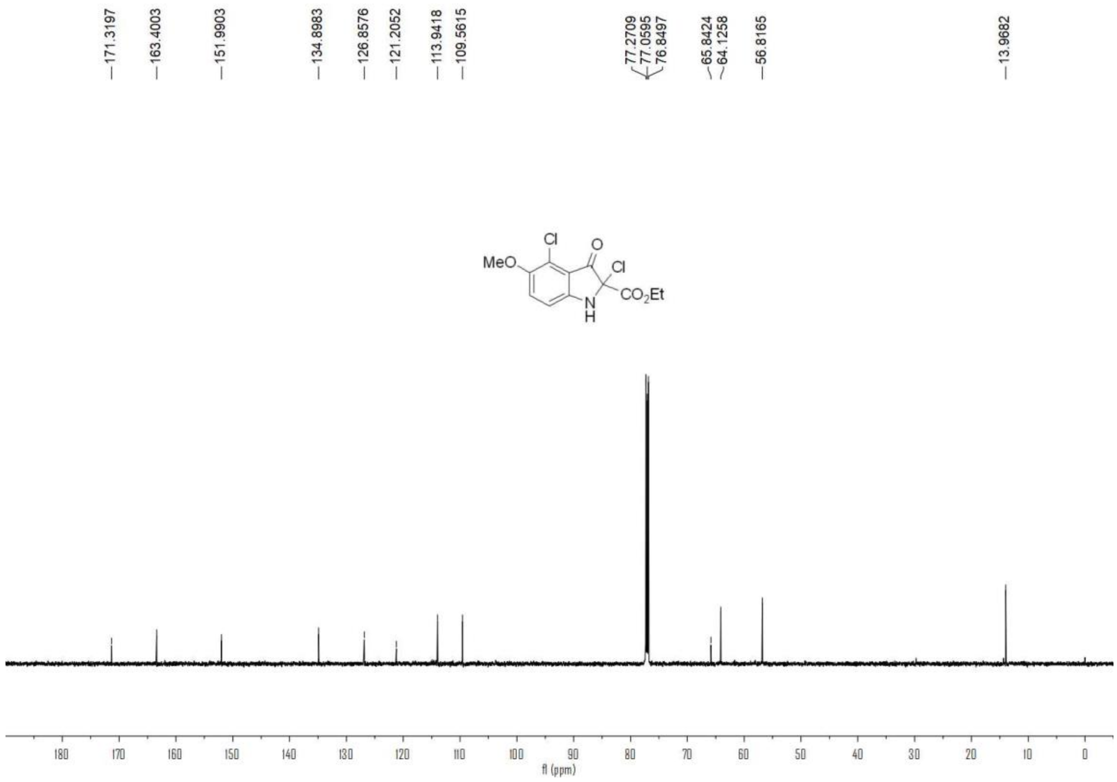

^1H NMR (500 MHz) of 3f in $CDCl_3$

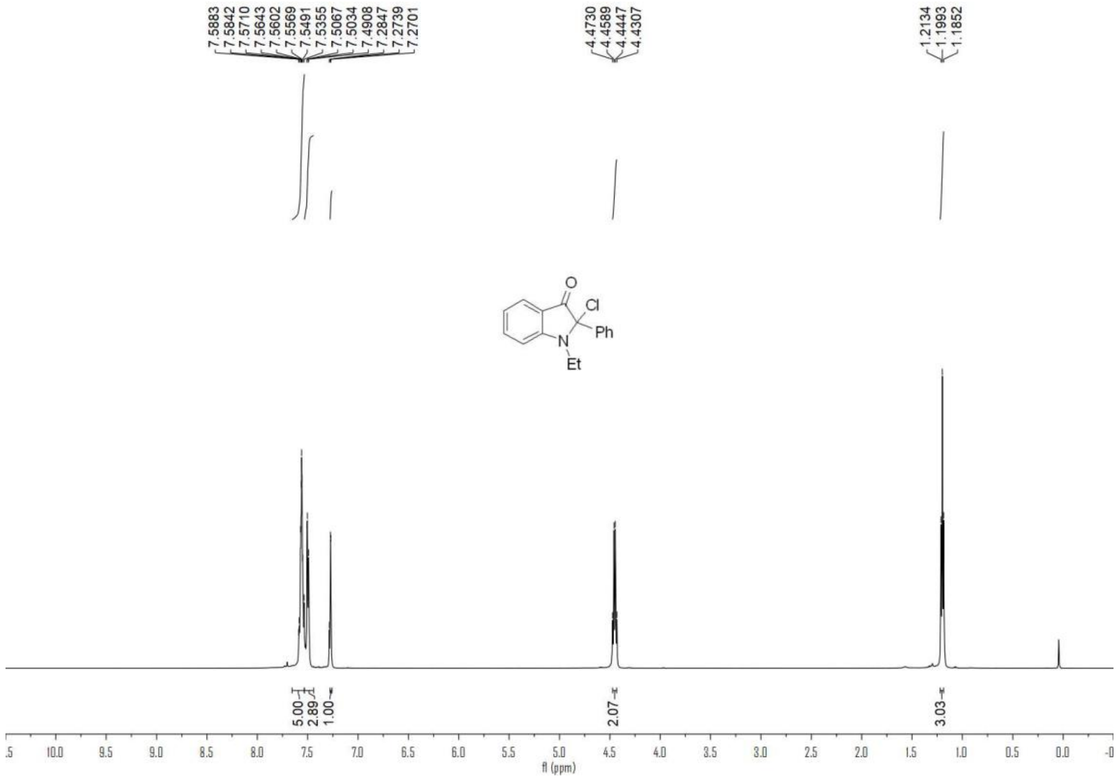

$^{13}C\{^{1}H\}$ NMR (126 MHz) of 3f in $CDCl_3$

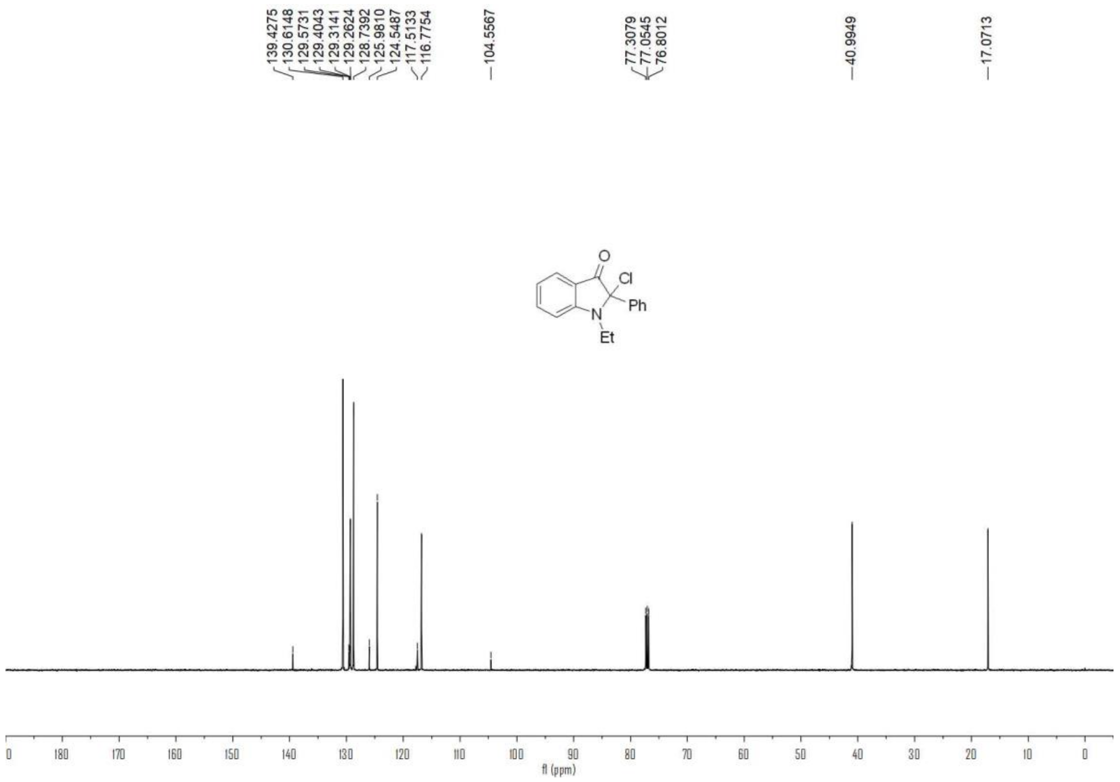

^{1}H NMR (500 MHz) of 3g in d_6-DMSO

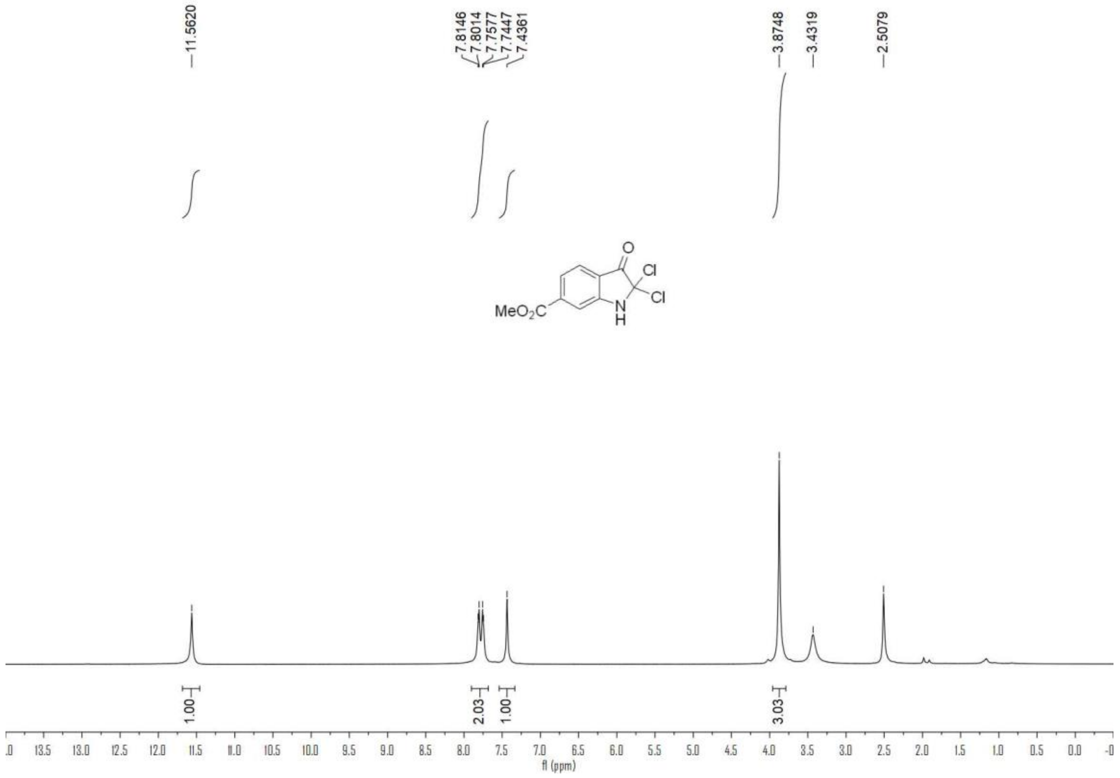

$^{13}C\{^1H\}$ NMR (126 MHz) of 3g in d_6-DMSO

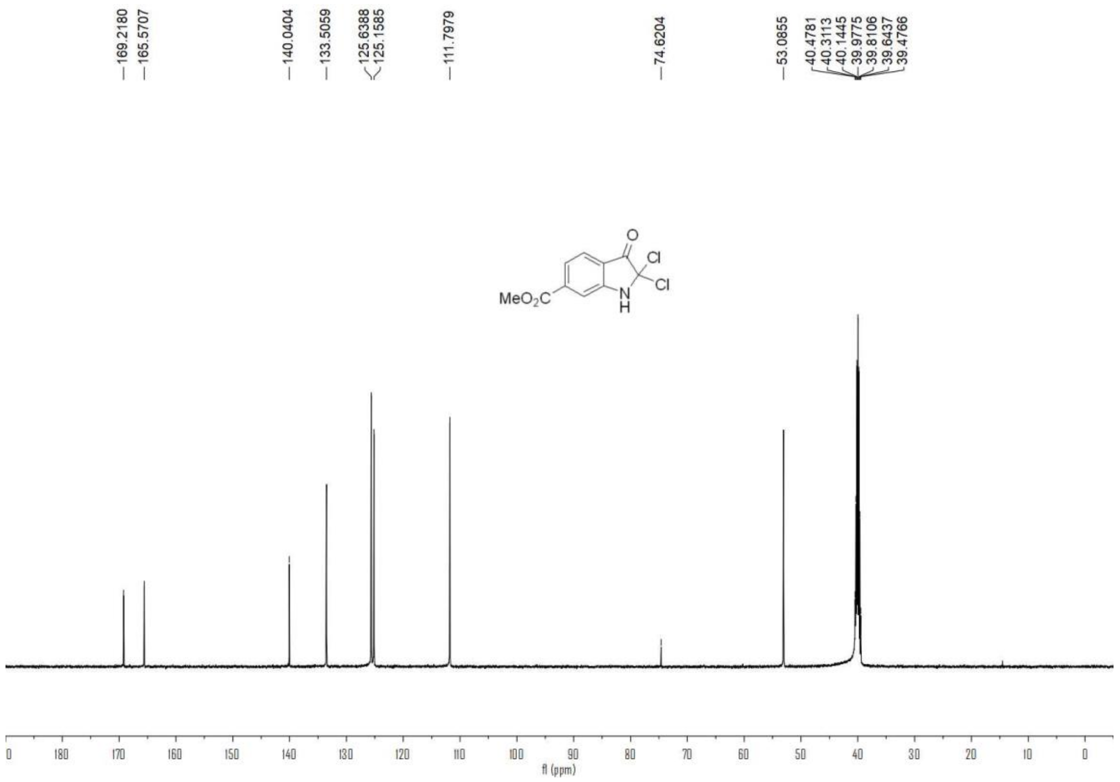

^1H NMR (500 MHz) of 3h in d_6-DMSO

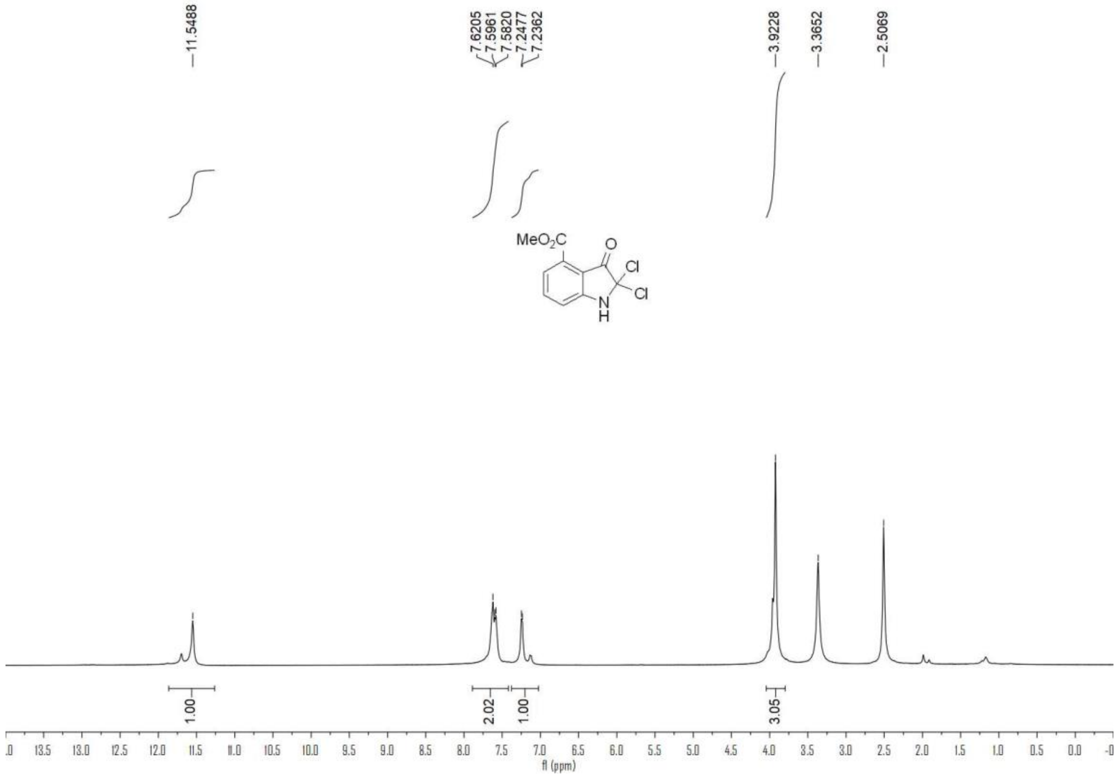

$^{13}C\{^{1}H\}$ NMR (126 MHz) of 3h in d_6-DMSO

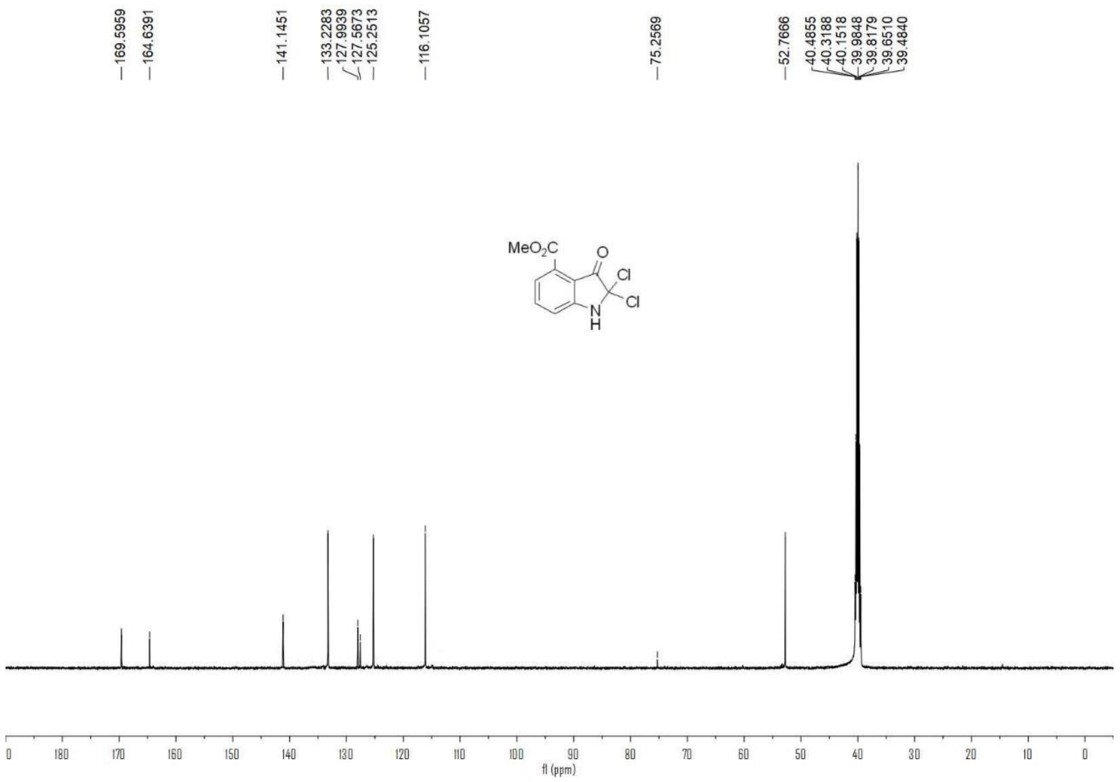

^{1}H NMR (500 MHz) of 3i in d_6-DMSO

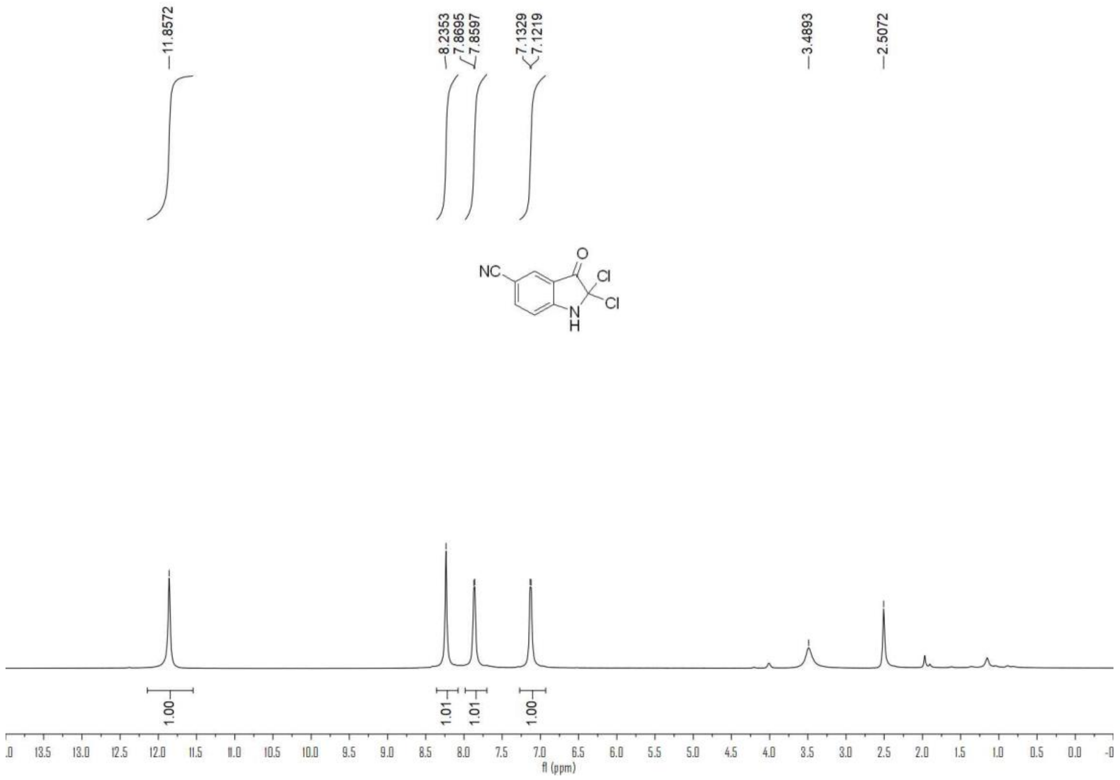

$^{13}C\{^{1}H\}$ NMR (126 MHz) of 3i in d_6-DMSO

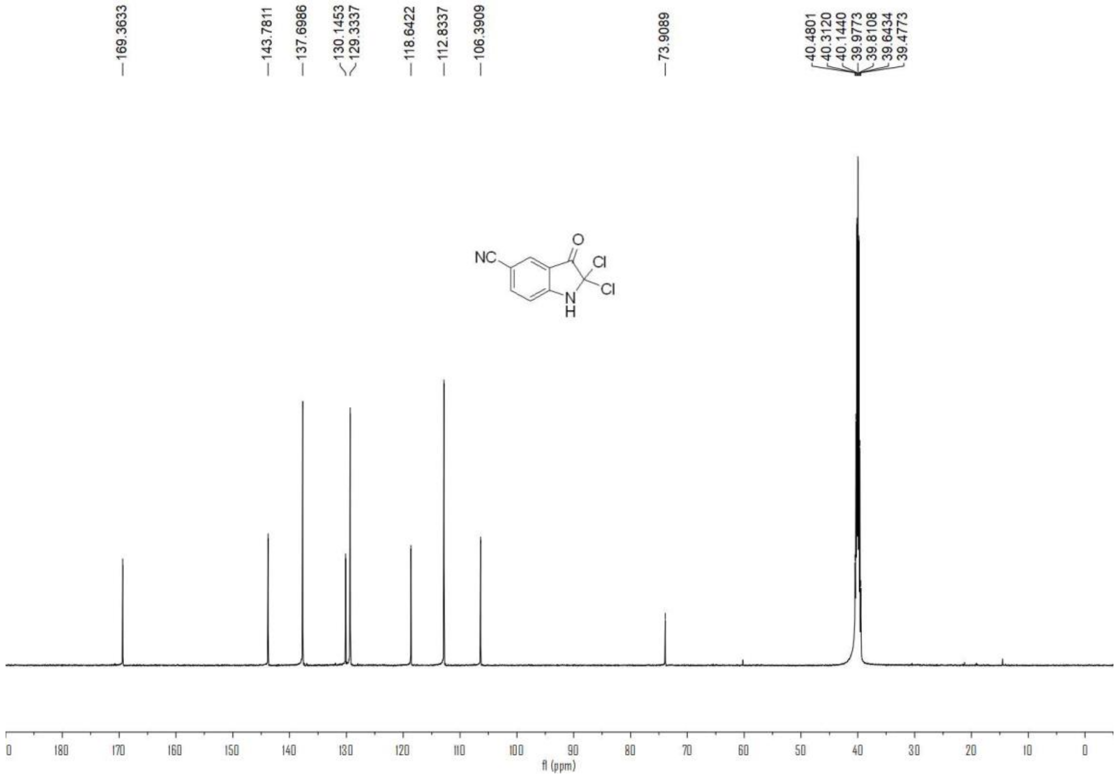

^{1}H NMR (500 MHz) of 3j in d_6-DMSO

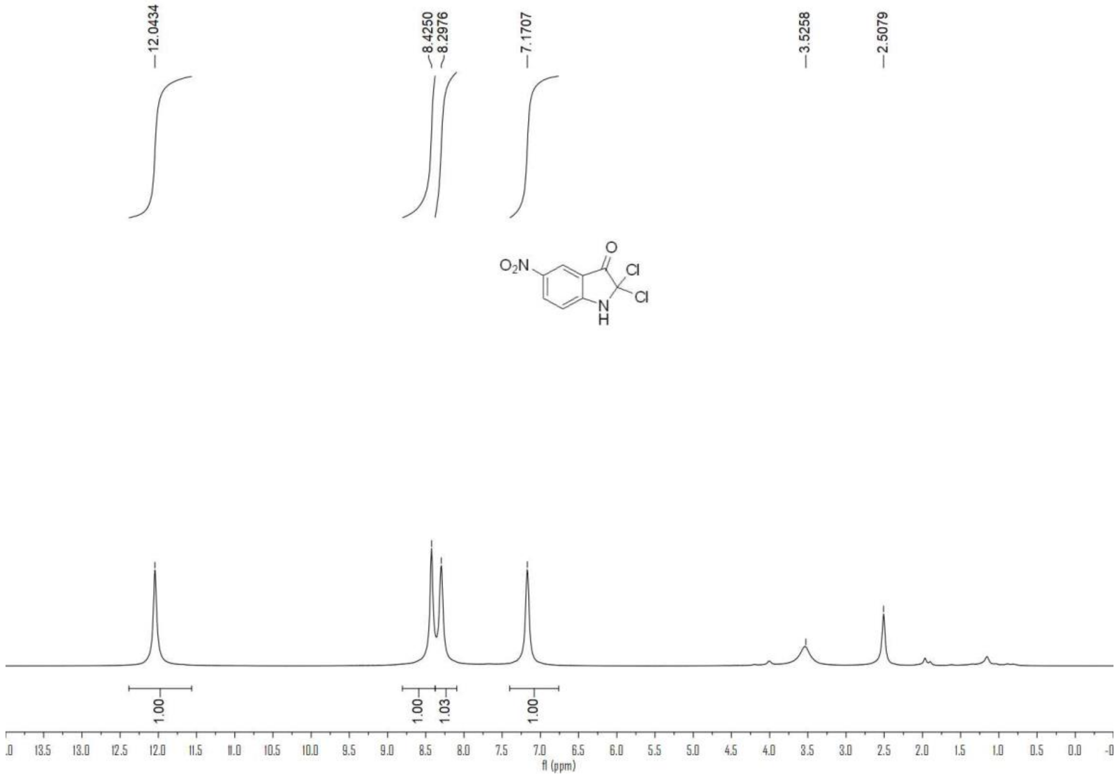

$^{13}C\{^1H\}$ NMR (126 MHz) of 3j in d_6-DMSO

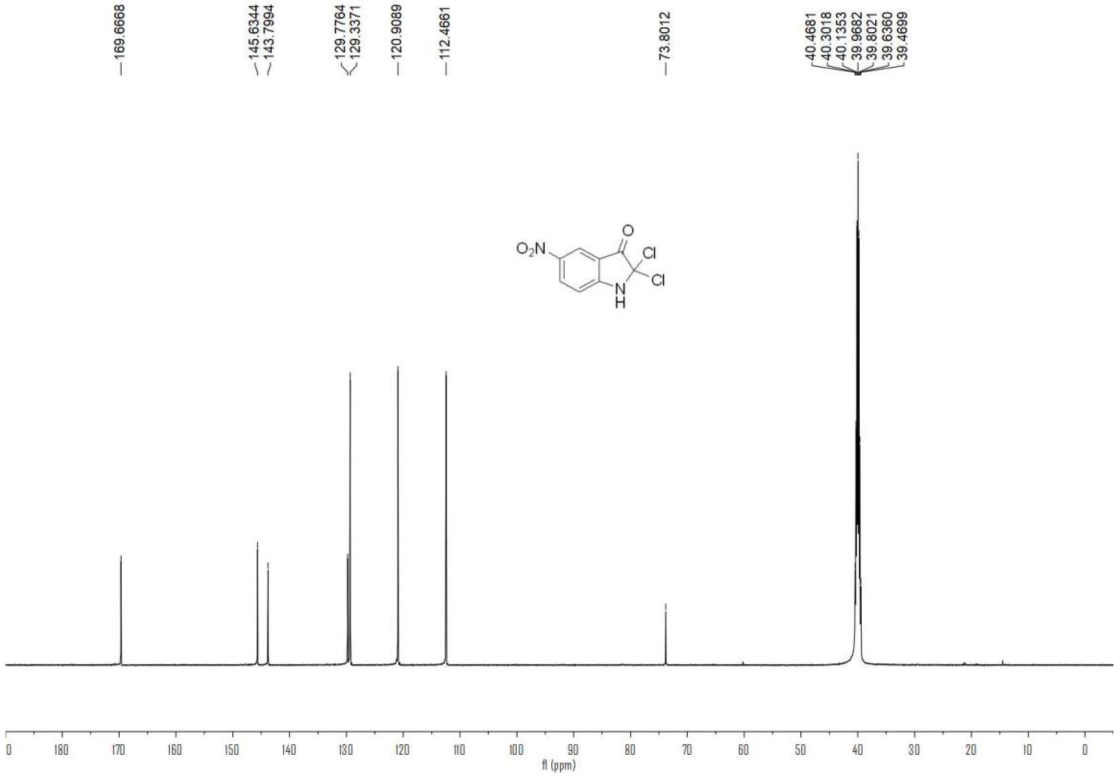

^1H NMR (500 MHz) of 3k in $CDCl_3$

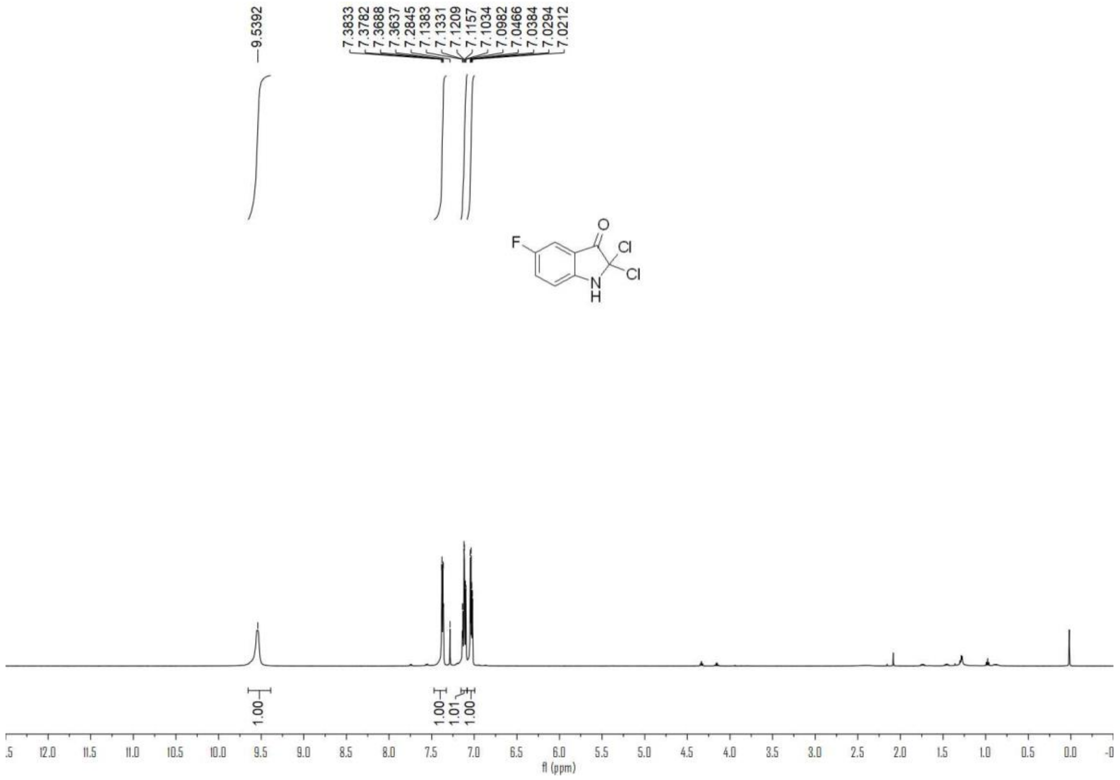

$^{13}C\{^1H\}$ NMR (126 MHz) of 3k in $CDCl_3$

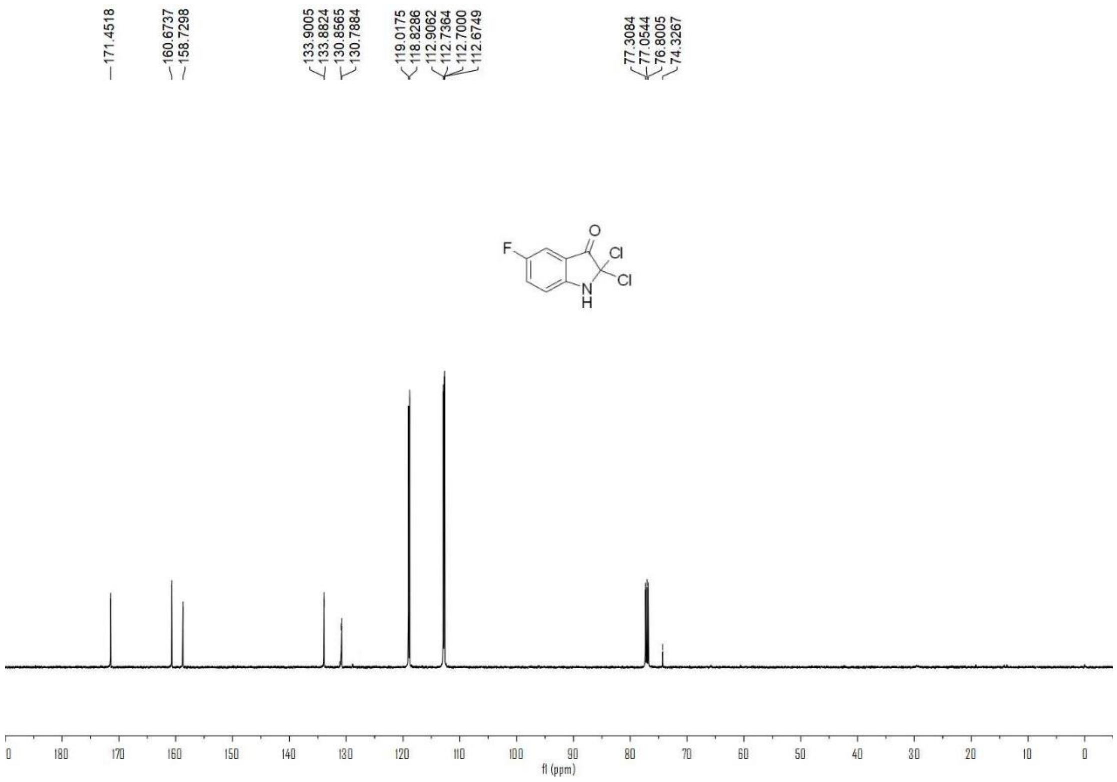

^{19}F NMR (471 MHz) of 3k in $CDCl_3$

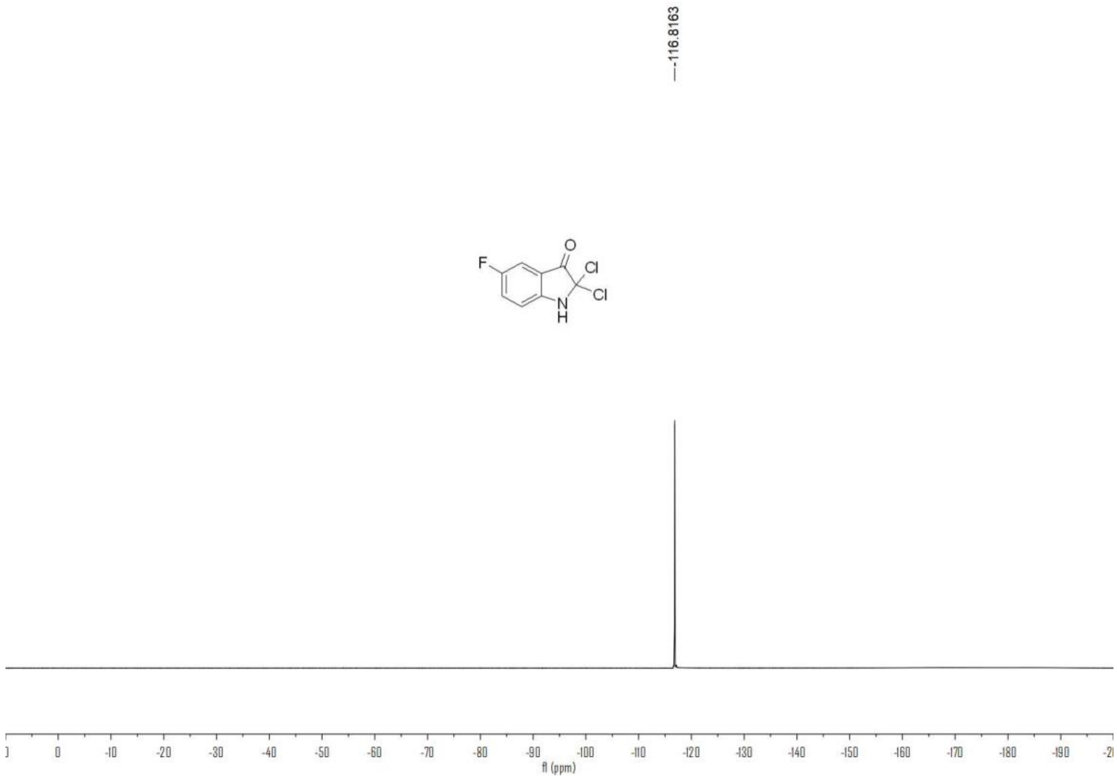

^{1}H NMR (500 MHz) of 3l in d_6-DMSO

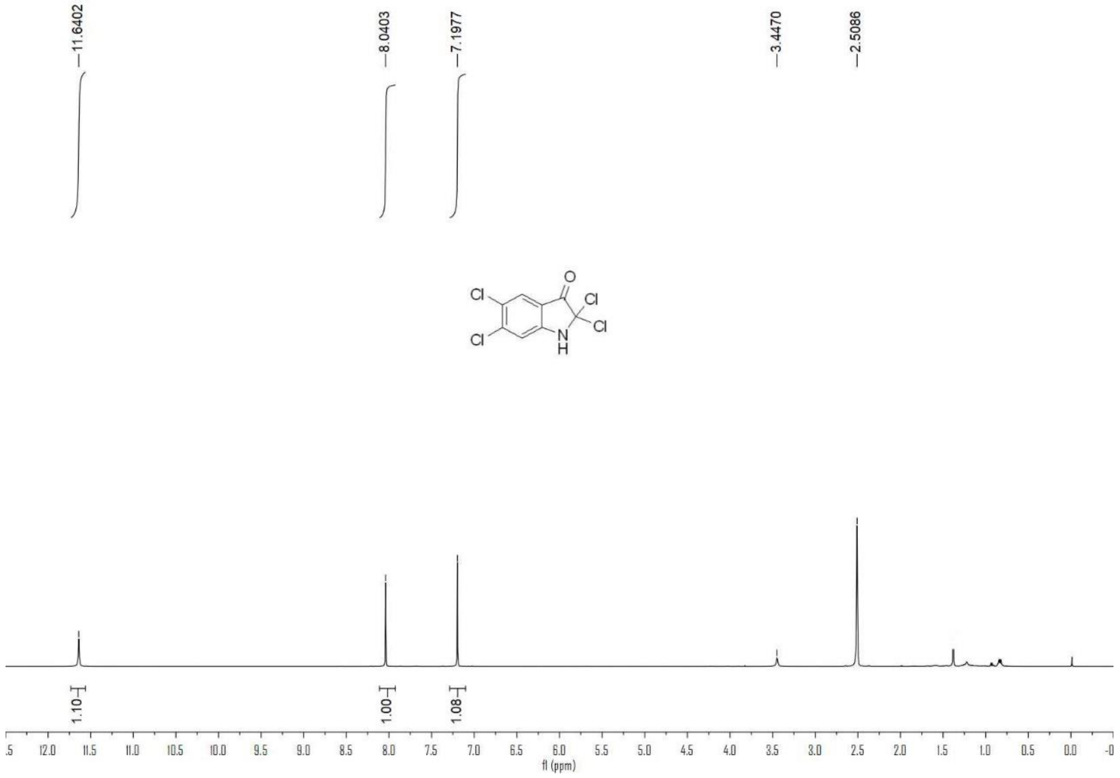

$^{13}C\{^{1}H\}$ NMR (126 MHz) of 3l in d_6-DMSO

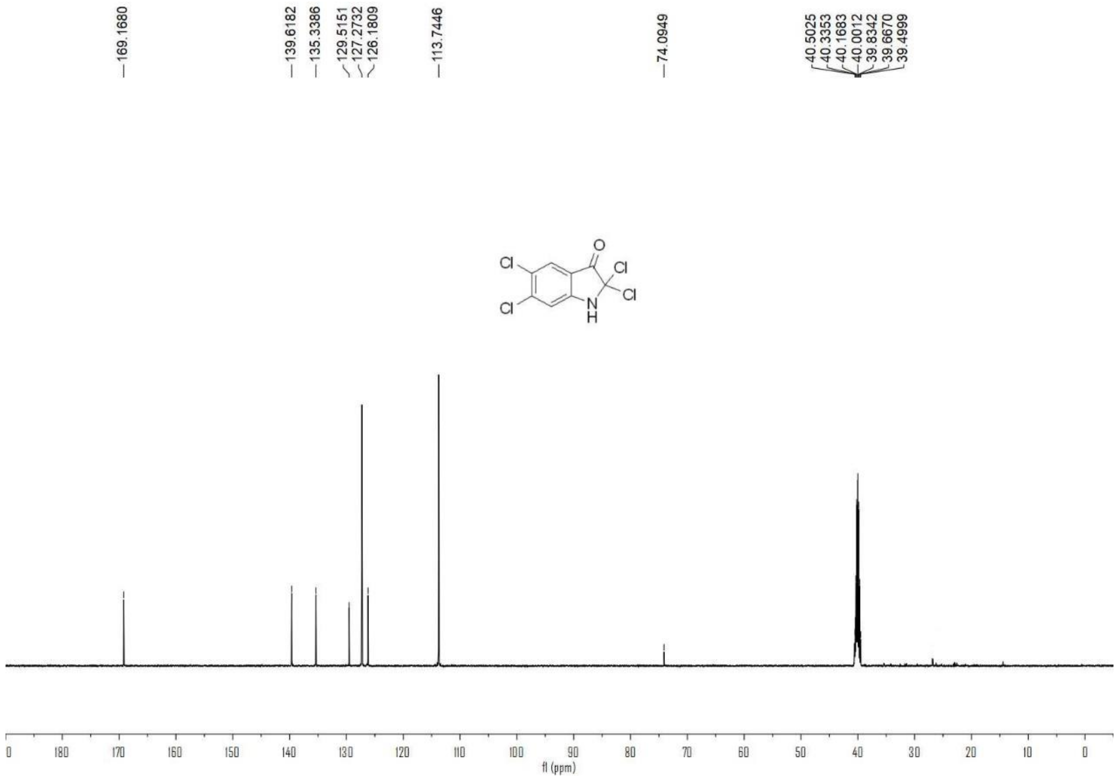

^{1}H NMR (500 MHz) of 3m in d_6-DMSO

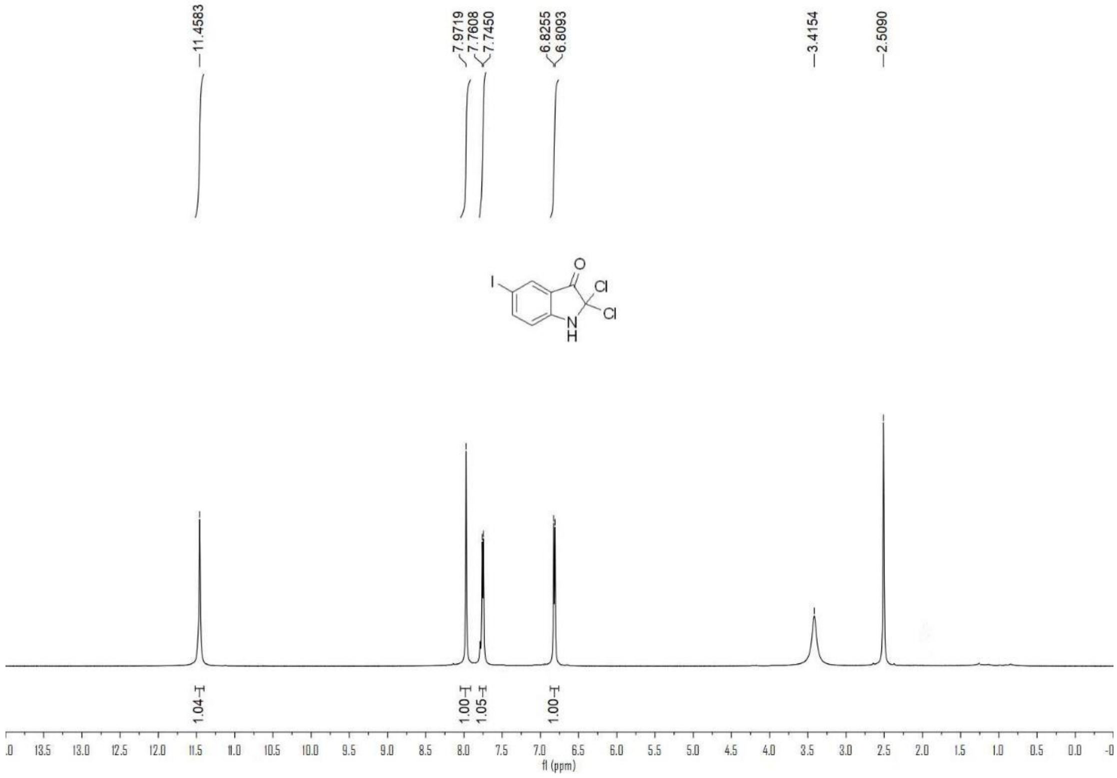

^{13}C{^{1}H} NMR (126 MHz) of 3m in d_6-DMSO

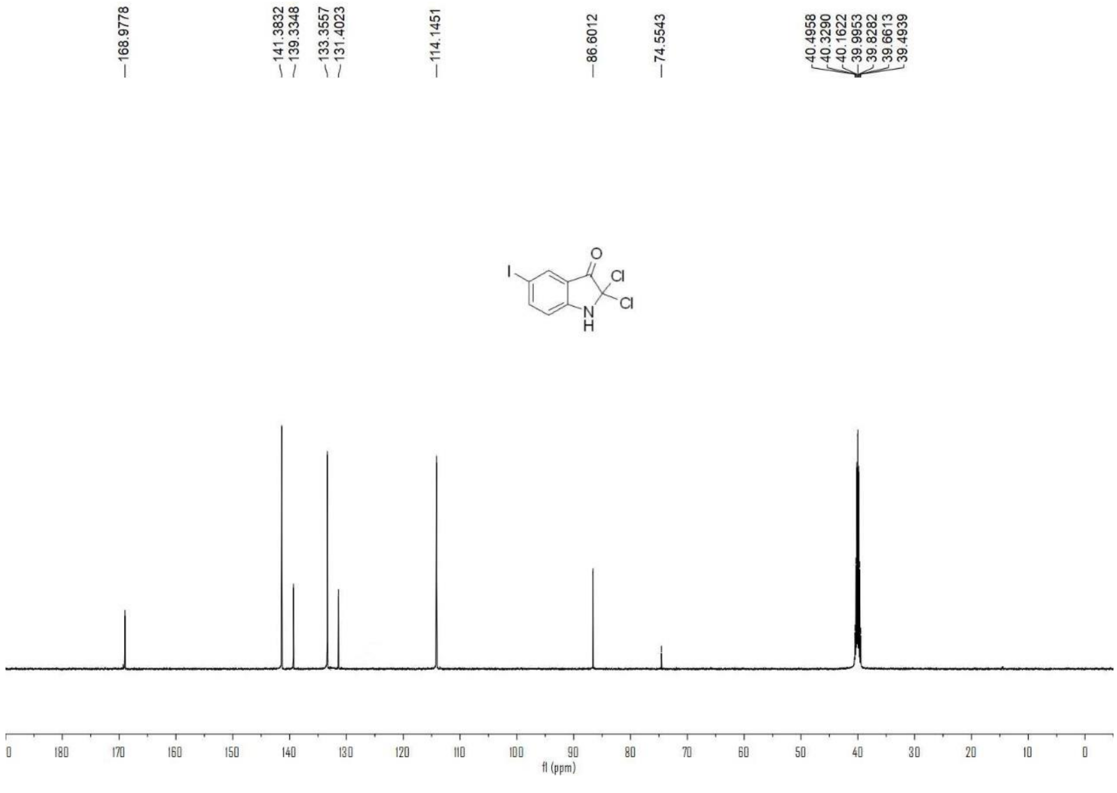

^{1}H NMR (500 MHz) of 3n in $CDCl_3$

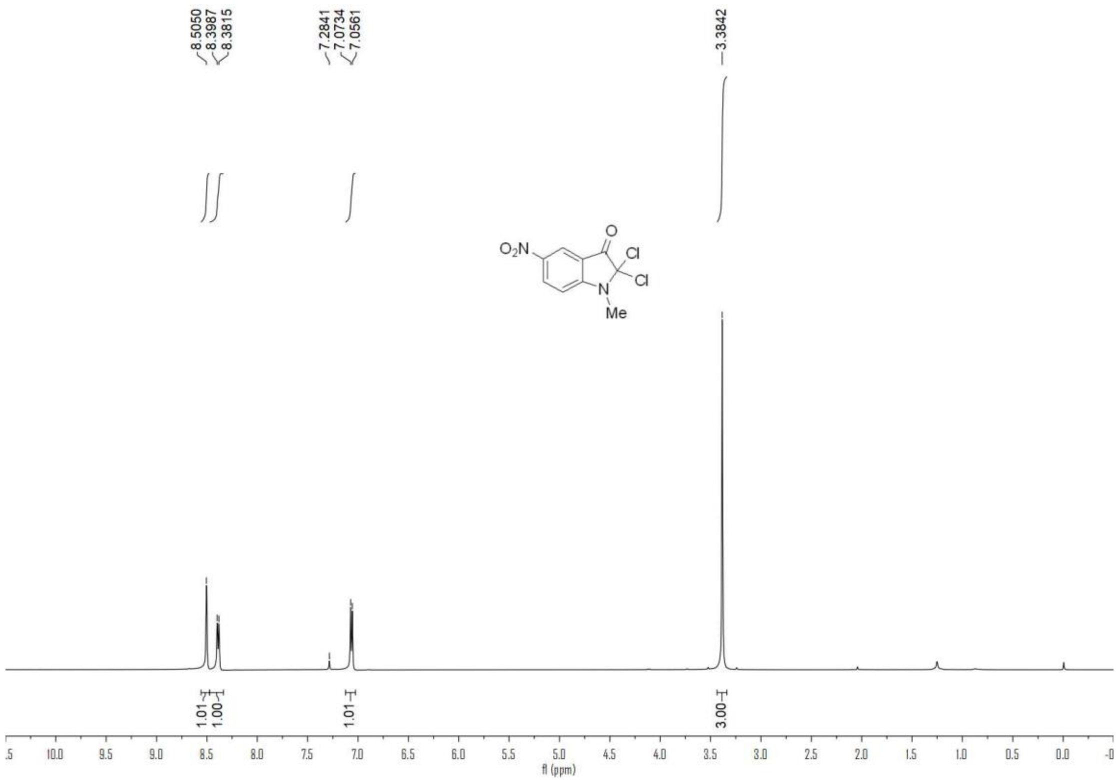

^{13}C{^{1}H} NMR (126 MHz) of 3n in $CDCl_3$

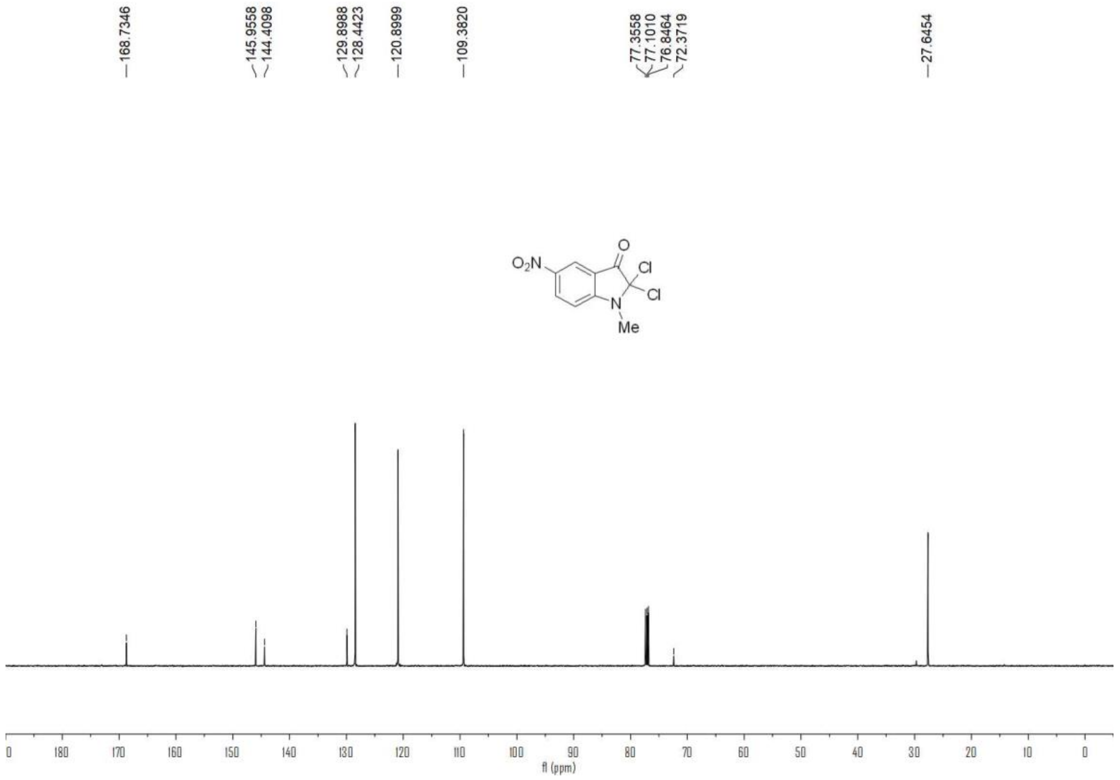

^{1}H NMR (600 MHz) of 3o in $CDCl_3$

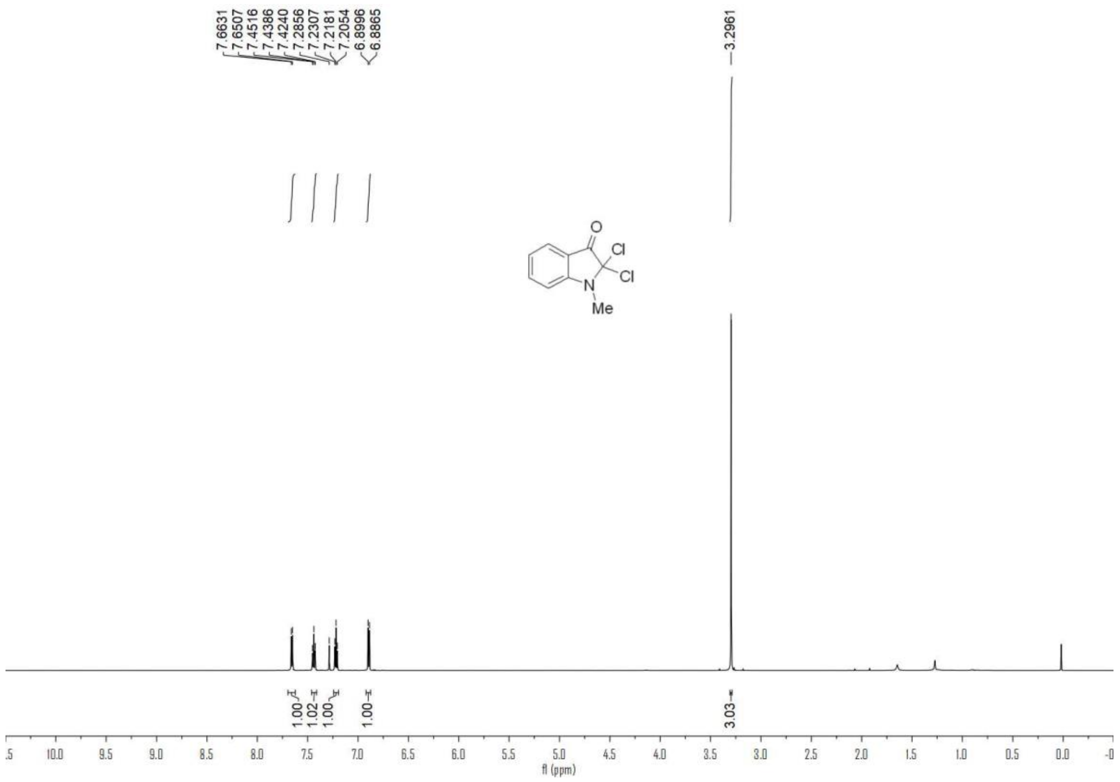

$^{13}C\{^{1}H\}$ NMR (151 MHz) of 3o in $CDCl_3$

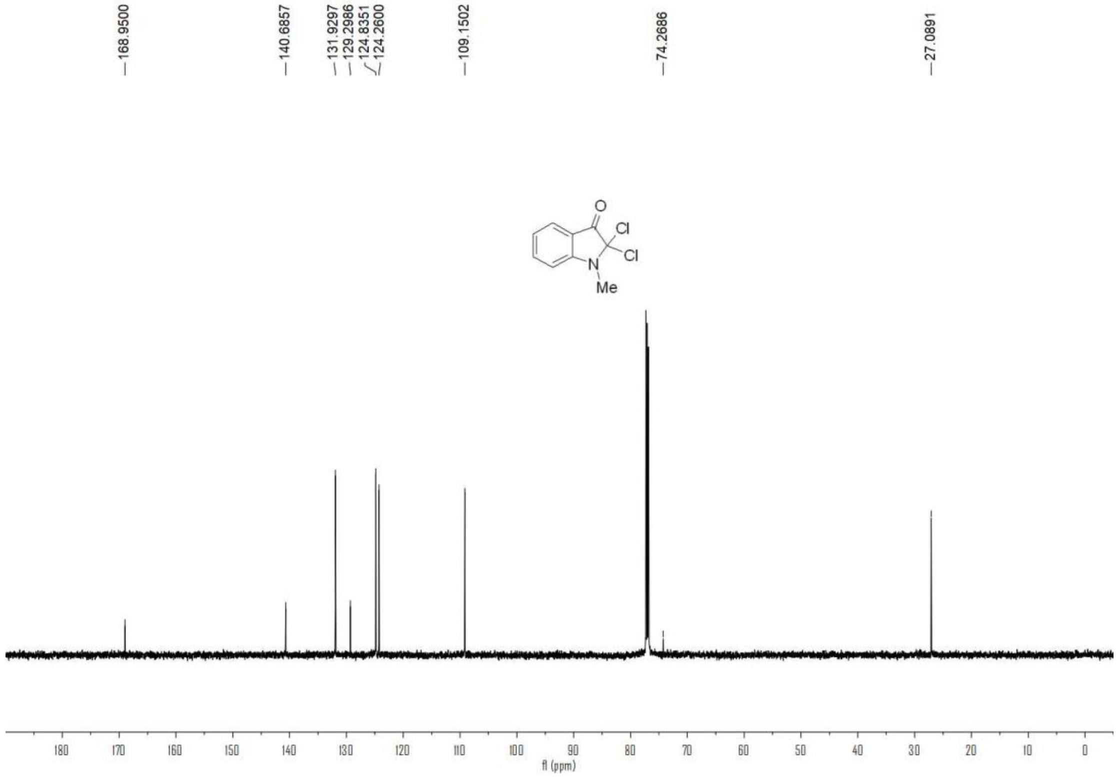

^{1}H NMR (600 MHz) of 3p in $CDCl_3$

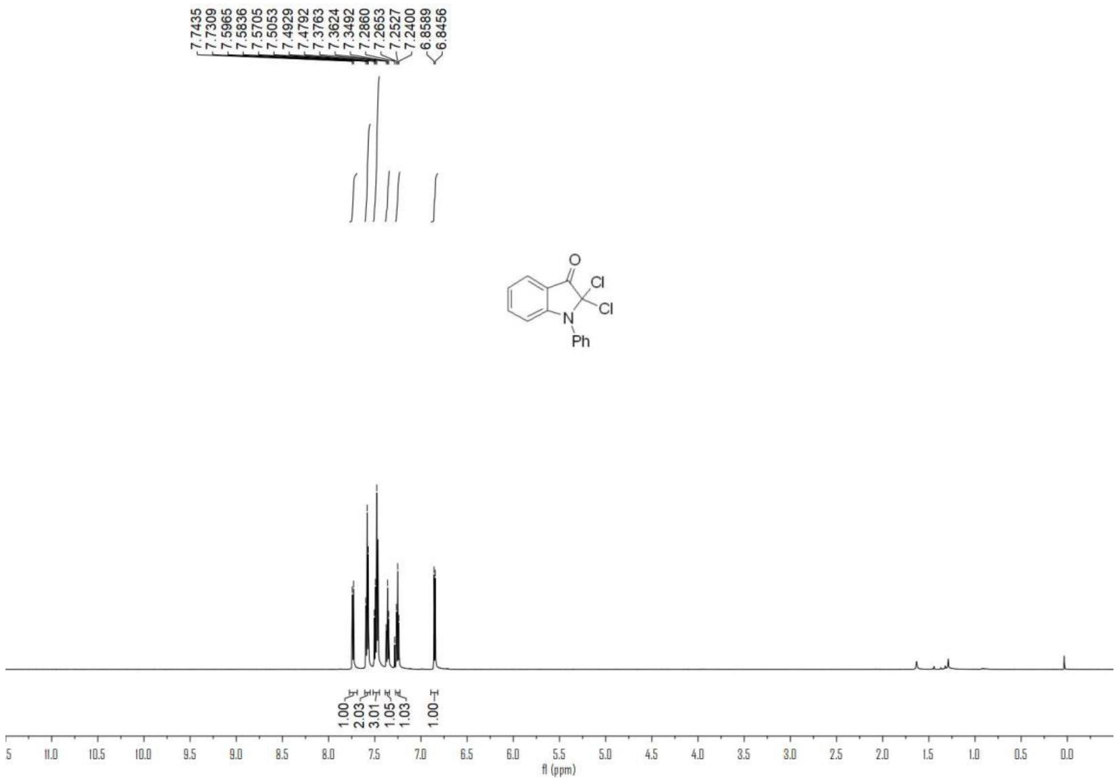

$^{13}C\{^{1}H\}$ NMR (151 MHz) of 3p in $CDCl_3$

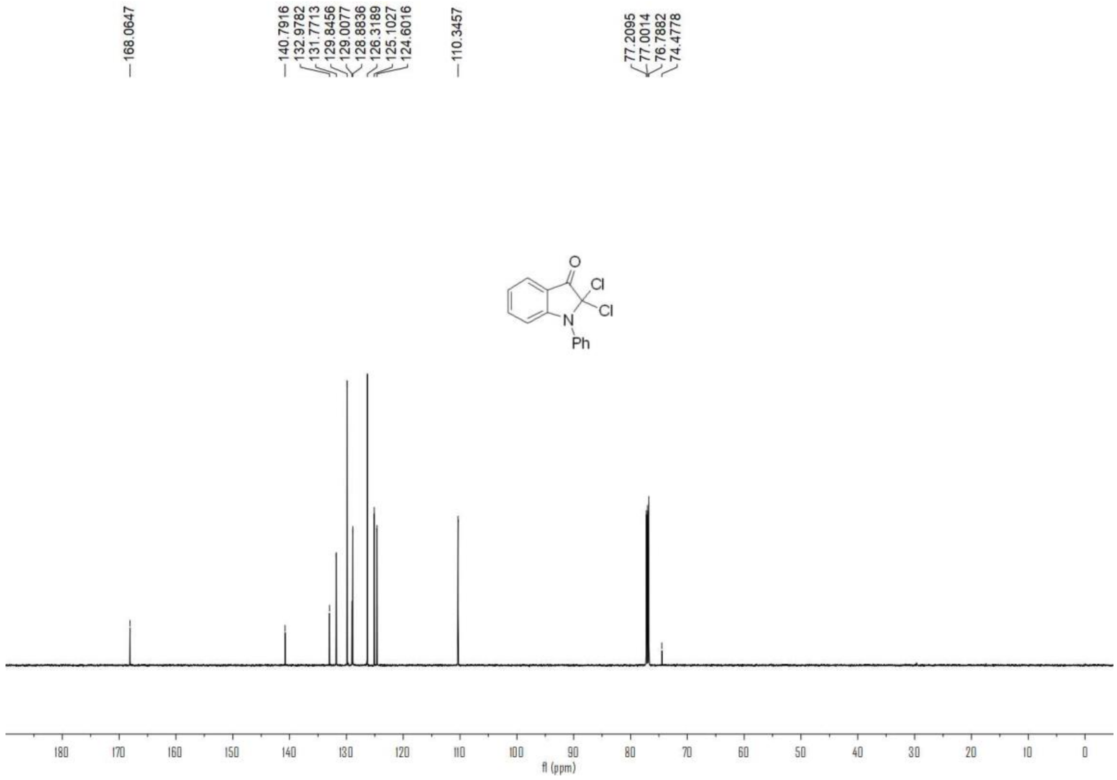

^{1}H NMR (500 MHz) of 3q in $CDCl_3$

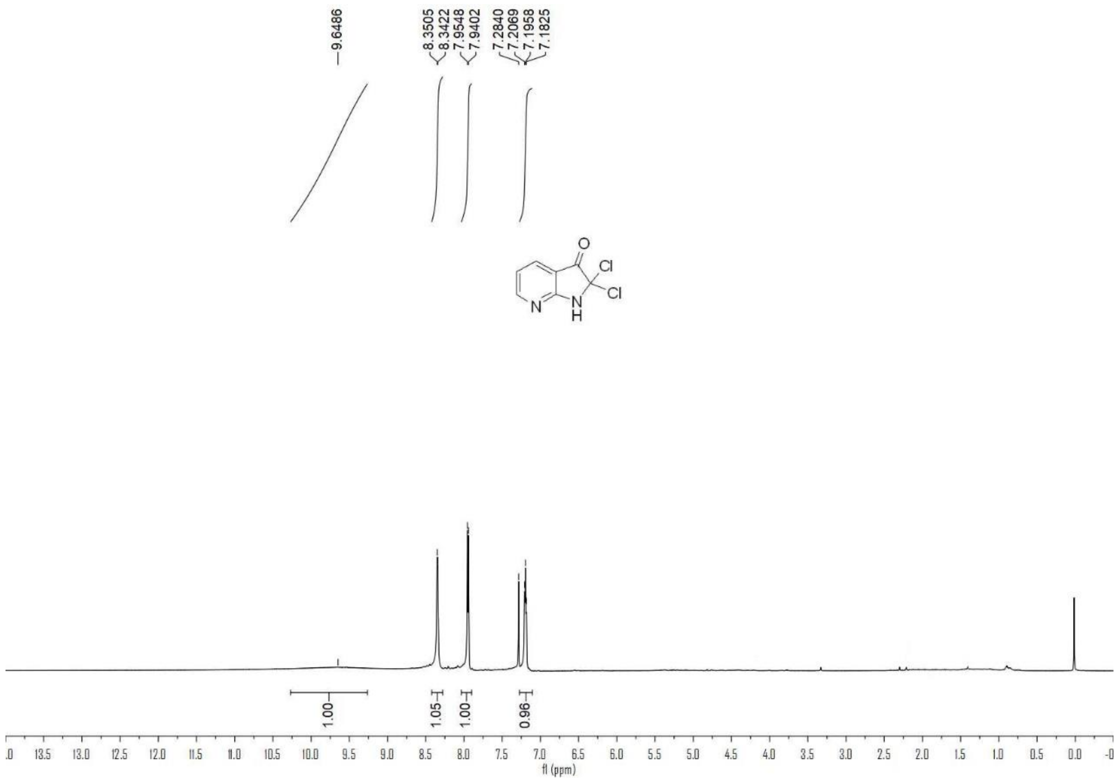

$^{13}C\{^1H\}$ NMR (126 MHz) of 3q in $CDCl_3$

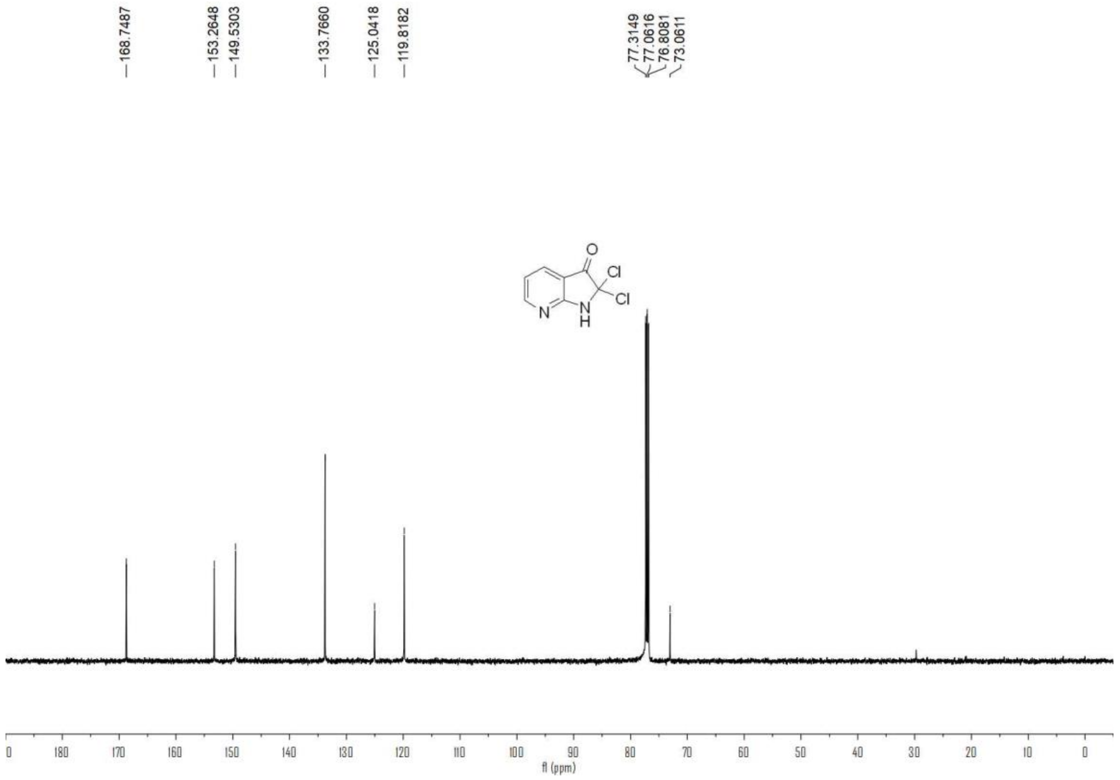

^{1}H NMR (500 MHz) of 5a in d_6-DMSO

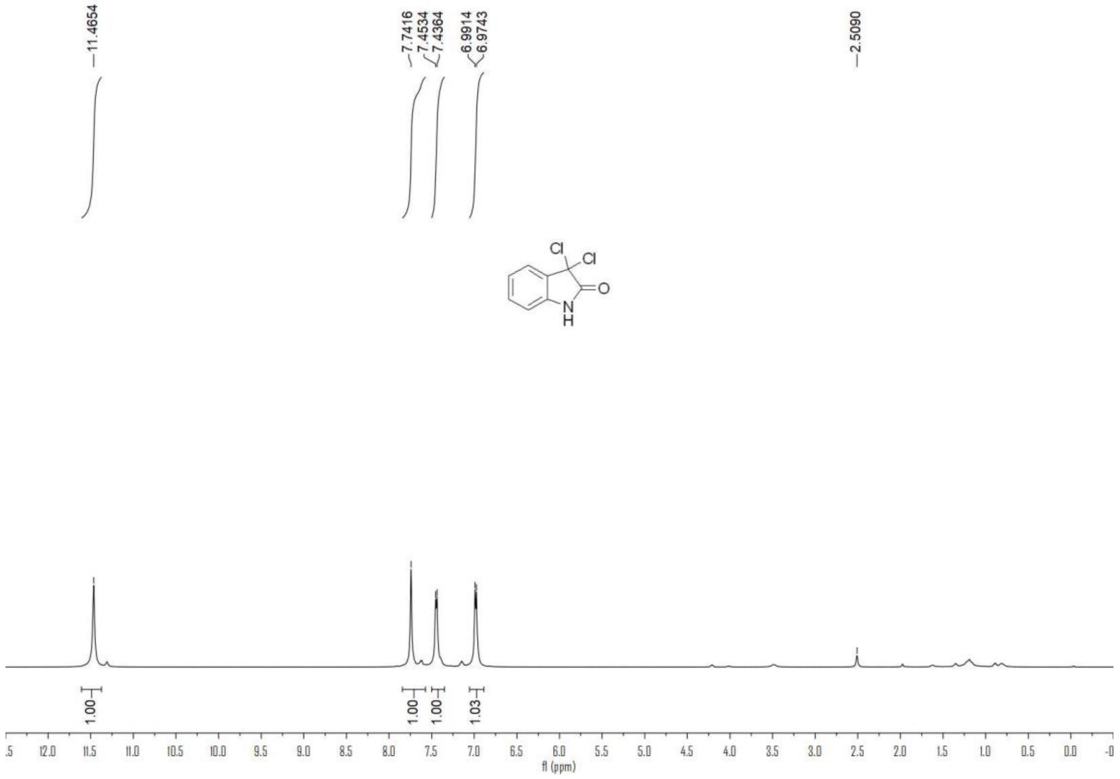

$^{13}C\{^{1}H\}$ NMR (126 MHz) of 5a in d_6-DMSO

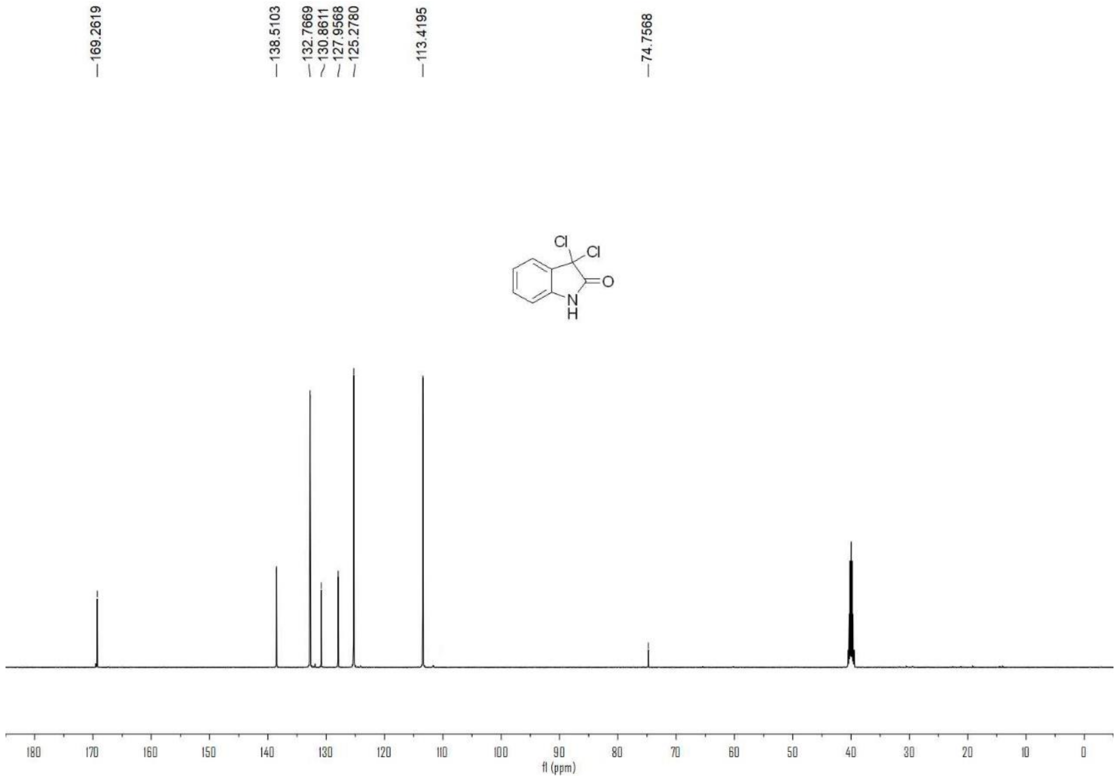

^{1}H NMR (500 MHz) of 5b in d_6-DMSO

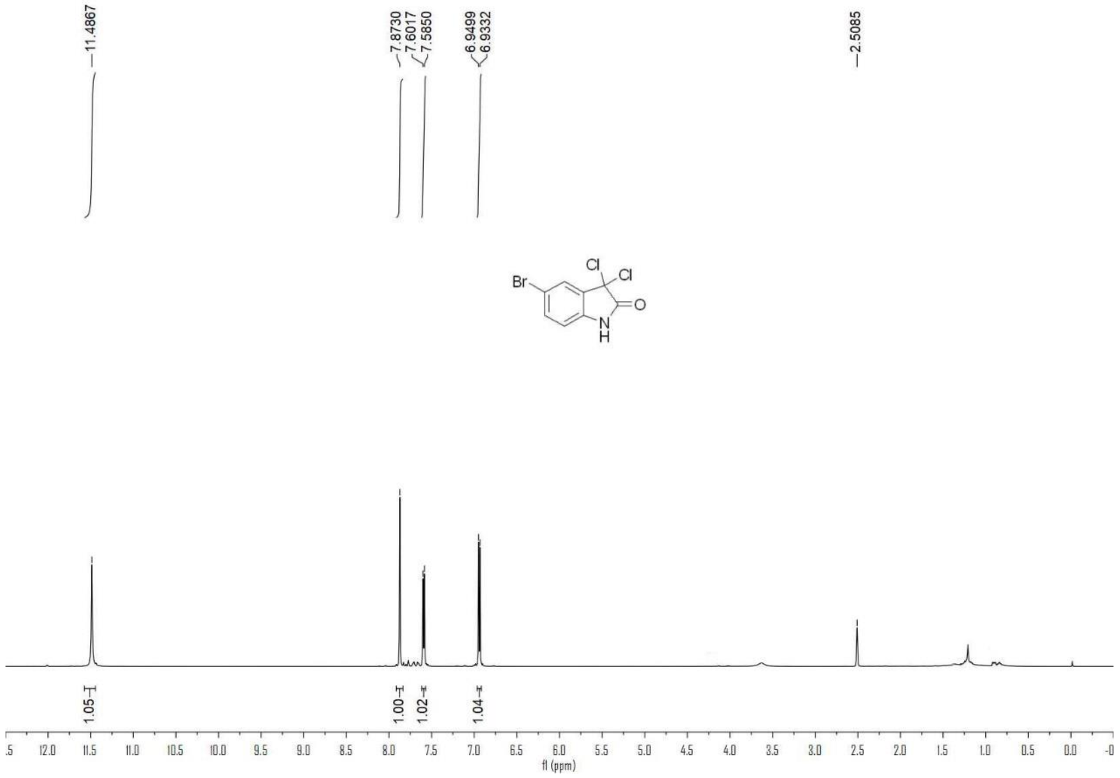

^{13}C{^{1}H} NMR (126 MHz) of 5b in d_6-DMSO

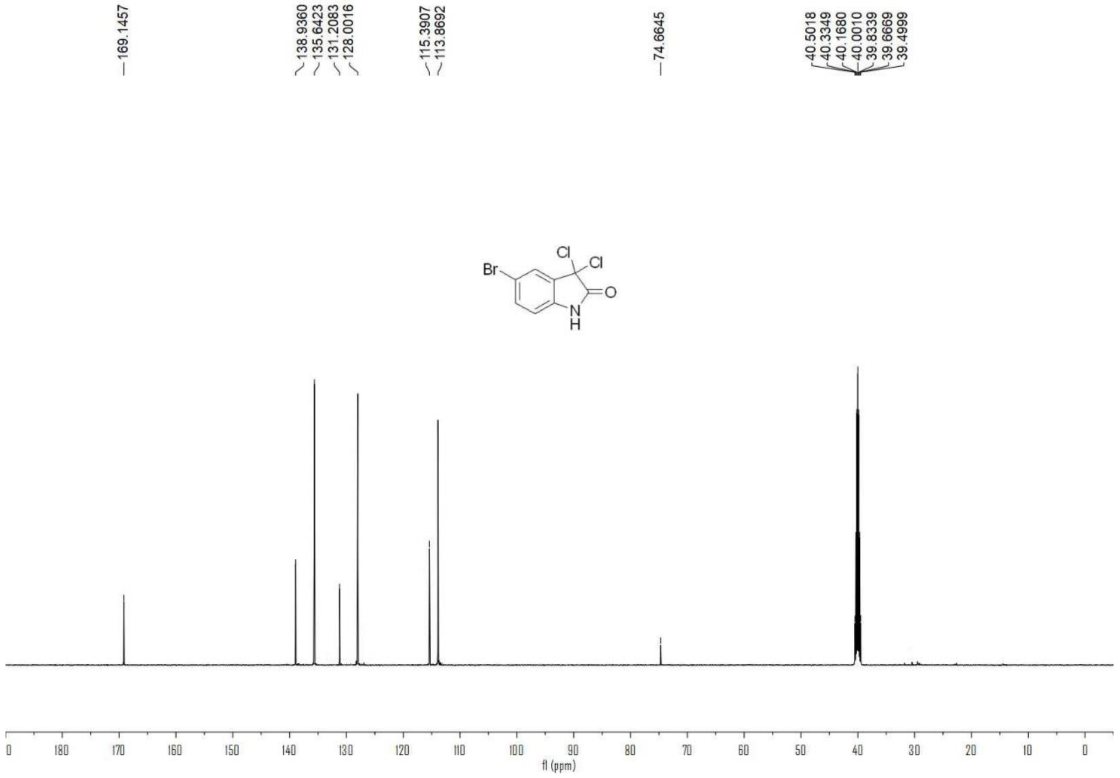

^{1}H NMR (500 MHz) of 5c in d_6-DMSO

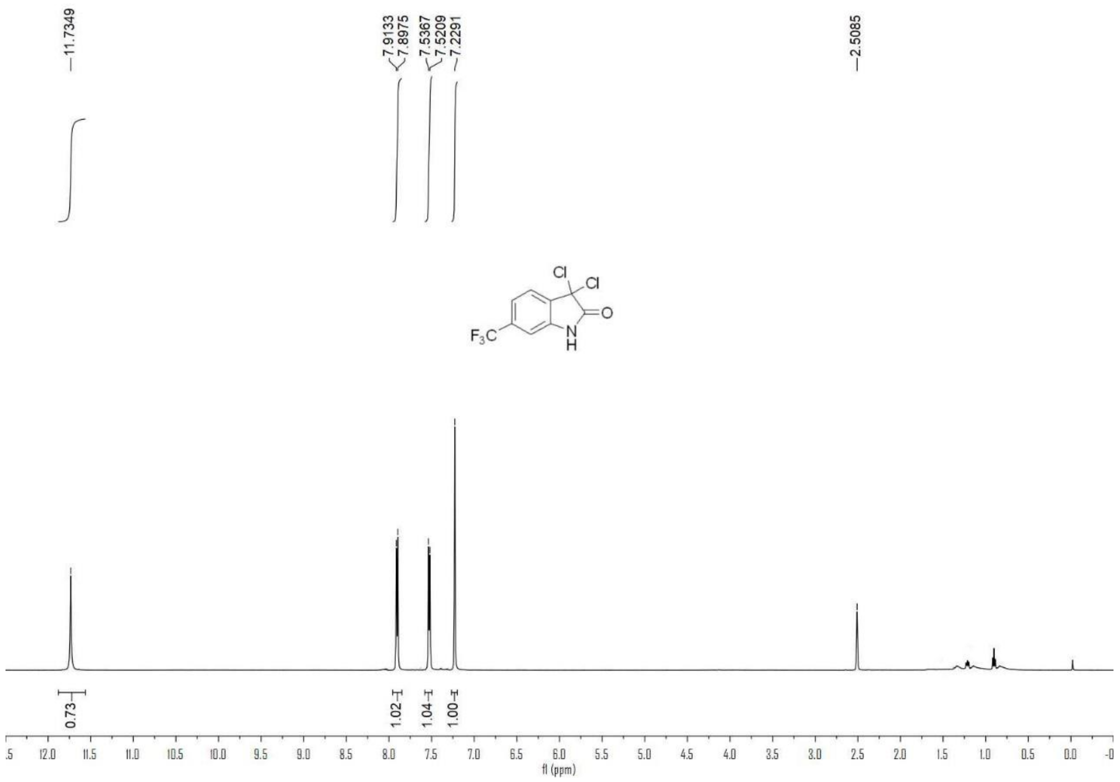

^{13}C{^{1}H} NMR (126 MHz) of 5c in d_6-DMSO

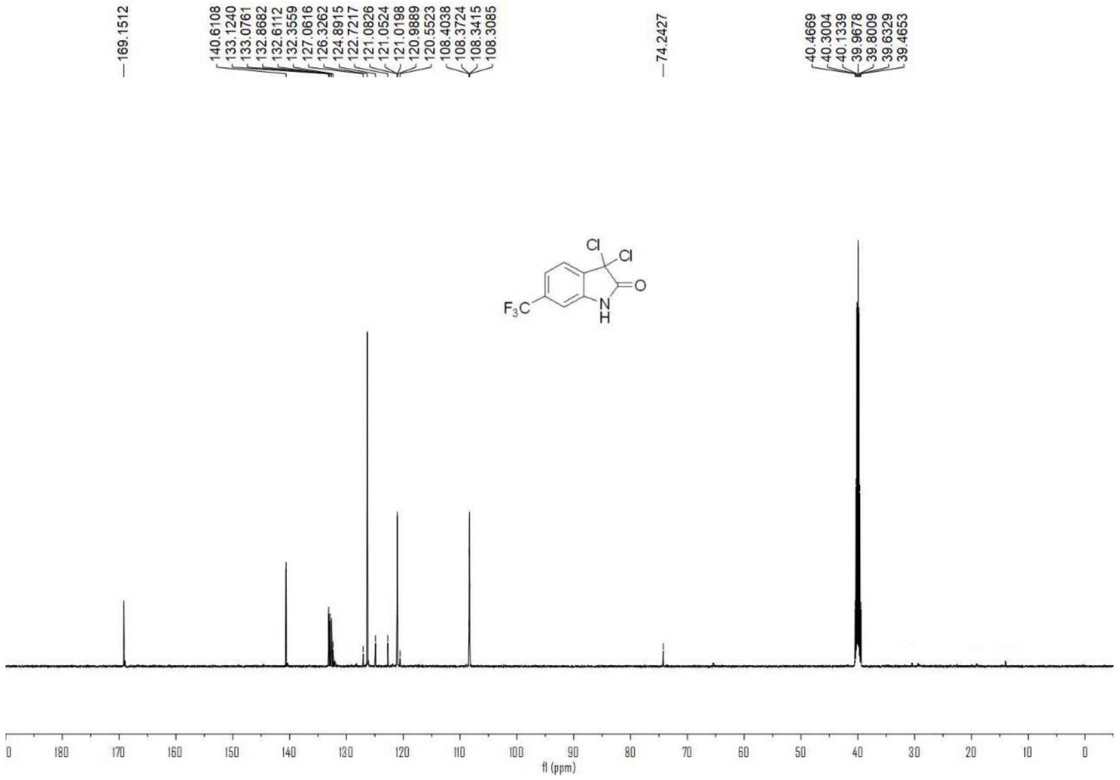

^{19}F NMR (471 MHz) of 5c in d_6-DMSO

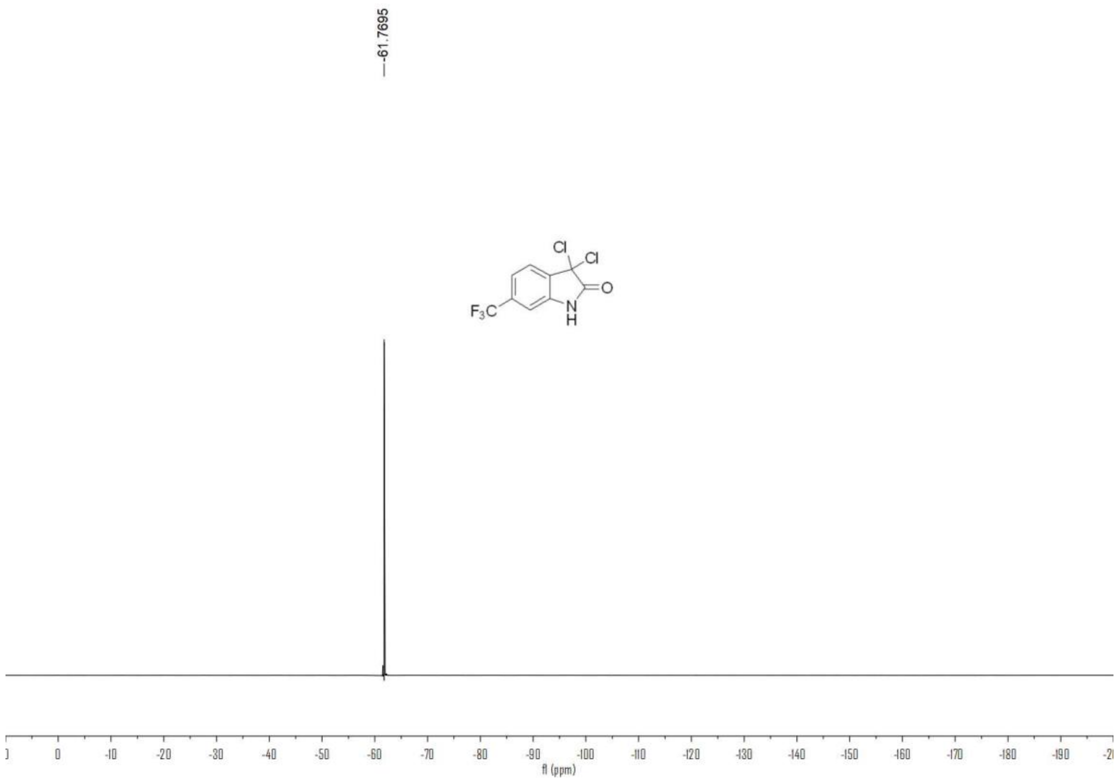

^{1}H NMR (500 MHz) of 5d in d_6-DMSO

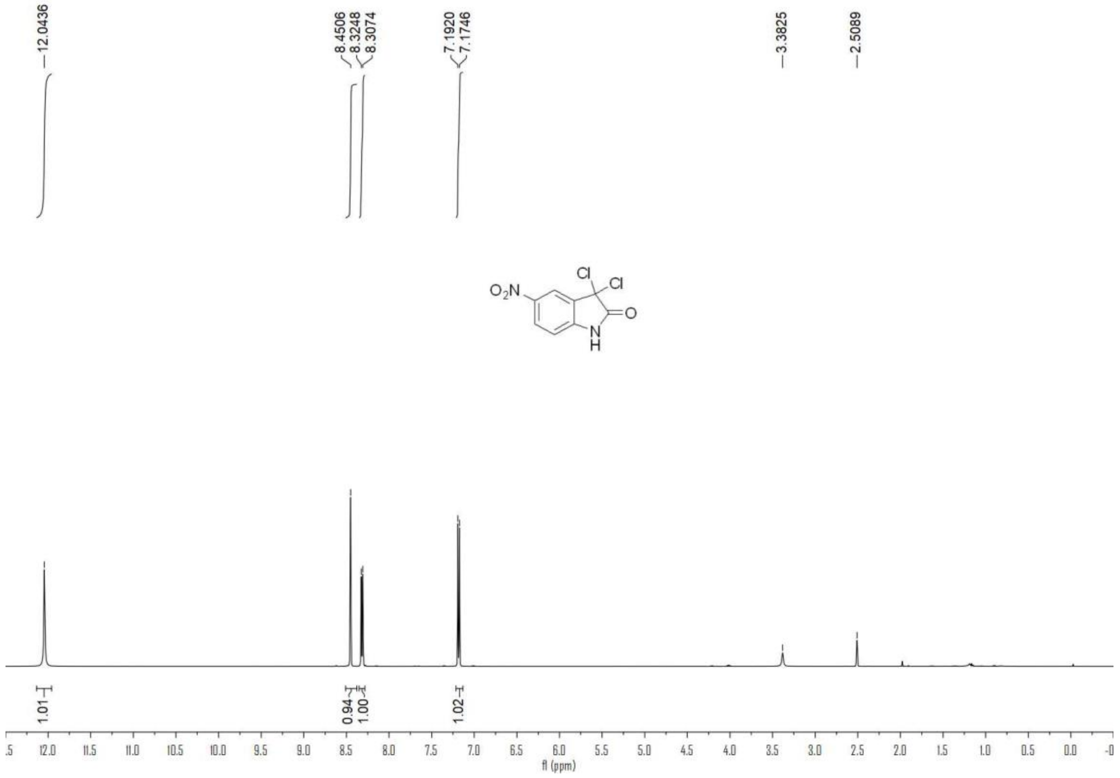

$^{13}C\{^1H\}$ NMR (126 MHz) of 5d in d_6-DMSO

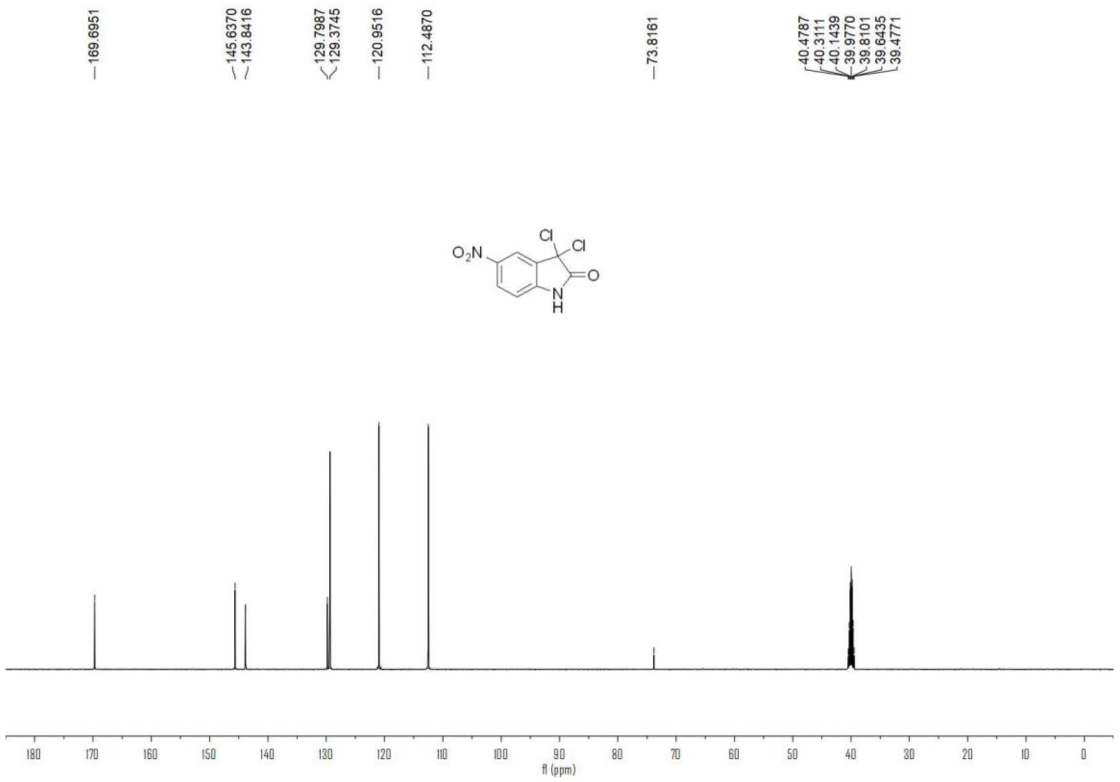

^1H NMR (500 MHz) of 5e in $CDCl_3$

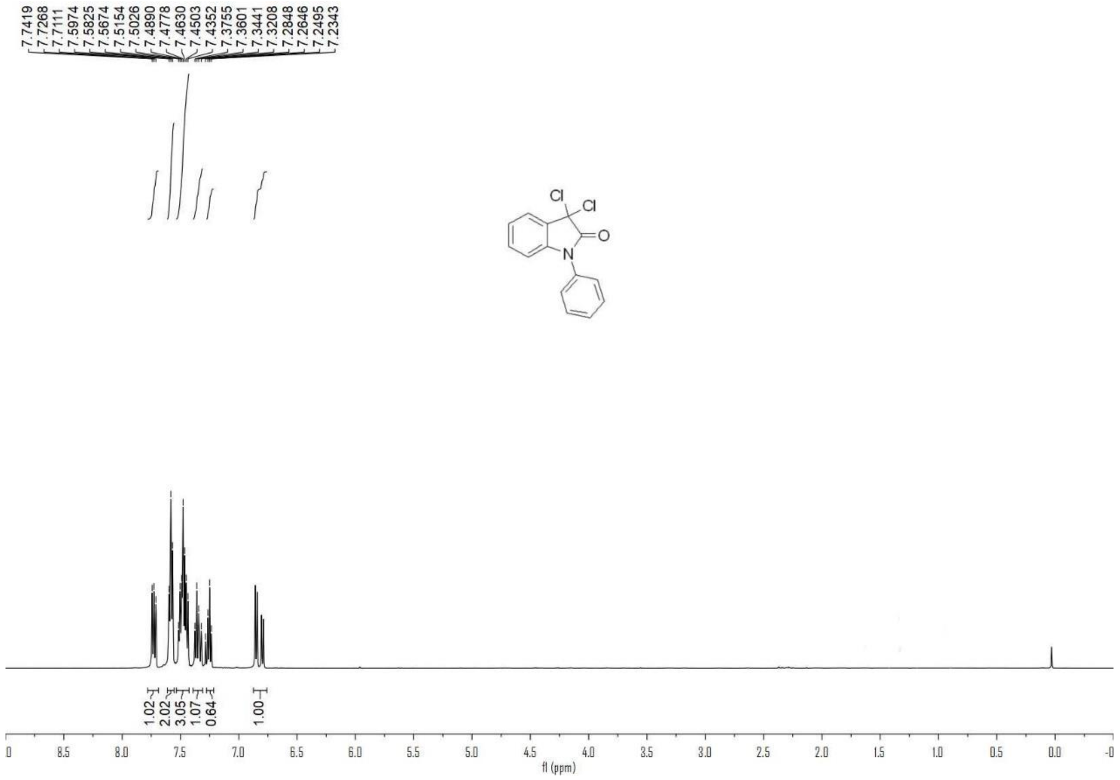

$^{13}C\{^{1}H\}$ NMR (126 MHz) of 5e in $CDCl_3$

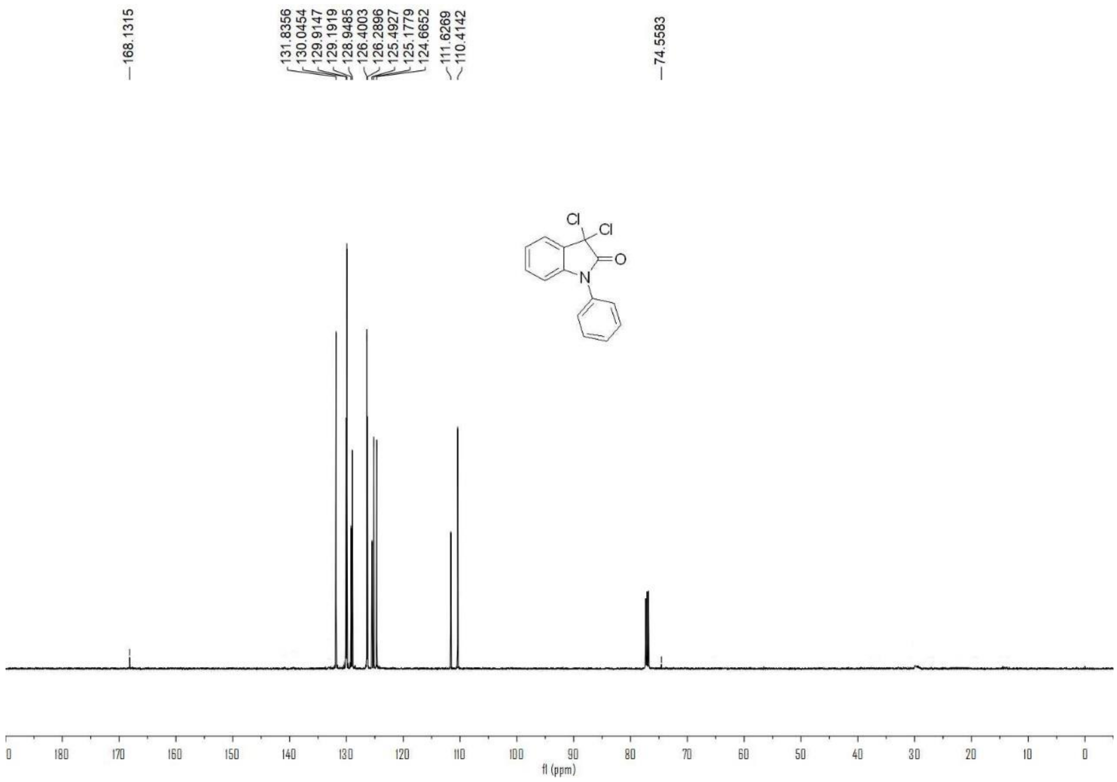

^{1}H NMR (600 MHz) of 7a in $CDCl_3$

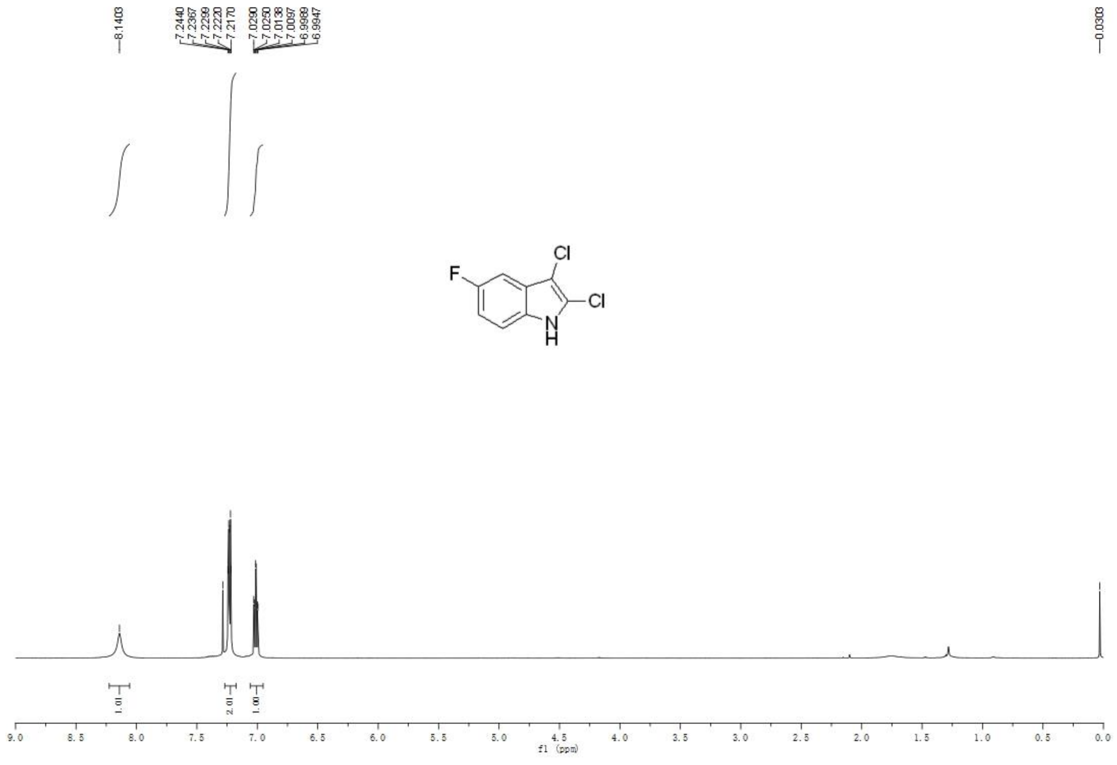

$^{13}C\{^1H\}$ NMR (151 MHz) of 7a in $CDCl_3$

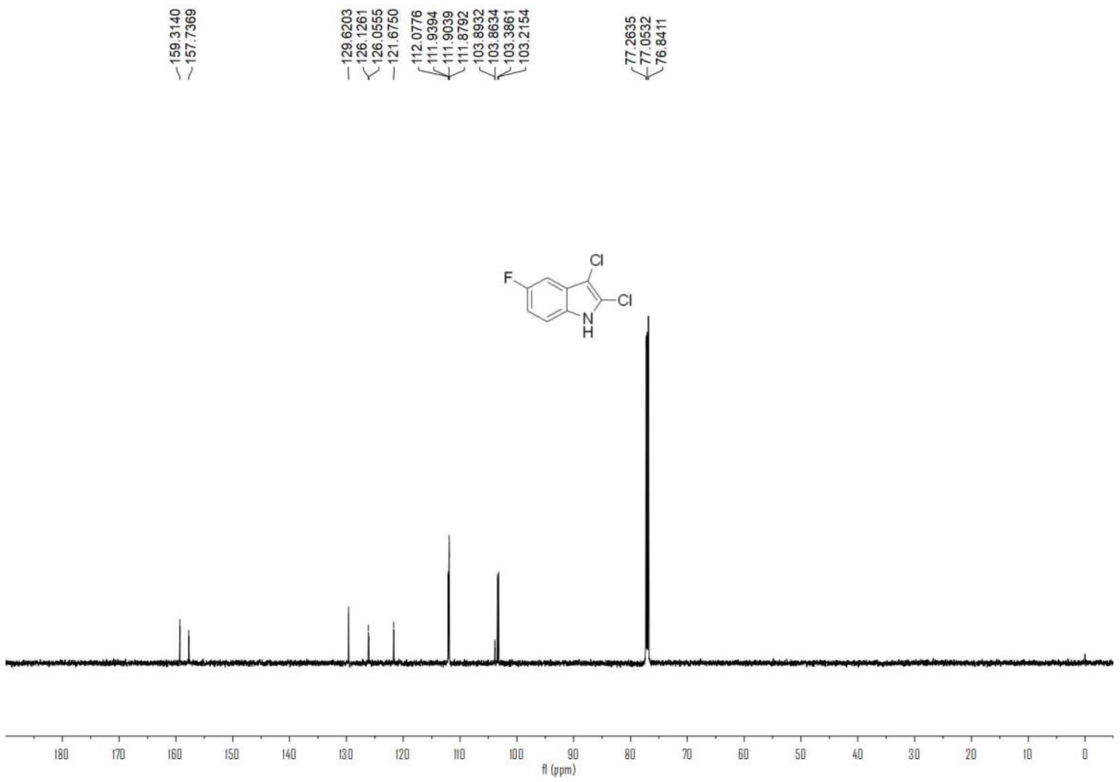

^{19}F NMR (565 MHz) of 7a in $CDCl_3$

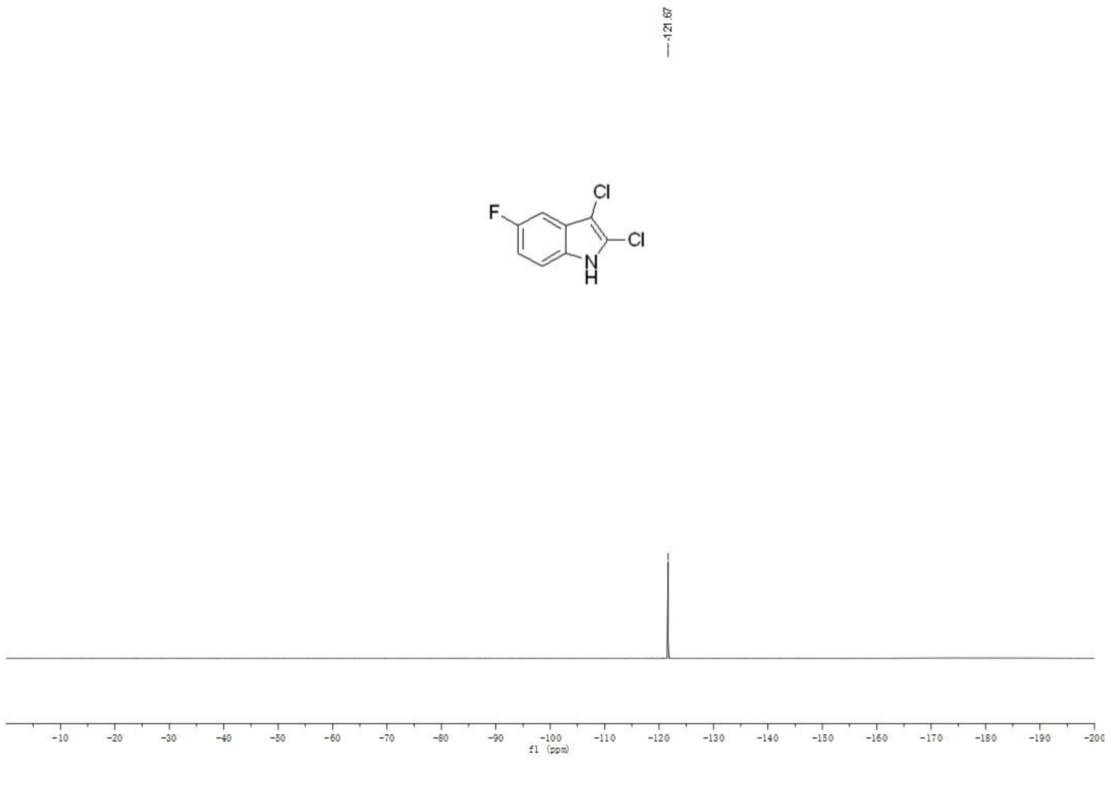

^{1}H NMR (600 MHz) of 7b in CDCl$_3$

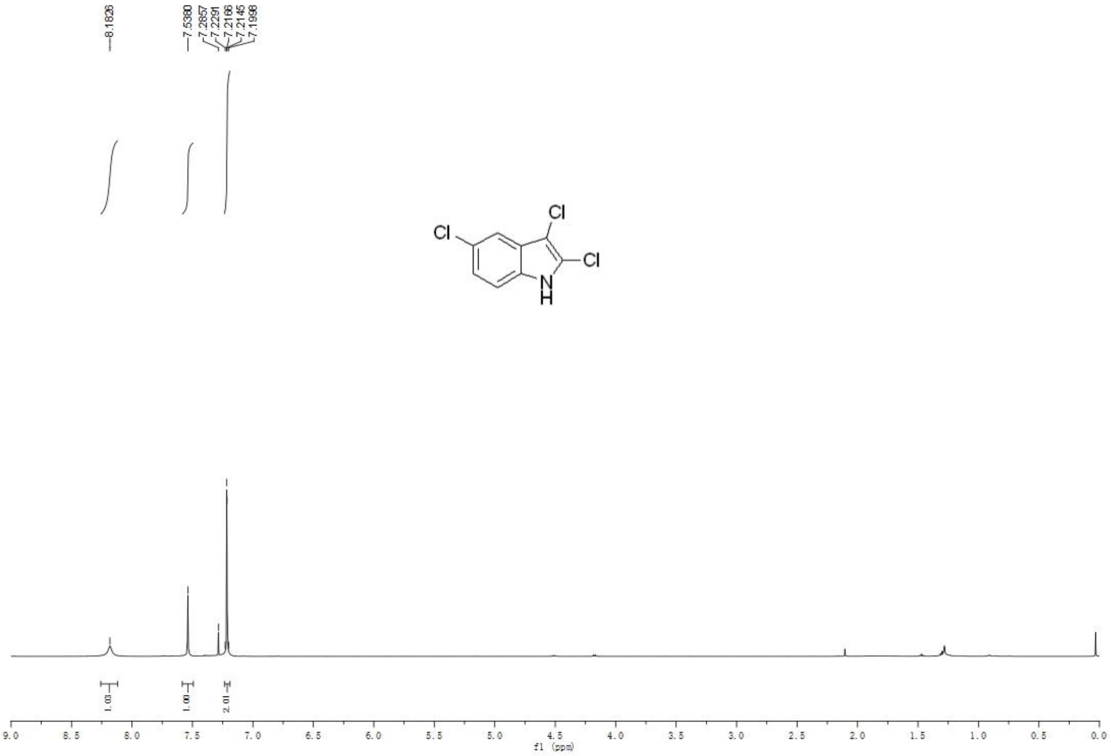

^{13}C{^{1}H} NMR (151 MHz) of 7b in CDCl$_3$

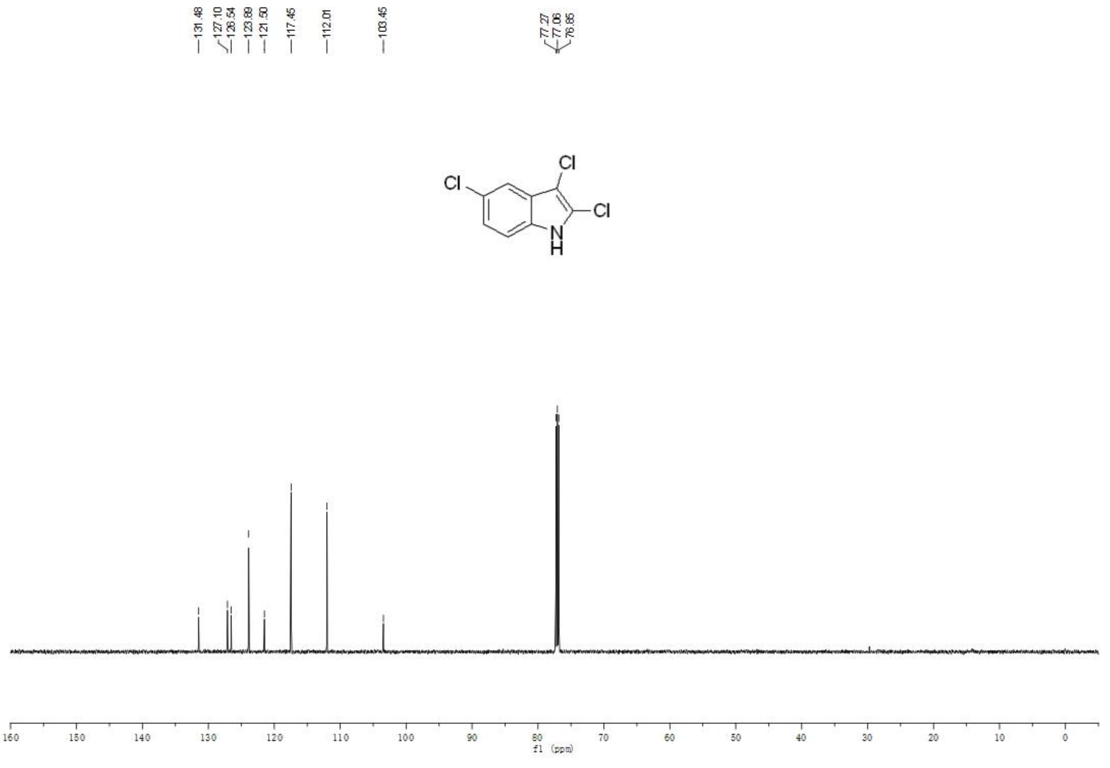

^{1}H NMR (600 MHz) of 7c in $CDCl_3$

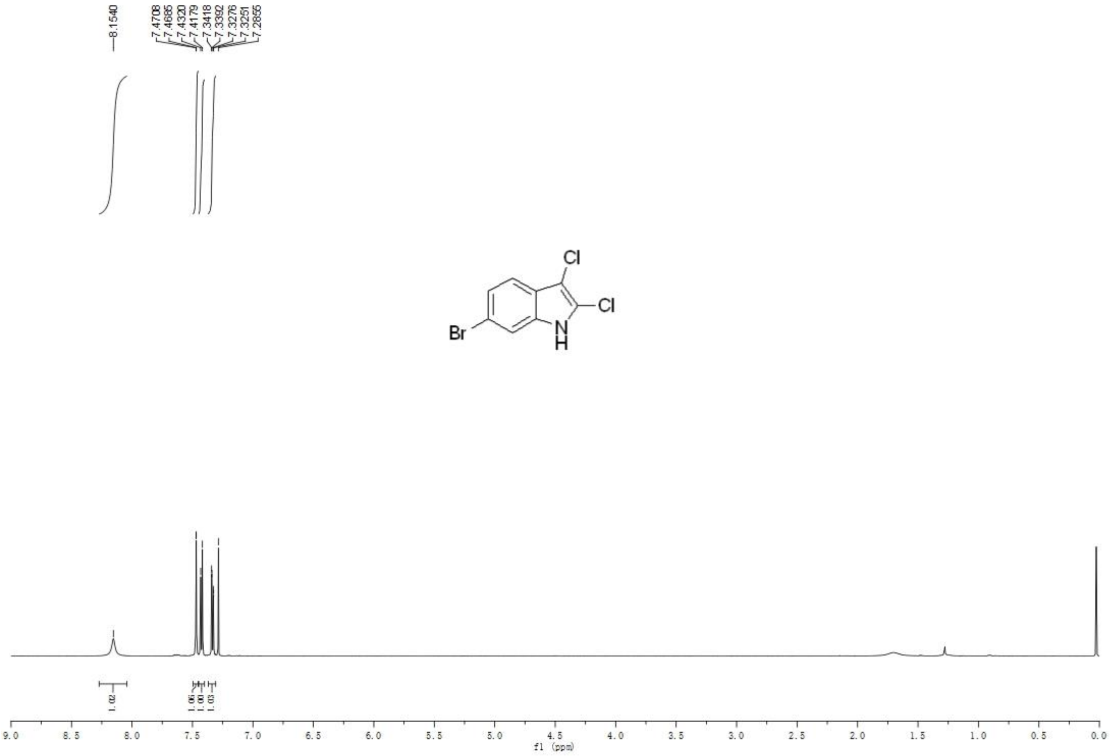

^{13}C{^{1}H} NMR (151 MHz) of 7c in $CDCl_3$

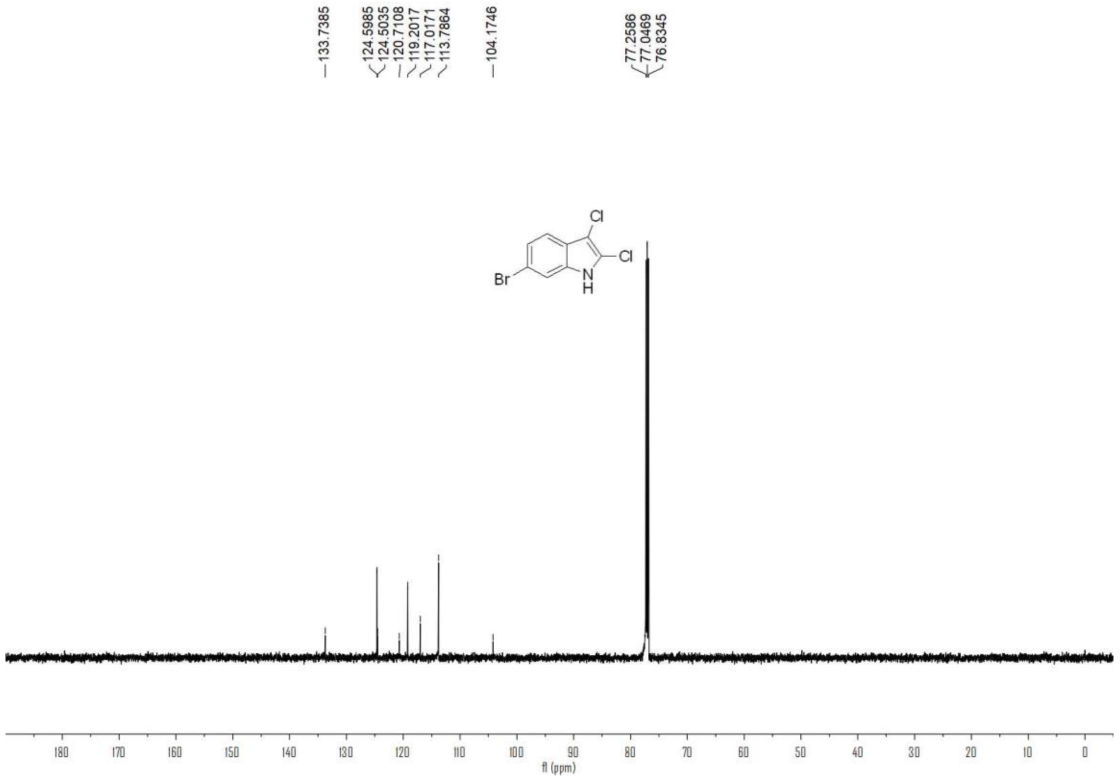

^{1}H NMR (500 MHz) of 8 in $CDCl_3$

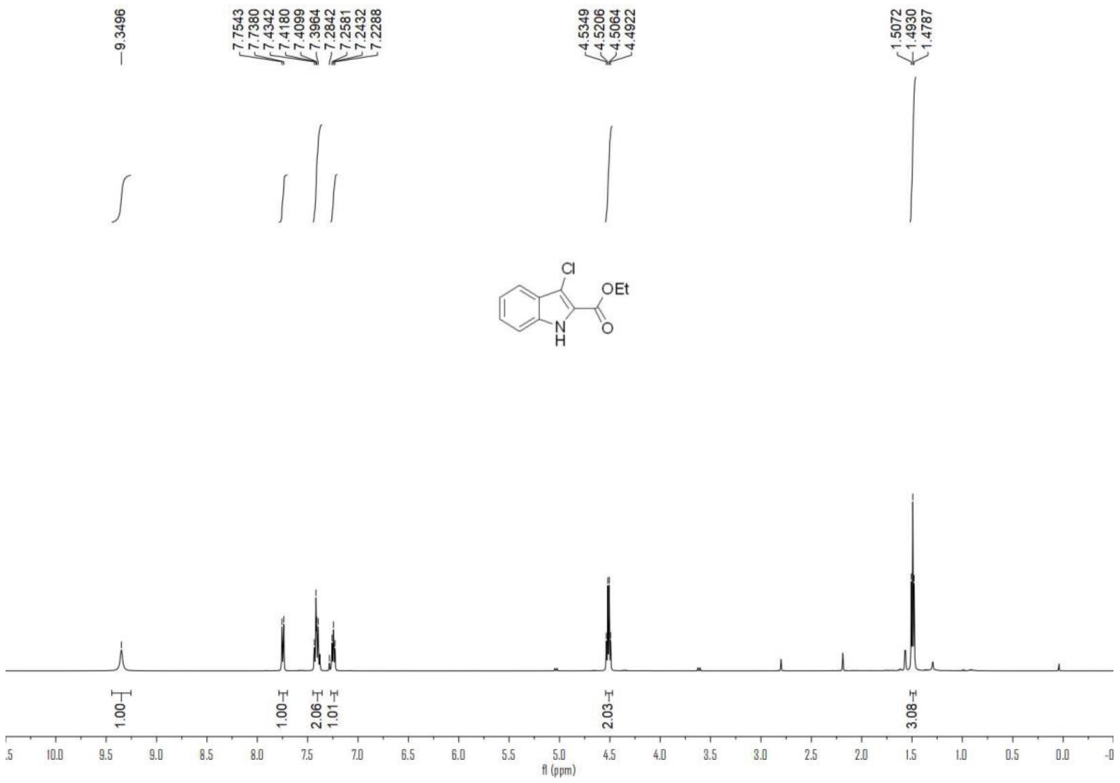

$^{13}C\{^{1}H\}$ NMR (126 MHz) of 8 in $CDCl_3$

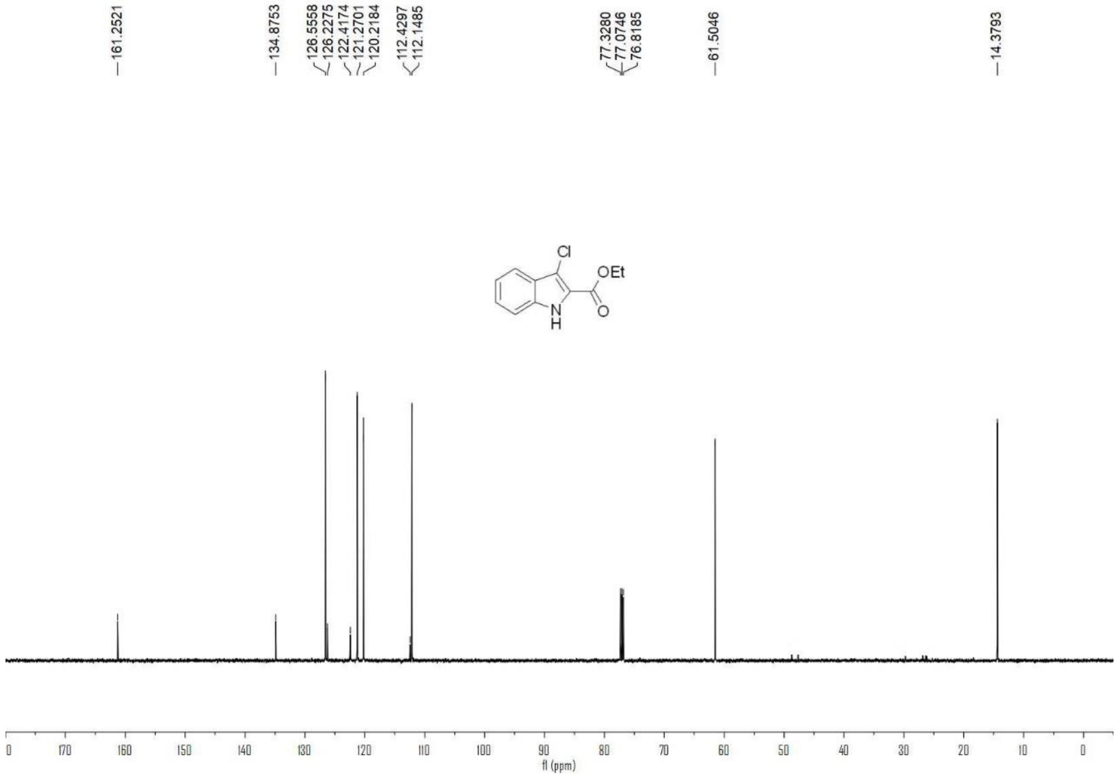

Supplement: Supplementary file 1 [file molecules-30-00102-s001.zip › molecules-3384517-supplementary.pdf]
